# Supplementary material for: Colchicine for the treatment of patients with COVID-19: an updated systematic review and meta-analysis of randomised controlled trials
Source: BMJ Open. 2024 Apr 17;14(4):e074373. doi: 10.1136/bmjopen-2023-074373 (PMC11029412; doi:10.1136/bmjopen-2023-074373)

Supplementary Material

Colchicine for the treatment of COVID-19 patients: a rapid systematic review and meta-analysis of randomized controlled trials

This supplemental material has been provided by the authors to give readers additional information about their work.

Table S1. PRISMA checklist.

Section and Topic	Item #	Checklist item	Reported on page #
**TITLE**			
Title	1	Identify the report as a systematic review.	1
**ABSTRACT**			
Abstract	2	See the PRISMA 2020 for Abstracts checklist.	3
**INTRODUCTION**			
Rationale	3	Describe the rationale for the review in the context of existing knowledge.	4
Objectives	4	Provide an explicit statement of the objective(s) or question(s) the review addresses.	4
**METHODS**			
Eligibility criteria	5	Specify the inclusion and exclusion criteria for the review and how studies were grouped for the syntheses.	5
Information sources	6	Specify all databases, registers, websites, organisations, reference lists and other sources searched or consulted to identify studies. Specify the date when each source was last searched or consulted.	5
Search strategy	7	Present the full search strategies for all databases, registers and websites, including any filters and limits used.	Suppl.
Selection process	8	Specify the methods used to decide whether a study met the inclusion criteria of the review, including how many reviewers screened each record and each report retrieved, whether they worked independently, and if applicable, details of automation tools used in the process.	5
Data collection process	9	Specify the methods used to collect data from reports, including how many reviewers collected data from each report, whether they worked independently, any processes for obtaining or confirming data from study investigators, and if applicable, details of automation tools used in the process.	5
Data items	10a	List and define all outcomes for which data were sought. Specify whether all results that were compatible with each outcome domain in each study were sought (e.g. for all measures, time points, analyses), and if not, the methods used to decide which results to collect.	5
	10b	List and define all other variables for which data were sought (e.g. participant and intervention characteristics, funding sources). Describe any assumptions made about any missing or unclear information.	5
Study risk of bias assessment	11	Specify the methods used to assess risk of bias in the included studies, including details of the tool(s) used, how many reviewers assessed each study and whether they worked independently, and if applicable, details of automation tools used in the process.	5,6
Effect measures	12	Specify for each outcome the effect measure(s) (e.g. risk ratio, mean difference) used in the synthesis or presentation of results.	6
Synthesis methods	13a	Describe the processes used to decide which studies were eligible for each synthesis (e.g. tabulating the study intervention characteristics and comparing against the planned groups for each synthesis (item #5)).	6

Section and Topic	Item #	Checklist item	Reported on page #
	13b	Describe any methods required to prepare the data for presentation or synthesis, such as handling of missing summary statistics, or data conversions.	6
	13c	Describe any methods used to tabulate or visually display results of individual studies and syntheses.	6
	13d	Describe any methods used to synthesize results and provide a rationale for the choice(s). If meta-analysis was performed, describe the model(s), method(s) to identify the presence and extent of statistical heterogeneity, and software package(s) used.	6
	13e	Describe any methods used to explore possible causes of heterogeneity among study results (e.g. subgroup analysis, meta-regression).	6
	13f	Describe any sensitivity analyses conducted to assess robustness of the synthesized results.	6
Reporting bias assessment	14	Describe any methods used to assess risk of bias due to missing results in a synthesis (arising from reporting biases).	6
Certainty assessment	15	Describe any methods used to assess certainty (or confidence) in the body of evidence for an outcome.	NA
**RESULTS**			
Study selection	16a	Describe the results of the search and selection process, from the number of records identified in the search to the number of studies included in the review, ideally using a flow diagram.	6, Figure 1
	16b	Cite studies that might appear to meet the inclusion criteria, but which were excluded, and explain why they were excluded.	Figure 1
Study characteristics	17	Cite each included study and present its characteristics.	6,7, Table 1
Risk of bias in studies	18	Present assessments of risk of bias for each included study.	7, Figure 2
Results of individual studies	19	For all outcomes, present, for each study: (a) summary statistics for each group (where appropriate) and (b) an effect estimate and its precision (e.g. confidence/credible interval), ideally using structured tables or plots.	Figures, suppl.
Results of syntheses	20a	For each synthesis, briefly summarise the characteristics and risk of bias among contributing studies.	7,8
	20b	Present results of all statistical syntheses conducted. If meta-analysis was done, present for each the summary estimate and its precision (e.g. confidence/credible interval) and measures of statistical heterogeneity. If comparing groups, describe the direction of the effect.	7,8
	20c	Present results of all investigations of possible causes of heterogeneity among study results.	7
	20d	Present results of all sensitivity analyses conducted to assess the robustness of the synthesized results.	7
Reporting	21	Present assessments of risk of bias due to missing results (arising from reporting biases) for each synthesis assessed.	7

Section and Topic	Item #	Checklist item	Reported on page #
biases			
Certainty of evidence	22	Present assessments of certainty (or confidence) in the body of evidence for each outcome assessed.	NA
**DISCUSSION**			
Discussion	23a	Provide a general interpretation of the results in the context of other evidence.	8-10
	23b	Discuss any limitations of the evidence included in the review.	9
	23c	Discuss any limitations of the review processes used.	9
	23d	Discuss implications of the results for practice, policy, and future research.	9,10
**OTHER INFORMATION**			
Registration and protocol	24a	Provide registration information for the review, including register name and registration number, or state that the review was not registered.	4
	24b	Indicate where the review protocol can be accessed, or state that a protocol was not prepared.	4
	24c	Describe and explain any amendments to information provided at registration or in the protocol.	NA
Support	25	Describe sources of financial or non-financial support for the review, and the role of the funders or sponsors in the review.	10
Competing interests	26	Declare any competing interests of review authors.	10
Availability of data, code and other materials	27	Report which of the following are publicly available and where they can be found: template data collection forms; data extracted from included studies; data used for all analyses; analytic code; any other materials used in the review.	10

Table S2. Search strategy for electronic databases.

**MEDLINE (PubMed)**	("colchicine"[MeSH Terms] OR "colchicine"[tiab]) AND ("COVID-19"[MeSH Terms] OR "COVID-19"[tiab] OR "SARS-CoV-2"[tiab] OR "coronavirus disease 2019"[tiab]) AND ("randomized controlled trial"[pt] OR "controlled clinical trial"[pt] OR "randomized"[tiab] OR "placebo"[tiab] OR "drug therapy"[Subheading] OR "therapeutics"[tiab] OR "treatment"[tiab] OR "intervention"[tiab] OR "clinical trial"[pt] OR "placebo-controlled trial"[tiab])
**Embase**	'colchicine'/exp OR 'colchicine':ab,ti AND ('COVID-19'/exp OR 'COVID-19':ab,ti OR 'SARS-CoV-2':ab,ti OR 'coronavirus disease 2019':ab,ti) AND ('clinical trial'/exp OR 'randomized controlled trial'/exp OR 'placebo'/exp OR 'double-blind procedure'/exp OR 'single-blind procedure'/exp OR 'clinical trial phase III'/exp OR 'clinical trial phase IV'/exp OR 'randomization'/exp OR 'placebo-controlled study'/exp OR 'crossover procedure'/exp OR 'factorial design'/exp OR 'control group'/exp)
**The Cochrane Library**	(("Colchicine"[Mesh]) OR ("colchicine"[tiab])) AND (("COVID-19"[Mesh]) OR ("SARS-CoV-2"[Mesh]) OR ("Coronavirus Disease 2019"[Mesh]) OR ("COVID-19"[tiab]) OR ("SARS-CoV-2"[tiab]) OR ("coronavirus disease 2019"[tiab]))

Table S3. Characteristics on included trials.

Sr No	Author, year	Country	Sample size	Age*	Sex, n (%)	Population	Intervention	Comparator	Immunization Status
1	Absalón-Aguilar 2021	Mexico	116	53 (44–62)	Male: 76 (65.5)<br>Female: 40 (35.5)	Hospitalized COVID-19 adult patients with severe disease	1.5 mg of colchicine at the time of the recruitment in the study and 0.5 mg BID to complete 10 days of treatment	Placebo	NR
2	Alsultan 2021	Syria	49 (35 were eligible as 14 received budesonide)	-	-	Hospitalized, moderate-to-severe ARDS, adult patients with either a positive PCR test of COVID-19 virus in specimens taken from the respiratory tracts or a negative PCR test but with clinical signs and symptoms of viral illness along with findings on chest CT compatible with COVID-19 infection	1.5 mg of colchicine followed by 0.5 mg after an hour on day 1, then 0.5 mg twice daily for the next 4 days	Supportive care only	NR
3	Cecconi 2022	Spain	239	65.1 ± 16.0	Male: 141 (59)<br>Female: 98 (41)	Hospitalized moderate-to-severe COVID-19 patients	5 days of oral colchicine: 1 mg loading dose and then 0.5 mg/day	Placebo	NR

4	Deftereos 2020	Greece	105	64 (54-76)	Male: 61 (58.1)<br>Female: 44 (41.9)	Hospitalized adult COVID-19 patients	The loading dose consisted of 1.5 mg of colchicine followed by 0.5 mg of colchicine 60 minutes later. The maintenance dosage was 0.5 mg colchicine twice daily until hospital discharge or a maximum of 21 days.	No colchicine	NR
5	Diaz 2021	Argentina	1279	61.8 ± 14.6	Male: 830 (64.9)<br>Female: 449 (35.1)	Hospitalized severe COVID-19 patients	Colchicine was administered orally in a loading dose of 1.5 mg followed by 0.5 mg orally within 2 hours of the initial dose and 0.5 mg orally twice a day for 14 days or discharge, whichever occurred first.	Usual care with no colchicine	NR
6	Dorward 2022	UK	2293 (156 received colchicine)	-	-	Non-hospitalized COVID-19 patients	Colchicine 500 µg daily for 14 days	Usual care	Both vaccinated and unvaccinated population
7	Eikelboom 2022 (inpatients)	11 countries	2611	Colchicine: 56.1 ± 16.7<br>Control: 56.0 ± 16.0	Colchicine: Male: 762 (58.4), Female: 542 (31.6)<br>Control: Male: 796 (60.9), Female: 511 (39.1)	Hospitalized COVID-19 patients	Colchicine 1.2 mg followed by 0.6 mg 2h later and then 0.6 mg twice daily in tablet form for 28 days	Usual care	Both vaccinated and unvaccinated population

8	Eikelboom 2022 (outpatients)	11 countries	3917	Colchicine: 45.0 ± 13.7 Control: 45.0 ± 13.3	Colchicine: Male: 1173 (60.5%) Female 766 (39.5%) Control: Male: 1177 (60·6%) Female 765 (39.4%)	Non-hospitalized COVID-19 patients	Oral colchicine 0.6 mg twice daily for 3 days and then 0.6 mg once daily for 25 days	Usual care	Both vaccinated and unvaccinated population
9	Gaitan-Duarte 2021	Bogota, Colombia	314	Colchicine + Rosuvastatin: 56.1 ± 13.2 Control: 55.3 ± 12.3	Colchicine: Male: 103 (68), Female: 50 (32) Standard care: Male 113 (70.2), Female 48 (29.8)	Hospitalized adult COVID-19 patients	Colchicine plus rosuvastatin (0.5 mg and 40 mg for 14 days)	Standard of care	NR
10	Gorial 2022	Iraq	160	-	-	Hospitalized moderate-to-severe COVID-19 patients	Colchicine 1 mg daily orally for 7 days, then 0.5 mg daily for another 7 days	Standard treatment	NR
11	Jalal 2022	Iraq	80	-	Colchicine: Male: 21 (52.5), Female: 19 (47.5) Control: Male: 19 (47.5), Female: 21 (52.5)	Patients with mild, moderate, or severe COVID-19 infection; either hospitalized or at home	Colchicine 0.5 mg twice daily for 14 days	Standard treatment	NR
12	Kasiri 2023	Iran	106	54.62 ± 13.92	Male: 49 (46.2) Female: 57 (53.8)	Hospitalized adult severe COVID-19 patients	Colchicine 2 mg loading dose followed by 0.5 mg twice daily for 7 days	Placebo	NR
13	Lopes 2021	Brazil	75	Colchicine group: 54.5 (42.5–64.5) Placebo: 55.0 (42.0–67.0)	Colchicine: Male 19 (53), Female: 17 (47) Placebo: Male: 14 (39), Female: 22 (61)	Hospitalized moderate-to-severe COVID-19 patients	Colchicine 0.5 mg thrice daily for 5 days, then 0.5mg twice daily for 5 days	Placebo	NR

14	Mostafaie, unpublished	Iran	120	-	-	Hospitalized COVID-19 patients aged ≥ 10 years	Colchicine plus herbal phenolic monoterpene	Standard treatment	NR
15	Pascual-Figal 2021	Spain	103	51 ± 12	Male: 54 (52.4) Female: 49 (47.6)	Hospitalized adult COVID-19 patients	Colchicine 1.5 mg loading dose, followed by 0.5 mg BID for one week and 0.5 mg per day for 28 days	Standard treatment	NR
16	Perricone 2023	Italy	152	Colchicine: 69.1 ± 13.1 Control: 67.9 ± 15	Colchicine: Male: 47 (61), Female: 30 (39) Control: Male: 50 (67), Female: 25 (33)	Hospitalized adult COVID-19 patients	Colchicine 0.5 mg three times a day if weight was less than 100 kg or 1 mg twice a day if weight was more than 100 kg for a maximum of 30 days or until hospital discharge	Standard treatment	Unvaccinated population only
17	Pimenta Bonifácio 2022	Brazil	30	Colchicine: 56.22 ± 11.6 Control: 46.11 ± 10.6	Colchicine: Male: 10 (71), Female: 4 (29) Control: Male: 11 (69), Female 5 (31)	Hospitalized moderate-to-severe COVID-19 patients	Colchicine 0.5 mg every 8 hours for three days, followed by 0.5mg 2 complete four weeks of treatment	Standard of care	Unvaccinated population only
18	Pourdowlat 2021	Iran	165	54.72 ± 15.03	Male: 93 (63) Female: 72 (37)	Non-hospitalized moderate-to-severe COVID-19 patients	A 3-day course of 0.5 mg colchicine followed by a 12-day course of 1 mg colchicine	Standard of care	NR

19	Rahman 2022	Bangladesh	299	47 (35–55)	Male: 190 (64.2) Female: 106 (35.8)	Hospitalized moderate COVID-19 patients	1.2 mg of colchicine on day 1 followed by daily treatment with 0.6 mg of colchicine for 13 days	Placebo	NR
20	RECOVERY 2021	UK, Indonesia, Nepal	11340	63.4 ± 13·8	Colchicine: Male: 3897 (69), Female: 1713 (31) Control group: Male: 4012 (70), Female: 1718 (30)	Hospitalized moderate-to-severe adult COVID-19 patients	Colchicine 1 mg followed by 500 µg 12h later and then 500 µg twice a day by mouth or nasogastric tube for 10 days in total or until discharge	Standard of care	NR
21	Salehzadeh 2022	Iran	100	Colchicine: 56.56 ± 17.16 Placebo: 55.56 ± 16.38	Male: 41 (41) Female 59 (59)	Hospitalized moderate COVID-19 patients	Colchicine 1 mg daily for six days	Placebo	NR
22	Sunil Naik 2023	India	122	-	-	Hospitalized moderate COVID-19 patients	Colchicine 0.5 mg BID	Standard care	NR
23	Tardif 2021	Brazil, Canada, Greece, South Africa, Spain, and the USA	4488	54·0 (47·0–61·0)	Colchicine: Males: 997 (44.6), Females: 1238 (55.4) Control: Males: 1070 (47.5), Female: 1183 (52.5)	Non-hospitalized COVID-19 patients aged ≥ 40 years	Colchicine 0·5 mg BID for the first 3 days and then once per day for 27 days	Placebo	NR

*Data reported as mean ± SD and median (IQR)

BID, twice daily; ARDS, acute respiratory distress syndrome; PCR, polymerase chain reaction: NR, Not reported

Supplementary Figure S1. Funnel plot for all-cause mortality.

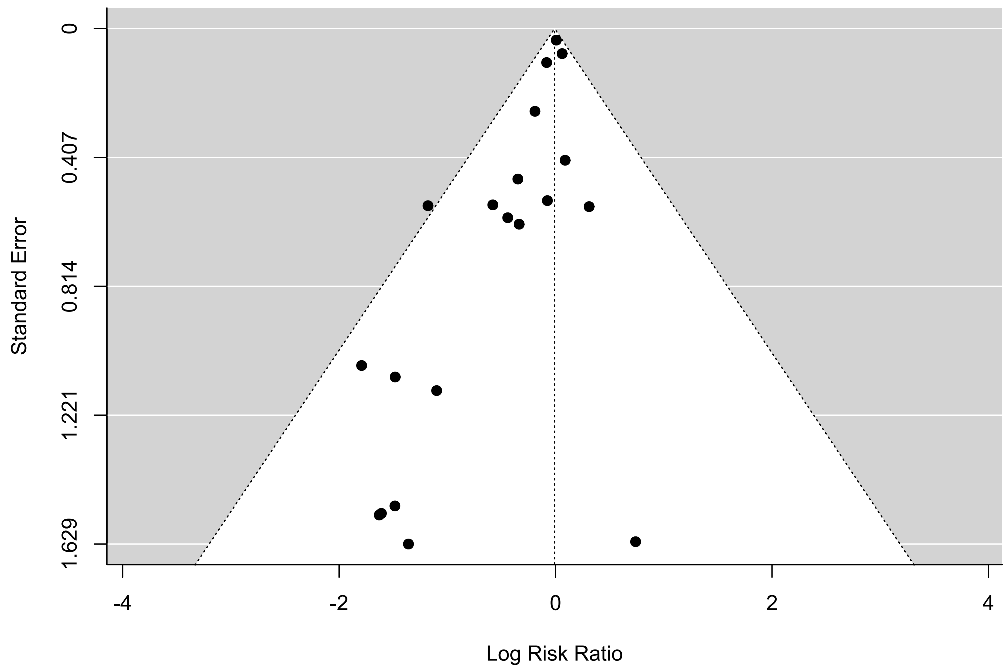

Supplementary Figure S2. Subgroup analysis of the effect of colchicine on the risk of mortality.

Study or Subgroup	Colchicine Events	Colchicine Total	Control Events	Control Total	Weight	Risk Ratio IV, Random, 95% CI
**1.6.1 Hospitalized**						
Absalon-Aguilar 2021	4	56	6	60	0.3%	0.71 [0.21, 2.40]
Alsultan 2021	3	14	7	21	0.3%	0.64 [0.20, 2.07]
Cecconi 2022	7	119	10	120	0.4%	0.71 [0.28, 1.79]
Deftereos 2020	1	55	4	50	0.1%	0.23 [0.03, 1.97]
Diaz 2021	131	640	142	639	8.3%	0.92 [0.75, 1.14]
Eikelboom 2022 (Inpatient)	264	1304	249	1307	15.3%	1.06 [0.91, 1.24]
Gaitan-Duarte 2021	22	153	28	161	1.4%	0.83 [0.50, 1.38]
Gorial 2022	1	80	3	80	0.1%	0.33 [0.04, 3.14]
Kasiri 2023	6	55	6	51	0.3%	0.93 [0.32, 2.69]
Lopes 2021	0	36	2	36	0.0%	0.20 [0.01, 4.03]
Mostafaie 2021	1	60	6	60	0.1%	0.17 [0.02, 1.34]
Pascual-Figal 2021	0	52	2	51	0.0%	0.20 [0.01, 3.99]
Perricone 2022	7	77	5	75	0.3%	1.36 [0.45, 4.11]
Pimenta-Bonifacio 2022	0	14	2	16	0.0%	0.23 [0.01, 4.36]
Rahman 2022	4	146	13	146	0.3%	0.31 [0.10, 0.92]
RECOVERY 2021	1173	5610	1190	5730	71.8%	1.01 [0.94, 1.08]
Sunil 2023	1	62	0	43	0.0%	2.10 [0.09, 50.25]
**Subtotal (95% CI)**		**8533**		**8646**	**99.1%**	**0.98 [0.90, 1.06]**
Total events	1625		1675			
Heterogeneity: Tau² = 0.00; Chi² = 16.79, df = 16 (P = 0.40); I² = 5%						
Test for overall effect: Z = 0.47 (P = 0.64)						
**1.6.2 Non-hospitalized**						
Dorward 2022	0	156	1	120	0.0%	0.26 [0.01, 6.25]
Eikelboom 2022 (Outpatient)	12	1939	11	1942	0.6%	1.09 [0.48, 2.47]
Tardif 2021	5	2235	9	2253	0.3%	0.56 [0.19, 1.67]
**Subtotal (95% CI)**		**4330**		**4315**	**0.9%**	**0.82 [0.43, 1.55]**
Total events	17		21			
Heterogeneity: Tau² = 0.00; Chi² = 1.45, df = 2 (P = 0.48); I² = 0%						
Test for overall effect: Z = 0.61 (P = 0.54)						
**Total (95% CI)**		**12863**		**12961**	**100.0%**	**0.99 [0.93, 1.05]**
Total events	1642		1696			
Heterogeneity: Tau² = 0.00; Chi² = 18.59, df = 19 (P = 0.48); I² = 0%						
Test for overall effect: Z = 0.28 (P = 0.78)						
Test for subgroup differences: Chi² = 0.30, df = 1 (P = 0.59), I² = 0%						

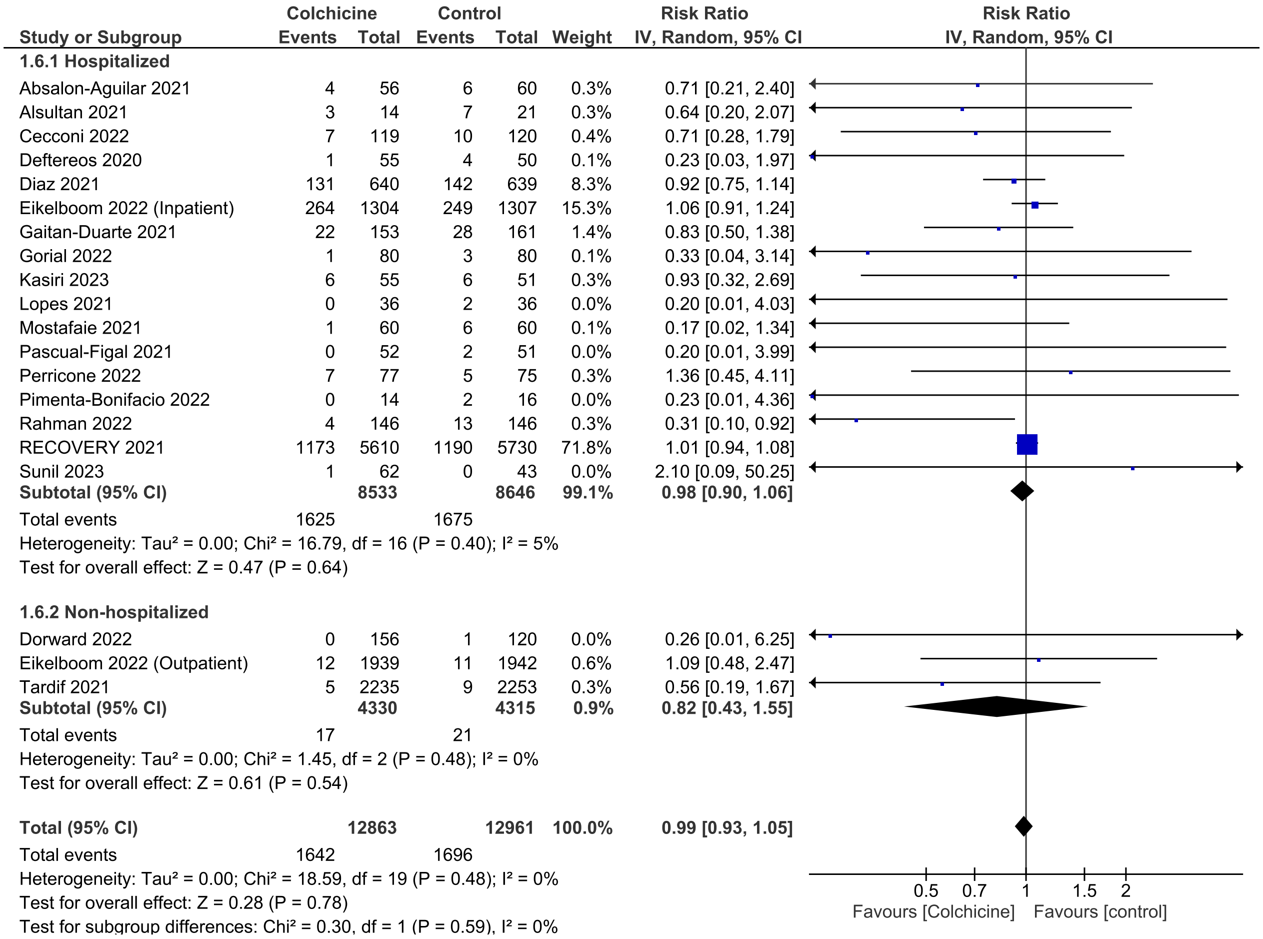

Supplementary Figure S3. Forest plot of the effect of colchicine on the risk of mechanical ventilation and ICU admission.

Study or Subgroup	Colchicine Events	Colchicine Total	Control Events	Control Total	Weight	Risk Ratio IV, Random, 95% CI
**1.2.1 Risk of mechanical ventilation**						
Cecconi 2022	5	119	10	120	12.5%	0.50 [0.18, 1.43]
Deftereos 2020	1	55	5	50	4.0%	0.18 [0.02, 1.50]
Kasiri 2023	6	55	6	51	12.2%	0.93 [0.32, 2.69]
Pascual-Figal 2021	0	52	2	51	2.1%	0.20 [0.01, 3.99]
Perricone 2022	4	77	3	75	7.6%	1.30 [0.30, 5.61]
Rahman 2022	2	146	4	146	6.0%	0.50 [0.09, 2.69]
RECOVERY 2021	259	3815	228	3962	36.4%	1.18 [0.99, 1.40]
Tardif 2021	11	2235	21	2253	19.2%	0.53 [0.26, 1.09]
**Subtotal (95% CI)**		**6554**		**6708**	**100.0%**	**0.75 [0.48, 1.18]**
Total events	288		279			
Heterogeneity: Tau² = 0.14; Chi² = 11.69, df = 7 (P = 0.11); I² = 40%						
Test for overall effect: Z = 1.23 (P = 0.22)						
**1.2.2 ICU admission**						
Cecconi 2022	11	119	14	120	37.4%	0.79 [0.38, 1.67]
Kasiri 2023	12	55	9	51	34.7%	1.24 [0.57, 2.69]
Lopes 2021	2	36	4	36	7.8%	0.50 [0.10, 2.56]
Pascual-Figal 2021	2	52	4	51	7.7%	0.49 [0.09, 2.56]
Perricone 2022	1	77	4	75	4.4%	0.24 [0.03, 2.13]
Rahman 2022	2	146	5	146	7.9%	0.40 [0.08, 2.03]
**Subtotal (95% CI)**		**485**		**479**	**100.0%**	**0.77 [0.49, 1.22]**
Total events	30		40			
Heterogeneity: Tau² = 0.00; Chi² = 3.70, df = 5 (P = 0.59); I² = 0%						
Test for overall effect: Z = 1.10 (P = 0.27)						

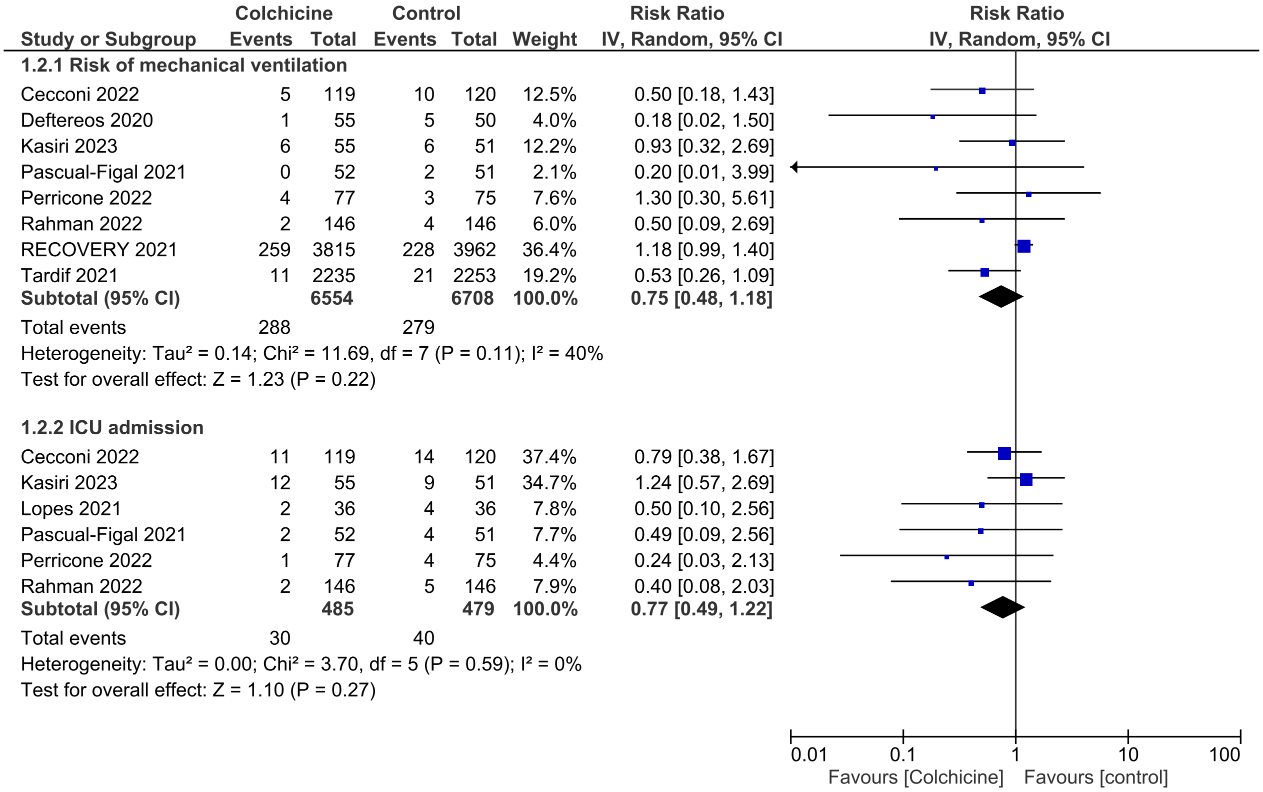

Supplementary Figure S4. Forest plot of the effect of colchicine on the risk of hospitalization.

	Colchicine		Control			Risk Ratio
**Study or Subgroup**	**Events**	**Total**	**Events**	**Total**	**Weight**	**IV, Random, 95% CI**
Eikelboom 2022 (Outpatient)	62	1939	61	1942	39.7%	1.02 [0.72, 1.44]
Pourdowlat 2021	5	102	18	100	15.5%	0.27 [0.11, 0.71]
Tardif 2021	101	2235	128	2253	44.8%	0.80 [0.62, 1.03]
**Total (95% CI)**		**4276**		**4295**	**100.0%**	**0.74 [0.48, 1.16]**
Total events	168		207			

Heterogeneity: Tau² = 0.10; Chi² = 6.69, df = 2 (P = 0.04); I² = 70%
Test for overall effect: Z = 1.30 (P = 0.19)

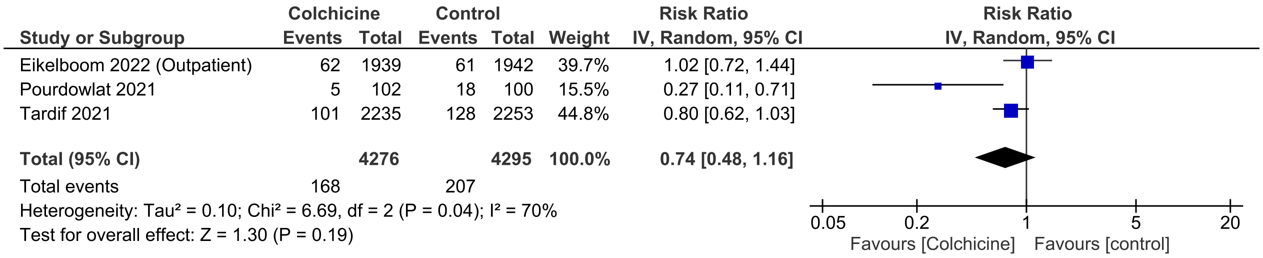

Supplementary Figure S5. Forest plot of the effect of colchicine on the length of hospital stay.

	Colchicine			Control				Mean Difference
**Study or Subgroup**	**Mean**	**SD**	**Total**	**Mean**	**SD**	**Total**	**Weight**	**IV, Random, 95% CI**
Absalon-Aguilar 2021	7.916	4.26	56	8.33	4.07	60	13.2%	-0.41 [-1.93, 1.10]
Jalal 2022	18.4	5.81	40	24.24	8.63	40	9.3%	-5.84 [-9.06, -2.62]
Lopes 2021	7	2.96	36	9.33	3.7	36	13.1%	-2.33 [-3.88, -0.78]
Mostafaie 2021	6.39	2.59	54	4.17	1.34	59	14.4%	2.22 [1.45, 2.99]
Pascual-Figal 2021	6.6	3.86	52	5.76	4.89	51	12.8%	0.84 [-0.86, 2.54]
Perricone 2022	14.1	10.4	77	14.7	8.1	75	9.9%	-0.60 [-3.56, 2.36]
Rahman 2022	10.67	5.93	146	10	6.67	146	13.3%	0.67 [-0.78, 2.12]
Salehzadeh 2022	6.28	2.51	50	8.12	2.66	50	14.1%	-1.84 [-2.85, -0.83]
**Total (95% CI)**			**511**			**517**	**100.0%**	**-0.70 [-2.30, 0.89]**

Heterogeneity: Tau² = 4.44; Chi² = 67.53, df = 7 (P < 0.00001); I² = 90%
Test for overall effect: Z = 0.86 (P = 0.39)

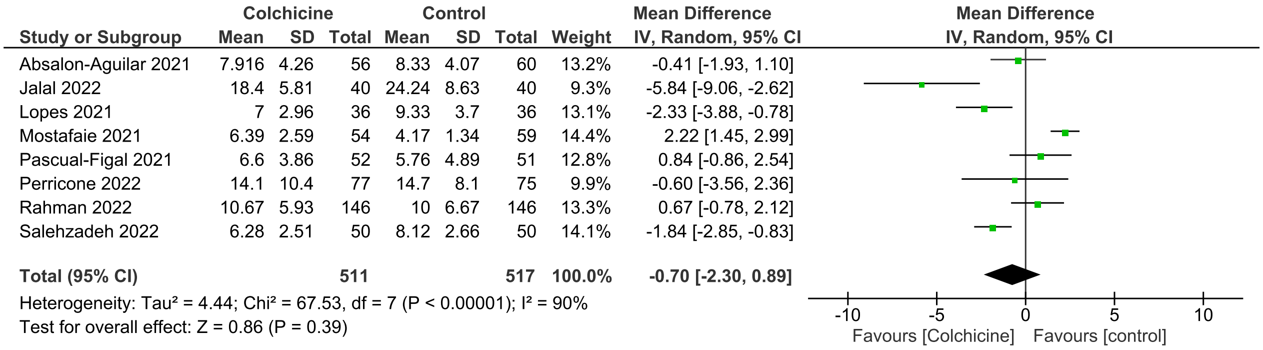

Supplementary Figure S6. Forest plot of the effect of colchicine on the rate of no recovery.

	Colchicine		Control			Risk Ratio
**Study or Subgroup**	**Events**	**Total**	**Events**	**Total**	**Weight**	**IV, Random, 95% CI**
Dorward 2022	42	156	306	1145	25.3%	1.01 [0.76, 1.33]
Gorial 2022	12	80	32	80	14.8%	0.38 [0.21, 0.67]
Kasiri 2023	7	55	9	51	8.5%	0.72 [0.29, 1.79]
Pourdowlat 2021	25	102	40	100	20.1%	0.61 [0.40, 0.93]
RECOVERY 2021	1709	5610	1698	5730	31.3%	1.03 [0.97, 1.09]
**Total (95% CI)**		**6003**		**7106**	**100.0%**	**0.77 [0.56, 1.05]**
Total events	1795		2085			

Heterogeneity: Tau² = 0.08; Chi² = 17.39, df = 4 (P = 0.002); I² = 77%
Test for overall effect: Z = 1.65 (P = 0.10)

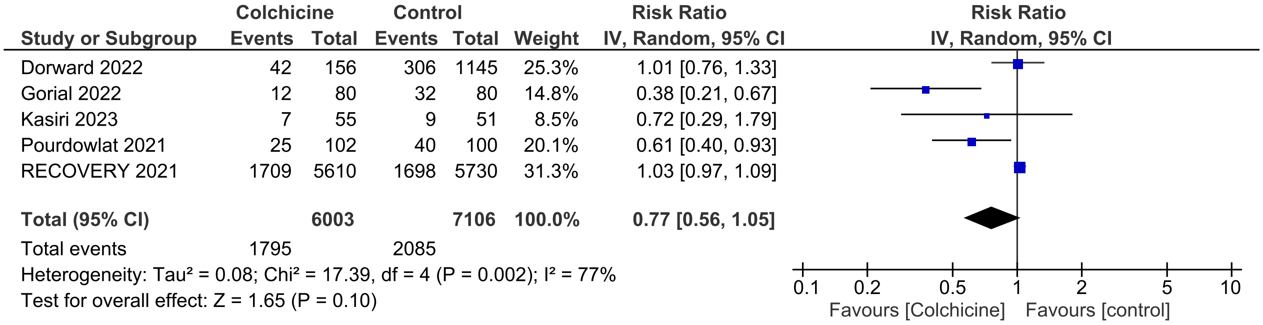

Supplement: Supplementary data [file bmjopen-2023-074373supp001.pdf]
