# Supplementary material for: Colchicine for the treatment of patients with COVID-19: an updated systematic review and meta-analysis of randomised controlled trials
Source: BMJ Open. 2024 Apr 17;14(4):e074373. doi: 10.1136/bmjopen-2023-074373 (PMC11029412; doi:10.1136/bmjopen-2023-074373)
Supplement: Author's manuscript [file bmjopen-2023-074373.draft_revisions.pdf]

BMJ Open is committed to open peer review. As part of this commitment we make the peer review history of every article we publish publicly available.

When an article is published we post the peer reviewers' comments and the authors' responses online. We also post the versions of the paper that were used during peer review. These are the versions that the peer review comments apply to.

The versions of the paper that follow are the versions that were submitted during the peer review process. They are not the versions of record or the final published versions. They should not be cited or distributed as the published version of this manuscript.

BMJ Open is an open access journal and the full, final, typeset and author-corrected version of record of the manuscript is available on our site with no access controls, subscription charges or pay-per-view fees (http://bmjopen.bmj.com).

If you have any questions on BMJ Open's open peer review process please email info.bmjopen@bmj.com

BMJ Open

Colchicine for the treatment of COVID-19 patients: a rapid systematic review and meta-analysis of randomized controlled trials

Journal:	*BMJ Open*
Manuscript ID	bmjopen-2023-074373
Article Type:	Original research
Date Submitted by the Author:	06-Apr-2023
Complete List of Authors:	Cheema, Huzaifa Ahmad; King Edward Medical University, Department of Medicine<br>Jafar, Uzair; King Edward Medical University, Department of Medicine<br>Usman, Muhammad; King Edward Medical University, Department of Medicine<br>Masood, Waniyah; Dow University of Health Sciences, Department of Medicine<br>Naseem, Muhammad Arsal; King Edward Medical University, Department of Medicine<br>Shahid, Abia; King Edward Medical University, Department of Medicine<br>Hermis, Alaa Hamza; Al-Mustaqbal University College<br>Sahra, Syeda; The University of Oklahoma Health Sciences Center, Department of Infectious Diseases<br>Sah, Ranjit; Tribhuvan University Teaching Hospital; Harvard Medical School<br>Lee, Ka Yiu; Mid Sweden University, Department of Health Sciences
Keywords:	COVID-19, SARS-CoV-2, VIROLOGY, CLINICAL PHARMACOLOGY

SCHOLARONE™
Manuscripts

I, the Submitting Author has the right to grant and does grant on behalf of all authors of the Work (as defined in the below author licence), an exclusive licence and/or a non-exclusive licence for contributions from authors who are: i) UK Crown employees; ii) where BMJ has agreed a CC-BY licence shall apply, and/or iii) in accordance with the terms applicable for US Federal Government officers or employees acting as part of their official duties; on a worldwide, perpetual, irrevocable, royalty-free basis to BMJ Publishing Group Ltd ("BMJ") its licensees and where the relevant Journal is co-owned by BMJ to the co-owners of the Journal, to publish the Work in this journal and any other BMJ products and to exploit all rights, as set out in our licence.

The Submitting Author accepts and understands that any supply made under these terms is made by BMJ to the Submitting Author unless you are acting as an employee on behalf of your employer or a postgraduate student of an affiliated institution which is paying any applicable article publishing charge ("APC") for Open Access articles. Where the Submitting Author wishes to make the Work available on an Open Access basis (and intends to pay the relevant APC), the terms of reuse of such Open Access shall be governed by a Creative Commons licence – details of these licences and which Creative Commons licence will apply to this Work are set out in our licence referred to above.

Other than as permitted in any relevant BMJ Author's Self Archiving Policies, I confirm this Work has not been accepted for publication elsewhere, is not being considered for publication elsewhere and does not duplicate material already published. I confirm all authors consent to publication of this Work and authorise the granting of this licence.

For peer review only

Colchicine for the treatment of COVID-19 patients: a rapid systematic review and meta-analysis of randomized controlled trials

Running title: Colchicine for COVID-19 patients

Huzaifa Ahmad Cheema*[1]; Uzair Jafar*[1]; Muhammad Usman[1]; Waniyah Masood[2]; Muhammad Arsal Naseem[1]; Abia Shahid[1]; Alaa Hamza Hermis[3]; Syeda Sahra[4]; Ranjit Sah[5,6]; Ka Yiu Lee[7]

*These authors contributed equally and are co-first authors.

1. Division of Infectious Diseases, Department of Medicine, King Edward Medical University, Lahore, Pakistan.
2. Department of Medicine, Dow University of Health Sciences, Karachi, Pakistan.
3. Al-Mustaqbal University College, 51001 Hillah, Babylon, Iraq.
4. Department of Infectious Diseases, University of Oklahoma Health Sciences Center, Oklahoma City, OK, United States.
5. Tribhuvan University Teaching Hospital, Institute of Medicine, Kathmandu, Nepal.
6. Harvard Medical School, Boston, MA, United States.
7. Swedish Winter Sports Research Centre, Department of Health Sciences, Mid Sweden University, Östersund, Sweden.

Corresponding authors:

1) Ka Yiu Lee, PhD; kyle.lee@miun.se

Address: Swedish Winter Sports Research Centre, Department of Health Sciences, Mid Sweden University, Östersund, Sweden

For peer review only

Telephone: +46725816133

2) Huzaifa Ahmad Cheema, MBBS: huzaifacheema@kemu.edu.pk;

Address: Division of Infectious Diseases, Department of Medicine, King Edward Medical University, Nila Gumbad Chowk, Neela Gumbad Lahore, Punjab 54000

Telephone: +923324487886

Word count: 1592

For peer review only

Abstract

Objectives: Colchicine is being extensively investigated among COVID-19 patients. The results of previous meta-analyses and randomized controlled trials (RCTs) have been inconsistent. Hence, we conducted an updated meta-analysis to investigate the effect of colchicine treatment on clinical outcomes in COVID-19 patients.

Design: We searched PubMed, Embase, the Cochrane Library, medRxiv and ClinicalTrials.gov from inception to January 2023 for all eligible studies. We conducted our meta-analysis using RevMan 5.4 with risk ratio (RR) and mean difference (MD) as the effect measures.

Eligibility criteria: All RCTs that investigated the efficacy of colchicine treatment in COVID-19 patients as compared with placebo or standard of care were included. Studies that used colchicine prophylactically were excluded.

Results: A total of 23 RCTs were included in this review. Colchicine did not decrease the risk of mortality (RR 0.99; 95% CI: 0.93-1.05; I^2=0%) with the results being consistent among both hospitalized and non-hospitalized patients. There were no significant differences between the colchicine and control groups in the incidence of mechanical ventilation, risk of ICU admission, risk of hospital admission, length of hospital stay, and the rate of no recovery.

Conclusions: The results of this meta-analysis do not support the use of colchicine as a treatment for reducing the risk of mortality or improving other clinical outcomes in patients with COVID-19. However, RCTs investigating early treatment with colchicine (within 5 days of symptom onset) are needed fully elucidate the potential benefits of colchicine in this patient population.

PROSPERO registration number: CRD42022369850

Keywords: colchicine; COVID-19; SARS-CoV-2; meta-analysis

For peer review only

Strengths and limitations of this study

- This systematic review used a comprehensive search for articles evaluating colchicine treatment for COVID-19 patients.
- We performed the largest meta-analysis to date by pooling results from 23 trials.
- There was significant interstudy heterogeneity in the overall analysis of some outcomes, such as length of hospitalization.
- Our results may not be generalizable to the usage of colchicine early in the course of the disease as most RCTs are of late-stage treatment.

For peer review only

1 INTRODUCTION

Unprecedented global research has been conducted as a result of the COVID-19 pandemic. Clinical trials conducted in response to COVID-19 have enabled us to administer the drug, monitor the results, and develop an appropriate conclusion. Even though particular antiviral medications and monoclonal antibodies have been approved to treat COVID-19,[1–3] ongoing clinical trials are being conducted to combat the virus more successfully, particularly using repurposed drugs which provide a cheap therapeutic alternative.[4,5]

Colchicine, an anti-inflammatory medication commonly used to treat gout, recurrent pericarditis, and familial Mediterranean fever, is being extensively investigated among COVID-19 patients.[6] Inhibiting neutrophil chemotaxis, suppressing inflammasome signaling and preventing the cytokine storm are some of the potential benefits of colchicine.[6] Although colchicine has been the subject of multiple clinical trials in COVID-19 patients, findings from smaller randomized controlled trials (RCTs) that were positive were later contradicted by results from major RCTs.[6] Therefore, we performed this meta-analysis to evaluate the safety and efficacy of colchicine for the treatment of COVID-19 patients and to interpret the significance of the latest data by incorporating it into our review.

For peer review only

2 METHODS

Our meta-analysis was registered with PROSPERO (CRD42022369850) and conducted by following the recommendations of Preferred Reporting Items for Systematic Reviews and Meta-Analyses (PRISMA).[7] The PRISMA checklist is provided in Supplementary Table 1.

2.1 Search strategy

We searched MEDLINE (via PubMed), Embase, the Cochrane Library, medRxiv and ClinicalTrials.gov from inception to January 2023 using a search strategy consisting of terms related to "colchicine" and "COVID-19" without any restrictions. Grey literature and the reference lists of the relevant studies were also explored to include all eligible trials. The detailed search strategy for different databases is given in Supplementary Table 2.

2.2 Study selection and eligibility criteria

All the literature obtained from our searches was imported into Mendeley Desktop 1.19.8 and duplicates were removed. The remaining articles were subjected to a rigorous screening process by two independent reviewers. The inclusion criteria were RCTs that investigated the efficacy of colchicine treatment in COVID-19 patients as compared with placebo or standard of care. Studies that used colchicine prophylactically were excluded.

2.3 Data extraction and outcomes

We extracted all information relating to the study characteristics such as author names, location, study population, details of intervention and comparator groups, and our outcomes of interest. We chose all-cause mortality as our primary outcome while the secondary outcomes included the incidence of mechanical ventilation (MV), risk of intensive care unit (ICU) admission, risk of hospitalization, the length of hospital stay, and the rate of no recovery (proportion of patients with no symptomatic resolution at the end of study).

2.4 Quality assessment

The risk of bias was assessed using the revised Cochrane Risk of Bias tool (RoB 2.0).[8] A rating of low risk of bias, some concerns or a high risk of bias was assigned to each study using

For peer review only

five domains: randomization process, deviations from intended interventions, missing outcome data, measurement of the outcome, and selection of reported result.

2.5 Data analysis

We conducted our meta-analysis using RevMan 5.4 with risk ratio (RR) and mean difference (MD) as the effect measures for categorical and continuous variables, respectively. We utilized a random-effects model as we anticipated our included studies to be substantially heterogeneous. We evaluated heterogeneity using the Chi^2 test and the I^2 statistic. We used the Cochrane Handbook for Systematic Reviews of Intervention to interpret the values of the I^2 statistic.[9] We conducted a subgroup analysis for our primary outcome on the basis of the study population (hospitalized vs. non-hospitalized patients). In addition, we conducted a sensitivity analysis on our primary outcome by excluding trials that used colchicine in combination with another intervention. For outcomes with data from 10 studies or more, we assessed publication bias by constructing funnel plots and running Egger's test for funnel plot asymmetry.

3 RESULTS

3.1 Search results and study characteristics

A total of 23 RCTs were eligible for inclusion in our meta-analysis.[10,11,20–29,12,30–32,13–19] The details of the screening process are presented in Figure 1.

Seventeen RCTs were conducted in hospitalized patients, four evaluated outpatients only,[20,24,25,32] and one RCT included both hospitalized and non-hospitalized patients.[29] Most of the trials had small sample sizes while the RECOVERY trial was the largest RCT with 11,340 patients.[26] The trials employed a variety of colchicine regimens and most were open-label that used standard of care as the comparator. Two RCTs combined colchicine with other

For peer review only - http://bmjopen.bmj.com/site/about/guidelines.xhtml

treatments; one with rosuvastatin,[28] and the other with herbal phenolic monoterpene.[15] The characteristics of each RCT are summarized in Supplementary Table 3.

3.2 Risk of bias assessment

According to RoB 2.0, seven studies were at a low risk of bias, one had a high risk of bias, and 12 had some concerns of bias (Figure 2). The most common source of bias was lack of blinding and deviations from protocol while some trials also had issues in the randomization process.

3.3 Results of the meta-analysis

3.3.1 Primary outcome

Our analysis, considering 25,824 patients from 20 RCTs, failed to find a statistically significant reduction in the risk of mortality with colchicine use (RR 0.99; 95% CI: 0.93-1.05; I^2=0%; Figure 2). The Egger's test indicated that publication bias was present (p = 0.002; Supplementary Figure 1). The results were consistent ($P_{interaction}$ = 0.59) among both hospitalized (RR 0.98; 95% CI: 0.90-1.06; I^2=5%) and non-hospitalized patients (RR 0.82; 95% CI: 0.43-1.55; I^2=0%; Supplementary Figure 2). Sensitivity analysis by excluding two studies that used combination therapies in the intervention group,[15,28] produced results consistent with our main analysis (RR 1.00; 95% CI: 0.94-1.06; I^2=0%).

3.3.2 Secondary outcomes

There were no significant differences between the colchicine and control groups in the incidence of MV (RR 0.75; 95% CI: 0.48-1.18; I^2=40%; Supplementary Figure 3), risk of ICU admission (RR 0.77; 95% CI: 0.49-1.22; I^2=0%; Supplementary Figure 3), risk of hospital admission (RR 0.74; 95% CI: 0.48-1.16; I_2=70%; Supplementary Figure 4), length of hospital stay (MD -0.70 days; 95% CI: -2.30 to 0.89 days; I^2=90%; Supplementary Figure 5), and the rate of no recovery (RR 0.77; 95% CI: 0.56-1.05; I^2=77%; Supplementary Figure 6).

For peer review only

4 DISCUSSION

To the best of our knowledge, this is the largest meta-analysis to date to assess the effectiveness of colchicine for COVID-19. The results of this meta-analysis suggest that colchicine does not have a significant effect on the risk of mortality in patients with COVID-19. There was also no significant benefit of colchicine in reducing the risk of mechanical ventilation, ICU admission, hospital admission, or improving recovery rates.

Colchicine's regulation of the inflammatory response has been proposed as a means to inhibit the inflammation caused by COVID-19. The primary mechanism by which colchicine affects cells is through the inhibition of microtubule polymerization, which has a ripple effect on various cellular functions such as maintaining cell shape, signaling, cell division, migration, and transport.[6,33] Additionally, colchicine modulates the synthesis of inflammatory cytokines such as tumor necrosis factor (TNF), interleukin-1 (IL-1), and interleukin-6 (IL-6) and disrupts the activation of inflammasomes, neutrophil adhesion, and recruitment.[6,33] Given that COVID-19 is characterized by excessive inflammation and a cytokine response, colchicine's anti-inflammatory effects may have the potential to improve outcomes.

Our results are consistent with some of the previous meta-analyses which also demonstrated no significant clinical benefit of colchicine in COVID-19 patients.[34] On the other hand, the meta-analyses by Zein et al. and Kow et al. found that colchicine use was associated with a reduction in the risk of mortality and duration of hospital stay, respectively.[35,36] However, the meta-analysis by Zein et al. also incorporated observational studies, which increases the likelihood of confounding bias in their findings.[35] Additionally, all of the previous meta-analyses included a small number of RCTs with most including less than ten. Conversely, our meta-analysis pooled

For peer review only

results from 23 RCTs, therefore, including a significantly greater cumulative sample size and substantially increasing the confidence in our estimates.

It is worth noting that despite including a large number of RCTs, the results of our meta-analysis are still greatly influenced by the RECOVERY trial.[26] The RECOVERY trial is by far the largest trial of colchicine which found no clinical benefit of colchicine in COVID-19 patients. However, this was a very late-stage treatment RCT with the median time from symptom onset to treatment initiation being 9 days. It is well recognized that late initiation of therapy might result in decreased effectiveness of antivirals.[37] Therefore, early treatment (within 5 days of symptom onset) with colchicine might prove to be beneficial for COVID-19 patients; however, this needs to be investigated in future RCTs as all of the currently available RCTs evaluated late-stage treatment.

Some limitations of our meta-analysis should be noted. The quality of the RCTs included in the meta-analysis was mixed with 9 being of high quality, one being of low quality, and 13 having some concerns of bias. This may have affected the reliability of the results. Additionally, there was significant interstudy heterogeneity in the overall analysis of some outcomes, such as length of hospitalization. Our results may not be generalizable to the usage of colchicine early in the course of the disease as most RCTs are of late-stage treatment. Finally, there was evidence of the presence of publication bias in our meta-analysis implying that small studies that did not report positive findings may not have been published.

In conclusion, the results of this meta-analysis do not support the use of colchicine as a treatment for reducing the risk of mortality or improving other clinical outcomes in patients with COVID-

For peer review only

19. However, RCTs investigating early treatment with colchicine (within 5 days of symptom onset) are needed fully elucidate the potential benefits of colchicine in this patient population.

Statements and Declarations

Financial support

No financial support was received for this study.

Conflicts of interest

The authors report no relationships that could be construed as a conflict of interest.

Acknowledgements

Not applicable.

Availability of data

The data that support the findings of this study are available from the corresponding author upon reasonable request.

Ethics statements

Patient consent for publication

Not required.

Ethics approval

Not applicable.

Authors' contributions

For peer review only

Conception and design of study: H.A. Cheema, A. Shahid, K.Y. Lee, U. Malik; **acquisition of data:** M. Usman, M. A. Naeem, H.A. Cheema; **data analysis and/or interpretation:** S. Sahra, R. Sah, U. Malik, A. A. Hermis, W. Masood; **drafting or writing of the manuscript:** M. A. Naeem, U. Malik, S. Sahra, M. Usman, A. Shahid, W. Masood; **substantial revision or critical review of the manuscript:** H.A. Cheema, A.A. Hermis, R. Sah, K.Y. Lee.

All authors have approved the final version of the manuscript.

For peer review only

References

1 Agarwal A, Rochwerg B, Lamontagne F, *et al.* A living WHO guideline on drugs for covid-19. *BMJ* 2020;**370**:m3379. doi:10.1136/bmj.m3379

2 Fatima M, Azeem S, Saeed J, *et al.* Efficacy and safety of molnupiravir for COVID-19 patients. *Eur J Intern Med* 2022;**102**:118–21. doi:10.1016/j.ejim.2022.05.024

3 Cheema HA, Jafar U, Sohail A, *et al.* Nirmatrelvir–ritonavir for the treatment of COVID-19 patients: A systematic review and meta-analysis. *J Med Virol* 2023;**95**. doi:10.1002/jmv.28471

4 Cheema HA, Shafiee A, Athar MMT, *et al.* No evidence of clinical efficacy of famotidine for the treatment of COVID-19: a systematic review and meta-analysis. *J Infect* 2023;**86**:154–225. doi:10.1016/j.jinf.2022.11.022

5 Cheema HA, Jafar U, Elrashedy AA, *et al.* Efficacy and safety of fluvoxamine for the treatment of COVID-19 patients: A systematic review and meta-analysis. J. Infect. 2022;**85**:702–69. doi:10.1016/j.jinf.2022.10.012

6 Reyes AZ, Hu KA, Teperman J, *et al.* Anti-inflammatory therapy for COVID-19 infection: the case for colchicine. *Ann Rheum Dis* 2021;**80**:550 LP – 557. doi:10.1136/annrheumdis-2020-219174

7 Page MJ, McKenzie JE, Bossuyt PM, *et al.* The PRISMA 2020 statement: an updated guideline for reporting systematic reviews. *BMJ* 2021;**372**:n71. doi:10.1136/bmj.n71

8 Sterne JAC, Savović J, Page MJ, *et al.* RoB 2: a revised tool for assessing risk of bias in

For peer review only

randomised trials. *BMJ* 2019;**366**:l4898. doi:10.1136/bmj.l4898

9 Higgins JPT, Thomas J, Chandler J, *et al.*, editors. *Cochrane Handbook for Systematic Reviews of Interventions*. 2nd ed. Hoboken, New Jersey: : Wiley Blackwell 2019. doi:10.1002/9781119536604

10 Perricone C, Scarsi M, Brucato A, *et al.* Treatment with COLchicine in hospitalized patients affected by COVID-19: The COLVID-19 trial. *Eur J Intern Med* 2023;**107**:30–6. doi:10.1016/j.ejim.2022.10.016

11 Rahman M, Datta PK, Islam K, *et al.* Efficacy of colchicine in patients with moderate COVID-19: A double-blinded, randomized, placebo-controlled trial. *PLoS One* 2022;**17**:e0277790.https://doi.org/10.1371/journal.pone.0277790

12 Kasiri H, Ghazaiean M, Rouhani N, *et al.* The effects of colchicine on hospitalized COVID-19 patients: A randomized, double-blind, placebo-controlled clinical trial. *J Investig Med* 2023;:10815589221141816. doi:10.1177/10815589221141815

13 Sunil Naik K, Andhalkar N, Pendse S. Effect of colchicine and aspirin given together in patients with moderate COVID-19. *Contemp Clin Trials Commun* 2023;:101070. doi:10.1016/j.conctc.2023.101070

14 Pimenta Bonifácio L, Ramacciotti E, Agati LB, *et al.* Efficacy and Safety of Ixekizumab vs. Low-Dose IL-2 vs. Colchicine vs. Standard of Care on the Treatment of Patients Hospitalized with Moderate to Critical Covid-19: A Pilot Randomized Clinical Trial (STRUCK: Survival Trial Using Cyto kine Inhibitors). *SSRN Electron J* Published Online First: 3 May 2022. doi:10.2139/ssrn.4095747

For peer review only

15 Mostafaie A. Colchicine Plus Phenolic Monoterpenes to Treat COVID-19. ClinicalTrials.gov. https://clinicaltrials.gov/ct2/show/NCT04392141 (accessed 23 Jan 2023).

16 Salehzadeh F, Pourfarzi F, Ataei S. The Impact of Colchicine on COVID-19 patients: A Clinical Trial Study. *Mediterr J Rheumatol* 2022;**33**:232. doi:10.31138/mjr.33.2.232

17 Deftereos SG, Giannopoulos G, Vrachatis DA, *et al.* Effect of Colchicine vs Standard Care on Cardiac and Inflammatory Biomarkers and Clinical Outcomes in Patients Hospitalized with Coronavirus Disease 2019: The GRECCO-19 Randomized Clinical Trial. *JAMA Netw Open* 2020;**3**:1–14. doi:10.1001/jamanetworkopen.2020.13136

18 Cecconi A, Martinez-Vives P, Vera A, *et al.* Efficacy of short-course colchicine treatment in hospitalized patients with moderate to severe COVID-19 pneumonia and hyperinflammation: a randomized clinical trial. *Sci Rep* 2022;**12**:1–8. doi:10.1038/s41598-022-13424-6

19 Eikelboom JW, Jolly SS, Belley-Cote EP, *et al.* Colchicine and the combination of rivaroxaban and aspirin in patients hospitalised with COVID-19 (ACT): an open-label, factorial, randomised, controlled trial. *Lancet Respir Med* 2022;**19**:1–10. doi:10.1016/S2213-2600(22)00298-3

20 Pourdowlat G, Saghafi F, Mozafari A, *et al.* Efficacy and safety of colchicine treatment in patients with COVID-19: A prospective, multicenter, randomized clinical trial. *Phyther Res* 2022;**36**:891–8. doi:10.1002/ptr.7319

21 Absalón-Aguilar A, Rull-Gabayet M, Pérez-Fragoso A, *et al.* Colchicine Is Safe Though

For peer review only

Ineffective in the Treatment of Severe COVID-19: a Randomized Clinical Trial (COLCHIVID). *J Gen Intern Med* 2022;**37**:4–14. doi:10.1007/s11606-021-07203-8

22 Gorial FI, Maulood MF, Abdulamir AS, *et al.* Randomized controlled trial of colchicine add on to the standard therapy in moderate and severe corona virus Disease-19 infection. *Ann Med Surg* 2022;**77**:103593. doi:10.1016/j.amsu.2022.103593

23 Lopes MI, Bonjorno LP, Giannini MC, *et al.* Beneficial effects of colchicine for moderate to severe COVID-19: a randomised, double-blinded, placebo-controlled clinical trial. *RMD Open* 2021;**7**:e001455. doi:10.1136/rmdopen-2020-001455

24 Tardif JC, Bouabdallaoui N, L'Allier PL, *et al.* Colchicine for community-treated patients with COVID-19 (COLCORONA): a phase 3, randomised, double-blinded, adaptive, placebo-controlled, multicentre trial. *Lancet Respir Med* 2021;**9**:924–32. doi:10.1016/S2213-2600(21)00222-8

25 Dorward J, Yu LM, Hayward G, *et al.* Colchicine for COVID-19 in the community (PRINCIPLE): a randomised, controlled, adaptive platform trial. *Br J Gen Pract* 2022;**72**:E446–55. doi:10.3399/BJGP.2022.0083

26 Recovery Collaborative Group. Colchicine in patients admitted to hospital with COVID-19 (RECOVERY): a randomised, controlled, open-label, platform trial. *Lancet Respir Med* 2021;**9**:1419–26. doi:10.1016/S2213-2600(21)00435-5

27 Diaz R, Orlandini A, Castellana N, *et al.* Effect of Colchicine vs Usual Care Alone on Intubation and 28-Day Mortality in Patients Hospitalized with COVID-19: A Randomized Clinical Trial. *JAMA Netw Open* 2021;**4**:1–12. doi:10.1001/jamanetworkopen.2021.41328

For peer review only

28 Gaitán-Duarte HG, Álvarez-Moreno C, Rincón-Rodríguez CJ, *et al.* Effectiveness of rosuvastatin plus colchicine, emtricitabine/tenofovir and combinations thereof in hospitalized patients with COVID-19: a pragmatic, open-label randomized trial. *eClinicalMedicine* 2022;**43**:1–12. doi:10.1016/j.eclinm.2021.101242

29 Jalal A, Aref S, Albustany D. Effectiveness of colchicine among patients with COVID-19 infection: A randomized, open-labeled, clinical trial. *Indian J Rheumatol* 2022;**17**:136. doi:10.4103/injr.injr_264_21

30 Pascual-Figal DA, Roura-Piloto AE, Moral-Escudero E, *et al.* Colchicine in Recently Hospitalized Patients with COVID-19: A Randomized Controlled Trial (COL-COVID). *Int J Gen Med* 2021;**Volume 14**:5517–26. doi:10.2147/IJGM.S329810

31 Alsultan M, Obeid A, Alsamarrai O, *et al.* Efficacy of Colchicine and Budesonide in Improvement Outcomes of Patients with Coronavirus Infection 2019 in Damascus, Syria: A Randomized Control Trial. *Interdiscip Perspect Infect Dis* 2021;**2021**:1–7. doi:10.1155/2021/2129006

32 Eikelboom JW, Jolly SS, Belley-cote EP, *et al.* Articles Colchicine and aspirin in community patients with COVID-19 (ACT): an open-label , factorial , randomised , controlled trial. *Lancet Respir* 2022;**19**:1–9. doi:10.1016/S2213-2600(22)00299-5

33 Ghaith HS, Gabra MD, Nafady MH, *et al.* A Review of the Rational and Current Evidence on Colchicine for COVID-19. *Curr Pharm Des* 2022;**28**:3194–201. doi:10.2174/1381612827666211210142352

34 Lan S-H, Hsu C-K, Lai C-C, *et al.* Effect of colchicine on the outcomes of patients with

For peer review only

COVID-19: a systematic review and meta-analysis of randomised controlled trials. *Ann Med* 2022;**54**:1956–65. doi:10.1080/07853890.2022.2096919

35 Zein AFMZ, Raffaello WM. Effect of colchicine on mortality in patients with COVID-19 - A systematic review and meta-analysis. *Diabetes Metab Syndr* 2022;**16**:102395. doi:10.1016/j.dsx.2022.102395

36 Kow CS, Lee L-H, Ramachandram DS, *et al.* The effect of colchicine on mortality outcome and duration of hospital stay in patients with COVID-19: A meta-analysis of randomized trials. *Immunity, Inflamm Dis* 2022;**10**:255–64. doi:10.1002/iid3.562

37 Forrest JI, Rayner CR, Park JJH, *et al.* Early Treatment of COVID-19 Disease: A Missed Opportunity. *Infect Dis Ther* 2020;**9**:715–20. doi:10.1007/s40121-020-00349-8

For peer review only

Figure legends

Figure 1. PRISMA 2020 flowchart.

Figure 2. Quality assessment of included trials.

Figure 3. Effect of colchicine on all-cause mortality in COVID-19 patients.

For peer review only

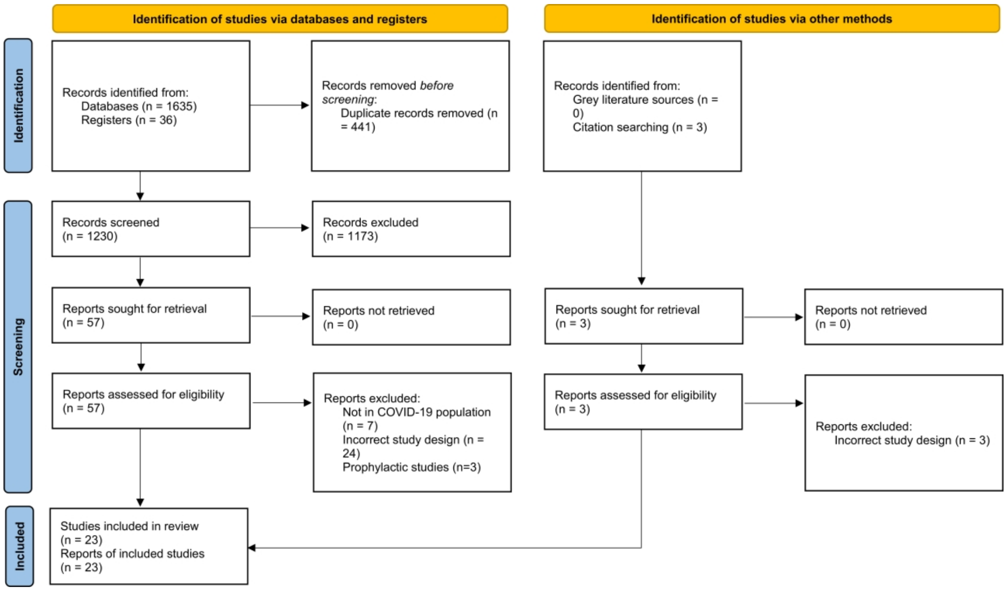

Figure 1. PRISMA 2020 flowchart.

1393x812mm (72 x 72 DPI)

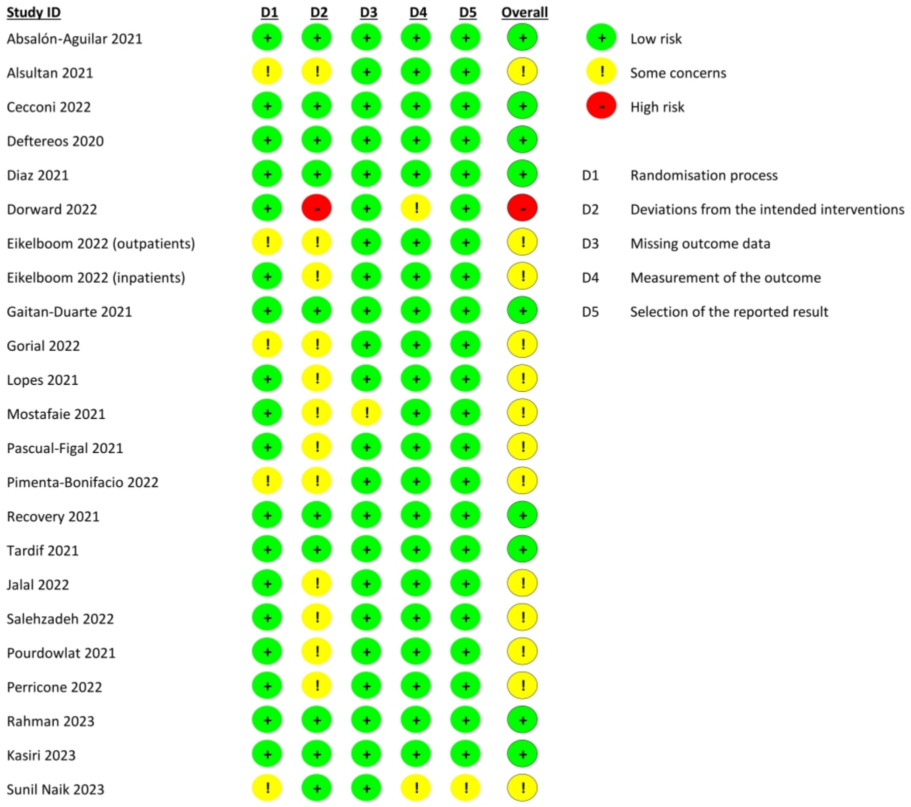

Figure 2. Quality assessment of included trials.

2089x1921mm (72 x 72 DPI)

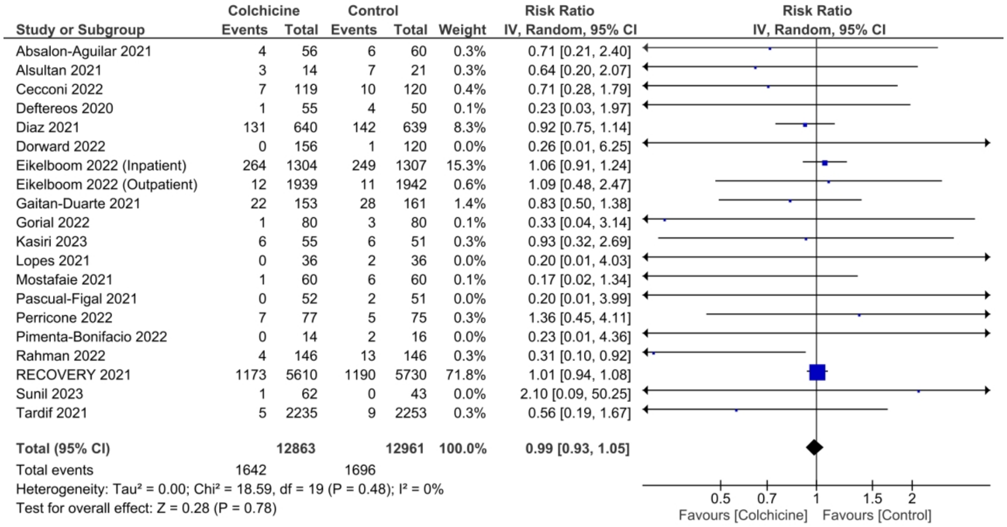

Figure 3. Effect of colchicine on all-cause mortality in COVID-19 patients.

928x493mm (72 x 72 DPI)

Supplementary Material

Colchicine for the treatment of COVID-19 patients: a rapid systematic review and meta-analysis of randomized controlled trials

This supplemental material has been provided by the authors to give readers additional information about their work.

For peer review only

Table S1. PRISMA checklist.

Section and Topic	Item #	Checklist item	Reported on page #
**TITLE**			
Title	1	Identify the report as a systematic review.	1
**ABSTRACT**			
Abstract	2	See the PRISMA 2020 for Abstracts checklist.	3
**INTRODUCTION**			
Rationale	3	Describe the rationale for the review in the context of existing knowledge.	4
Objectives	4	Provide an explicit statement of the objective(s) or question(s) the review addresses.	4
**METHODS**			
Eligibility criteria	5	Specify the inclusion and exclusion criteria for the review and how studies were grouped for the syntheses.	5
Information sources	6	Specify all databases, registers, websites, organisations, reference lists and other sources searched or consulted to identify studies. Specify the date when each source was last searched or consulted.	5
Search strategy	7	Present the full search strategies for all databases, registers and websites, including any filters and limits used.	Suppl.
Selection process	8	Specify the methods used to decide whether a study met the inclusion criteria of the review, including how many reviewers screened each record and each report retrieved, whether they worked independently, and if applicable, details of automation tools used in the process.	5
Data collection process	9	Specify the methods used to collect data from reports, including how many reviewers collected data from each report, whether they worked independently, any processes for obtaining or confirming data from study investigators, and if applicable, details of automation tools used in the process.	5
Data items	10a	List and define all outcomes for which data were sought. Specify whether all results that were compatible with each outcome domain in each study were sought (e.g. for all measures, time points, analyses), and if not, the methods used to decide which results to collect.	5
	10b	List and define all other variables for which data were sought (e.g. participant and intervention characteristics, funding sources). Describe any assumptions made about any missing or unclear information.	5
Study risk of bias assessment	11	Specify the methods used to assess risk of bias in the included studies, including details of the tool(s) used, how many reviewers assessed each study and whether they worked independently, and if applicable, details of automation tools used in the process.	5,6
Effect measures	12	Specify for each outcome the effect measure(s) (e.g. risk ratio, mean difference) used in the synthesis or presentation of results.	6
Synthesis methods	13a	Describe the processes used to decide which studies were eligible for each synthesis (e.g. tabulating the study intervention characteristics and comparing against the planned groups for each synthesis (item #5)).	6

For peer review only - http://bmjopen.bmj.com/site/about/guidelines.xhtml

Section and Topic	Item #	Checklist item	Reported on page #
	13b	Describe any methods required to prepare the data for presentation or synthesis, such as handling of missing summary statistics, or data conversions.	6
	13c	Describe any methods used to tabulate or visually display results of individual studies and syntheses.	6
	13d	Describe any methods used to synthesize results and provide a rationale for the choice(s). If meta-analysis was performed, describe the model(s), method(s) to identify the presence and extent of statistical heterogeneity, and software package(s) used.	6
	13e	Describe any methods used to explore possible causes of heterogeneity among study results (e.g. subgroup analysis, meta-regression).	6
	13f	Describe any sensitivity analyses conducted to assess robustness of the synthesized results.	6
Reporting bias assessment	14	Describe any methods used to assess risk of bias due to missing results in a synthesis (arising from reporting biases).	6
Certainty assessment	15	Describe any methods used to assess certainty (or confidence) in the body of evidence for an outcome.	NA
**RESULTS**			
Study selection	16a	Describe the results of the search and selection process, from the number of records identified in the search to the number of studies included in the review, ideally using a flow diagram.	6, Figure 1
	16b	Cite studies that might appear to meet the inclusion criteria, but which were excluded, and explain why they were excluded.	Figure 1
Study characteristics	17	Cite each included study and present its characteristics.	6,7, Table 1
Risk of bias in studies	18	Present assessments of risk of bias for each included study.	7, Figure 2
Results of individual studies	19	For all outcomes, present, for each study: (a) summary statistics for each group (where appropriate) and (b) an effect estimate and its precision (e.g. confidence/credible interval), ideally using structured tables or plots.	Figures, suppl.
Results of syntheses	20a	For each synthesis, briefly summarise the characteristics and risk of bias among contributing studies.	7,8
	20b	Present results of all statistical syntheses conducted. If meta-analysis was done, present for each the summary estimate and its precision (e.g. confidence/credible interval) and measures of statistical heterogeneity. If comparing groups, describe the direction of the effect.	7,8
	20c	Present results of all investigations of possible causes of heterogeneity among study results.	7
	20d	Present results of all sensitivity analyses conducted to assess the robustness of the synthesized results.	7
Reporting	21	Present assessments of risk of bias due to missing results (arising from reporting biases) for each synthesis assessed.	7

For peer review only

Section and Topic	Item #	Checklist item	Reported on page #
biases			
Certainty of evidence	22	Present assessments of certainty (or confidence) in the body of evidence for each outcome assessed.	NA
**DISCUSSION**			
Discussion	23a	Provide a general interpretation of the results in the context of other evidence.	8-10
	23b	Discuss any limitations of the evidence included in the review.	9
	23c	Discuss any limitations of the review processes used.	9
	23d	Discuss implications of the results for practice, policy, and future research.	9,10
**OTHER INFORMATION**			
Registration and protocol	24a	Provide registration information for the review, including register name and registration number, or state that the review was not registered.	4
	24b	Indicate where the review protocol can be accessed, or state that a protocol was not prepared.	4
	24c	Describe and explain any amendments to information provided at registration or in the protocol.	NA
Support	25	Describe sources of financial or non-financial support for the review, and the role of the funders or sponsors in the review.	10
Competing interests	26	Declare any competing interests of review authors.	10
Availability of data, code and other materials	27	Report which of the following are publicly available and where they can be found: template data collection forms; data extracted from included studies; data used for all analyses; analytic code; any other materials used in the review.	10

For peer review only

Table S2. Search strategy for electronic databases.

**MEDLINE (PubMed)**	("colchicine"[MeSH Terms] OR "colchicine"[tiab]) AND ("COVID-19"[MeSH Terms] OR "COVID-19"[tiab] OR "SARS-CoV-2"[tiab] OR "coronavirus disease 2019"[tiab]) AND ("randomized controlled trial"[pt] OR "controlled clinical trial"[pt] OR "randomized"[tiab] OR "placebo"[tiab] OR "drug therapy"[Subheading] OR "therapeutics"[tiab] OR "treatment"[tiab] OR "intervention"[tiab] OR "clinical trial"[pt] OR "placebo-controlled trial"[tiab])
**Embase**	'colchicine'/exp OR 'colchicine':ab,ti AND ('COVID-19'/exp OR 'COVID-19':ab,ti OR 'SARS-CoV-2':ab,ti OR 'coronavirus disease 2019':ab,ti) AND ('clinical trial'/exp OR 'randomized controlled trial'/exp OR 'placebo'/exp OR 'double-blind procedure'/exp OR 'single-blind procedure'/exp OR 'clinical trial phase III'/exp OR 'clinical trial phase IV'/exp OR 'randomization'/exp OR 'placebo-controlled study'/exp OR 'crossover procedure'/exp OR 'factorial design'/exp OR 'control group'/exp)
**The Cochrane Library**	(("Colchicine"[Mesh]) OR ("colchicine"[tiab])) AND (("COVID-19"[Mesh]) OR ("SARS-CoV-2"[Mesh]) OR ("Coronavirus Disease 2019"[Mesh]) OR ("COVID-19"[tiab]) OR ("SARS-CoV-2"[tiab]) OR ("coronavirus disease 2019"[tiab]))

For peer review only

Table S3. Characteristics on included trials.

Sr No	Author, year	Country	Sample size	Age*	Sex, n (%)	Population	Intervention	Comparator
1	Absalón-Aguilar 2021	Mexico	116	53 (44–62)	Male: 76 (65.5)<br>Female: 40 (35.5)	Hospitalized COVID-19 adult patients with severe disease	1.5 mg of colchicine at the time of the recruitment in the study and 0.5 mg BID to complete 10 days of treatment	Placebo
2	Alsultan 2021	Syria	49 (35 were eligible as 14 received budesonide)	-	-	Hospitalized, moderate-to-severe ARDS, adult patients with either a positive PCR test of COVID-19 virus in specimens taken from the respiratory tracts or a negative PCR test but with clinical signs and symptoms of viral illness along with findings on chest CT compatible with COVID-19 infection	1.5 mg of colchicine followed by 0.5 mg after an hour on day 1, then 0.5 mg twice daily for the next 4 days	Supportive care only
3	Cecconi 2022	Spain	239	65.1 ± 16.0	Male: 141 (59)<br>Female: 98 (41)	Hospitalized moderate-to-severe COVID-19 patients	5 days of oral colchicine: 1 mg loading dose and then 0.5 mg/day	Placebo
4	Deftereos 2020	Greece	105	64 (54-76)	Male: 61 (58.1)<br>Female: 44 (41.9)	Hospitalized adult COVID-19 patients	The loading dose consisted of 1.5 mg of colchicine followed by 0.5 mg of colchicine 60 minutes later. The maintenance dosage was 0.5 mg colchicine twice daily until hospital discharge or a maximum of 21 days.	No colchicine
5	Diaz 2021	Argentina	1279	61.8 ± 14.6	Male: 830 (64.9)<br>Female: 449 (35.1)	Hospitalized severe COVID-19 patients	Colchicine was administered orally in a loading dose of 1.5 mg followed by 0.5 mg orally within 2 hours of the initial dose and 0.5 mg orally twice a day for 14 days or	Usual care with no colchicine

For peer review only - http://bmjopen.bmj.com/site/about/guidelines.xhtml

							discharge, whichever occurred first.	
6	Dorward 2022	UK	2293 (156 received colchicine)	-	-	Non-hospitalized COVID-19 patients	Colchicine 500 µg daily for 14 days	Usual care
7	Eikelboom 2022 (inpatients)	11 countries	2611	Colchicine: 56.1 ± 16.7 Control: 56.0 ± 16.0	Colchicine: Male: 762 (58.4), Female: 542 (31.6) Control: Male: 796 (60.9), Female: 511 (39.1)	Hospitalized COVID-19 patients	Colchicine 1.2 mg followed by 0.6 mg 2h later and then 0.6 mg twice daily in tablet form for 28 days	Usual care
8	Eikelboom 2022 (outpatients)	11 countries	3917	Colchicine: 45.0 ± 13.7 Control: 45.0 ± 13.3	Colchicine: Male: 1173 (60.5%) Female 766 (39.5%) Control: Male: 1177 (60·6%) Female 765 (39.4%)	Non-hospitalized COVID-19 patients	Oral colchicine 0.6 mg twice daily for 3 days and then 0.6 mg once daily for 25 days	Usual care
9	Gaitan-Duarte 2021	Bogota, Colombia	314	Colchicine + Rosuvastatin: 56.1 ± 13.2 Control: 55.3 ± 12.3	Colchicine: Male: 103 (68), Female: 50 (32) Standard care: Male 113 (70.2), Female 48 (29.8)	Hospitalized adult COVID-19 patients	Colchicine plus rosuvastatin (0.5 mg and 40 mg for 14 days)	Standard of care
10	Gorial 2022	Iraq	160	-	-	Hospitalized moderate-to-severe COVID-19 patients	Colchicine 1 mg daily orally for 7 days, then 0.5 mg daily for another 7 days	Standard treatment

For peer review only

11	Jalal 2022	Iraq	80	-	Colchicine: Male: 21 (52.5), Female: 19 (47.5) Control: Male: 19 (47.5), Female: 21 (52.5)	Patients with mild, moderate, or severe COVID-19 infection; either hospitalized or at home	Colchicine 0.5 mg twice daily for 14 days	Standard treatment
12	Kasiri 2023	Iran	106	54.62 ± 13.92	Male: 49 (46.2) Female: 57 (53.8)	Hospitalized adult severe COVID-19 patients	Colchicine 2 mg loading dose followed by 0.5 mg twice daily for 7 days	Placebo
13	Lopes 2021	Brazil	75	Colchicine group: 54.5 (42.5–64.5) Placebo: 55.0 (42.0–67.0)	Colchicine: Male 19 (53), Female: 17 (47) Placebo: Male: 14 (39), Female: 22 (61)	Hospitalized moderate-to-severe COVID-19 patients	Colchicine 0.5 mg thrice daily for 5 days, then 0.5mg twice daily for 5 days	Placebo
14	Mostafaie, unpublished	Iran	120	-	-	Hospitalized COVID-19 patients aged ≥ 10 years	Colchicine plus herbal phenolic monoterpene	Standard treatment
15	Pascual-Figal 2021	Spain	103	51 ± 12	Male: 54 (52.4) Female: 49 (47.6)	Hospitalized adult COVID-19 patients	Colchicine 1.5 mg loading dose, followed by 0.5 mg BID for one week and 0.5 mg per day for 28 days	Standard treatment
16	Perricone 2023	Italy	152	Colchicine: 69.1 ± 13.1 Control: 67.9 ± 15	Colchicine: Male: 47 (61), Female: 30 (39) Control: Male: 50 (67), Female: 25 (33)	Hospitalized adult COVID-19 patients	Colchicine 0.5 mg three times a day if weight was less than 100 kg or 1 mg twice a day if weight was more than 100 kg for a maximum of 30 days or until hospital discharge	Standard treatment
17	Pimenta Bonifácio 2022	Brazil	30	Colchicine: 56.22 ± 11.6 Control: 46.11 ± 10.6	Colchicine: Male: 10 (71), Female: 4 (29) Control: Male: 11 (69), Female 5 (31)	Hospitalized moderate-to-severe COVID-19 patients	Colchicine 0.5 mg every 8 hours for three days, followed by 0.5mg 2 complete four weeks of treatment	Standard of care

For peer review only

18	Pourdowlat 2021	Iran	165	54.72 ± 15.03	Male: 93 (63) Female: 72 (37)	Non-hospitalized moderate-to-severe COVID-19 patients	A 3-day course of 0.5 mg colchicine followed by a 12-day course of 1 mg colchicine	Standard of care
19	Rahman 2022	Bangladesh	299	47 (35–55)	Male: 190 (64.2) Female: 106 (35.8)	Hospitalized moderate COVID-19 patients	1.2 mg of colchicine on day 1 followed by daily treatment with 0.6 mg of colchicine for 13 days	Placebo
20	RECOVERY 2021	UK, Indonesia, Nepal	11340	63.4 ± 13·8	Colchicine: Male: 3897 (69), Female: 1713 (31) Control group: Male: 4012 (70), Female: 1718 (30)	Hospitalized moderate-to-severe adult COVID-19 patients	Colchicine 1 mg followed by 500 µg 12h later and then 500 µg twice a day by mouth or nasogastric tube for 10 days in total or until discharge	Standard of care
21	Salehzadeh 2022	Iran	100	Colchicine: 56.56 ± 17.16 Placebo: 55.56 ± 16.38	Male: 41 (41) Female 59 (59)	Hospitalized moderate COVID-19 patients	Colchicine 1 mg daily for six days	Placebo
22	Sunil Naik 2023	India	122	-	-	Hospitalized moderate COVID-19 patients	Colchicine 0.5 mg BID	Standard care
23	Tardif 2021	Brazil, Canada, Greece, South Africa, Spain, and the USA	4488	54·0 (47·0–61·0)	Colchicine: Males: 997 (44.6), Females: 1238 (55.4) Control: Males: 1070 (47.5), Female: 1183 (52.5)	Non-hospitalized COVID-19 patients aged ≥ 40 years	Colchicine 0·5 mg BID for the first 3 days and then once per day for 27 days	Placebo

*Data reported as mean ± SD and median (IQR)

BID, twice daily; ARDS, acute respiratory distress syndrome; PCR, polymerase chain reaction.

For peer review only

Supplementary Figure S1. Funnel plot for all-cause mortality.

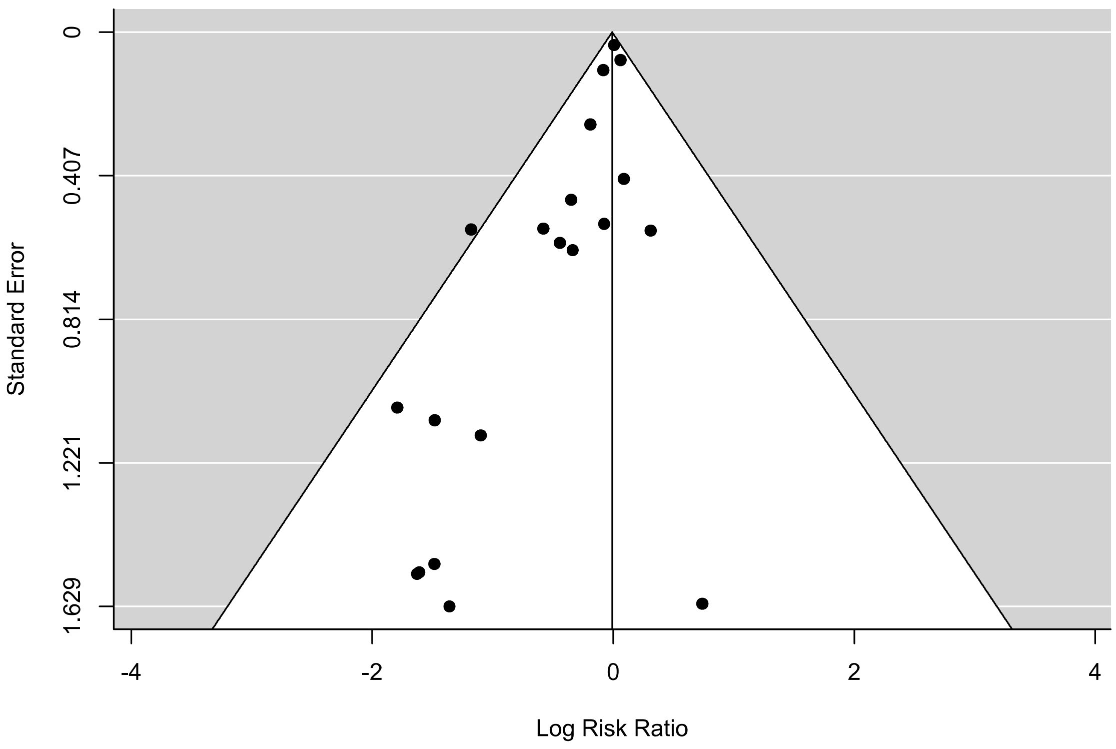

eview only

Supplementary Figure S2. Subgroup analysis of the effect of colchicine on the risk of mortality.

Study or Subgroup	Colchicine Events	Colchicine Total	Control Events	Control Total	Weight	Risk Ratio IV, Random, 95% CI
**1.6.1 Hospitalized**						
Absalon-Aguilar 2021	4	56	6	60	0.3%	0.71 [0.21, 2.40]
Alsultan 2021	3	14	7	21	0.3%	0.64 [0.20, 2.07]
Cecconi 2022	7	119	10	120	0.4%	0.71 [0.28, 1.79]
Deftereos 2020	1	55	4	50	0.1%	0.23 [0.03, 1.97]
Diaz 2021	131	640	142	639	8.3%	0.92 [0.75, 1.14]
Eikelboom 2022 (Inpatient)	264	1304	249	1307	15.3%	1.06 [0.91, 1.24]
Gaitan-Duarte 2021	22	153	28	161	1.4%	0.83 [0.50, 1.38]
Gorial 2022	1	80	3	80	0.1%	0.33 [0.04, 3.14]
Kasiri 2023	6	55	6	51	0.3%	0.93 [0.32, 2.69]
Lopes 2021	0	36	2	36	0.0%	0.20 [0.01, 4.03]
Mostafaie 2021	1	60	6	60	0.1%	0.17 [0.02, 1.34]
Pascual-Figal 2021	0	52	2	51	0.0%	0.20 [0.01, 3.99]
Perricone 2022	7	77	5	75	0.3%	1.36 [0.45, 4.11]
Pimenta-Bonifacio 2022	0	14	2	16	0.0%	0.23 [0.01, 4.36]
Rahman 2022	4	146	13	146	0.3%	0.31 [0.10, 0.92]
RECOVERY 2021	1173	5610	1190	5730	71.8%	1.01 [0.94, 1.08]
Sunil 2023	1	62	0	43	0.0%	2.10 [0.09, 50.25]
**Subtotal (95% CI)**		**8533**		**8646**	**99.1%**	**0.98 [0.90, 1.06]**
Total events	1625		1675			
Heterogeneity: Tau² = 0.00; Chi² = 16.79, df = 16 (P = 0.40); I² = 5%						
Test for overall effect: Z = 0.47 (P = 0.64)						
**1.6.2 Non-hospitalized**						
Dorward 2022	0	156	1	120	0.0%	0.26 [0.01, 6.25]
Eikelboom 2022 (Outpatient)	12	1939	11	1942	0.6%	1.09 [0.48, 2.47]
Tardif 2021	5	2235	9	2253	0.3%	0.56 [0.19, 1.67]
**Subtotal (95% CI)**		**4330**		**4315**	**0.9%**	**0.82 [0.43, 1.55]**
Total events	17		21			
Heterogeneity: Tau² = 0.00; Chi² = 1.45, df = 2 (P = 0.48); I² = 0%						
Test for overall effect: Z = 0.61 (P = 0.54)						
**Total (95% CI)**		**12863**		**12961**	**100.0%**	**0.99 [0.93, 1.05]**
Total events	1642		1696			
Heterogeneity: Tau² = 0.00; Chi² = 18.59, df = 19 (P = 0.48); I² = 0%						
Test for overall effect: Z = 0.28 (P = 0.78)						
Test for subgroup differences: Chi² = 0.30, df = 1 (P = 0.59), I² = 0%						

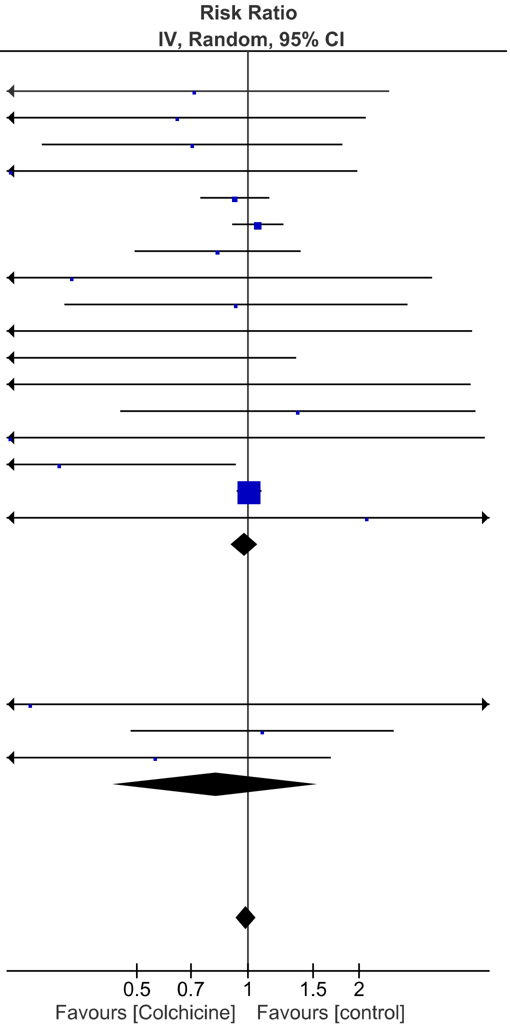

For peer review only

Supplementary Figure S3. Forest plot of the effect of colchicine on the risk of mechanical ventilation and ICU admission.

	Colchicine		Control			Risk Ratio
**Study or Subgroup**	**Events**	**Total**	**Events**	**Total**	**Weight**	**IV, Random, 95% CI**
**1.2.1 Risk of mechanical ventilation**						
Cecconi 2022	5	119	10	120	12.5%	0.50 [0.18, 1.43]
Deftereos 2020	1	55	5	50	4.0%	0.18 [0.02, 1.50]
Kasiri 2023	6	55	6	51	12.2%	0.93 [0.32, 2.69]
Pascual-Figal 2021	0	52	2	51	2.1%	0.20 [0.01, 3.99]
Perricone 2022	4	77	3	75	7.6%	1.30 [0.30, 5.61]
Rahman 2022	2	146	4	146	6.0%	0.50 [0.09, 2.69]
RECOVERY 2021	259	3815	228	3962	36.4%	1.18 [0.99, 1.40]
Tardif 2021	11	2235	21	2253	19.2%	0.53 [0.26, 1.09]
**Subtotal (95% CI)**		**6554**		**6708**	**100.0%**	**0.75 [0.48, 1.18]**
Total events	288		279			
Heterogeneity: Tau² = 0.14; Chi² = 11.69, df = 7 (P = 0.11); I² = 40%						
Test for overall effect: Z = 1.23 (P = 0.22)						
**1.2.2 ICU admission**						
Cecconi 2022	11	119	14	120	37.4%	0.79 [0.38, 1.67]
Kasiri 2023	12	55	9	51	34.7%	1.24 [0.57, 2.69]
Lopes 2021	2	36	4	36	7.8%	0.50 [0.10, 2.56]
Pascual-Figal 2021	2	52	4	51	7.7%	0.49 [0.09, 2.56]
Perricone 2022	1	77	4	75	4.4%	0.24 [0.03, 2.13]
Rahman 2022	2	146	5	146	7.9%	0.40 [0.08, 2.03]
**Subtotal (95% CI)**		**485**		**479**	**100.0%**	**0.77 [0.49, 1.22]**
Total events	30		40			
Heterogeneity: Tau² = 0.00; Chi² = 3.70, df = 5 (P = 0.59); I² = 0%						
Test for overall effect: Z = 1.10 (P = 0.27)						

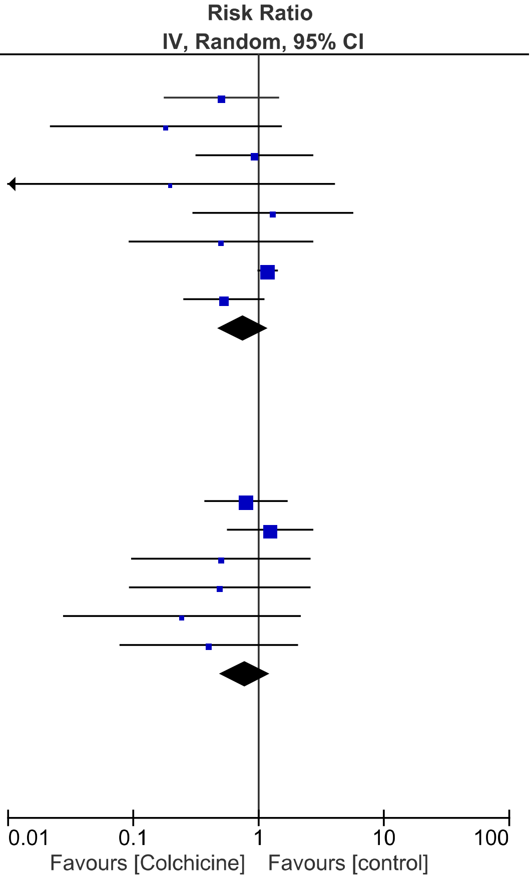

eview only

Supplementary Figure S4. Forest plot of the effect of colchicine on the risk of hospitalization.

	Colchicine		Control			Risk Ratio
**Study or Subgroup**	**Events**	**Total**	**Events**	**Total**	**Weight**	**IV, Random, 95% CI**
Eikelboom 2022 (Outpatient)	62	1939	61	1942	39.7%	1.02 [0.72, 1.44]
Pourdowlat 2021	5	102	18	100	15.5%	0.27 [0.11, 0.71]
Tardif 2021	101	2235	128	2253	44.8%	0.80 [0.62, 1.03]
**Total (95% CI)**		**4276**		**4295**	**100.0%**	**0.74 [0.48, 1.16]**
Total events	168		207			

Heterogeneity: Tau² = 0.10; Chi² = 6.69, df = 2 (P = 0.04); I² = 70%
Test for overall effect: Z = 1.30 (P = 0.19)

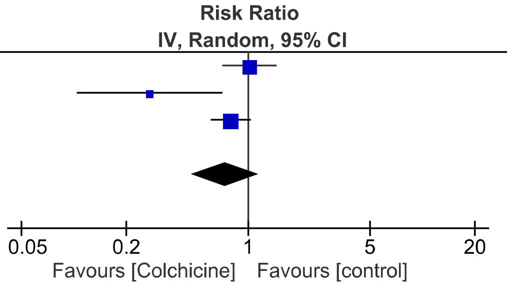

For peer review only

Supplementary Figure S5. Forest plot of the effect of colchicine on the length of hospital stay.

	Colchicine			Control				Mean Difference
Study or Subgroup	Mean	SD	Total	Mean	SD	Total	Weight	IV, Random, 95% CI
Absalon-Aguilar 2021	7.916	4.26	56	8.33	4.07	60	13.2%	-0.41 [-1.93, 1.10]
Jalal 2022	18.4	5.81	40	24.24	8.63	40	9.3%	-5.84 [-9.06, -2.62]
Lopes 2021	7	2.96	36	9.33	3.7	36	13.1%	-2.33 [-3.88, -0.78]
Mostafaie 2021	6.39	2.59	54	4.17	1.34	59	14.4%	2.22 [1.45, 2.99]
Pascual-Figal 2021	6.6	3.86	52	5.76	4.89	51	12.8%	0.84 [-0.86, 2.54]
Perricone 2022	14.1	10.4	77	14.7	8.1	75	9.9%	-0.60 [-3.56, 2.36]
Rahman 2022	10.67	5.93	146	10	6.67	146	13.3%	0.67 [-0.78, 2.12]
Salehzadeh 2022	6.28	2.51	50	8.12	2.66	50	14.1%	-1.84 [-2.85, -0.83]
**Total (95% CI)**			**511**			**517**	**100.0%**	**-0.70 [-2.30, 0.89]**

Heterogeneity: Tau² = 4.44; Chi² = 67.53, df = 7 (P < 0.00001); I² = 90%

Test for overall effect: Z = 0.86 (P = 0.39)

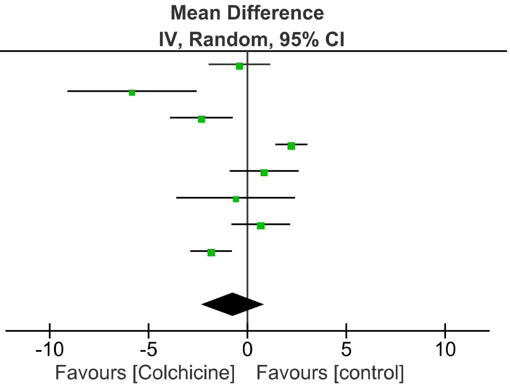

For peer review only

Supplementary Figure S6. Forest plot of the effect of colchicine on the rate of no recovery.

	Colchicine		Control			Risk Ratio
**Study or Subgroup**	**Events**	**Total**	**Events**	**Total**	**Weight**	**IV, Random, 95% CI**
Dorward 2022	42	156	306	1145	25.3%	1.01 [0.76, 1.33]
Gorial 2022	12	80	32	80	14.8%	0.38 [0.21, 0.67]
Kasiri 2023	7	55	9	51	8.5%	0.72 [0.29, 1.79]
Pourdowlat 2021	25	102	40	100	20.1%	0.61 [0.40, 0.93]
RECOVERY 2021	1709	5610	1698	5730	31.3%	1.03 [0.97, 1.09]
**Total (95% CI)**		**6003**		**7106**	**100.0%**	**0.77 [0.56, 1.05]**
Total events	1795		2085			

Heterogeneity: Tau² = 0.08; Chi² = 17.39, df = 4 (P = 0.002); I² = 77%

Test for overall effect: Z = 1.65 (P = 0.10)

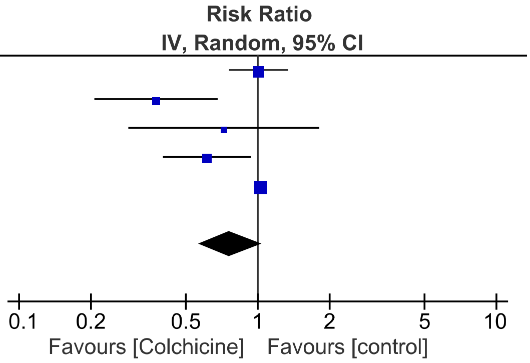

For peer review only

PRISMA 2020 checklist

Section and Topic	Item #	Checklist item	Reported on page #
**TITLE**			
Title	1	Identify the report as a systematic review.	1
**ABSTRACT**			
Abstract	2	See the PRISMA 2020 for Abstracts checklist.	3
**INTRODUCTION**			
Rationale	3	Describe the rationale for the review in the context of existing knowledge.	4
Objectives	4	Provide an explicit statement of the objective(s) or question(s) the review addresses.	4
**METHODS**			
Eligibility criteria	5	Specify the inclusion and exclusion criteria for the review and how studies were grouped for the syntheses.	5
Information sources	6	Specify all databases, registers, websites, organisations, reference lists and other sources searched or consulted to identify studies. Specify the date when each source was last searched or consulted.	5
Search strategy	7	Present the full search strategies for all databases, registers and websites, including any filters and limits used.	Suppl.
Selection process	8	Specify the methods used to decide whether a study met the inclusion criteria of the review, including how many reviewers screened each record and each report retrieved, whether they worked independently, and if applicable, details of automation tools used in the process.	5
Data collection process	9	Specify the methods used to collect data from reports, including how many reviewers collected data from each report, whether they worked independently, any processes for obtaining or confirming data from study investigators, and if applicable, details of automation tools used in the process.	5
Data items	10a	List and define all outcomes for which data were sought. Specify whether all results that were compatible with each outcome domain in each study were sought (e.g. for all measures, time points, analyses), and if not, the methods used to decide which results to collect.	5
	10b	List and define all other variables for which data were sought (e.g. participant and intervention characteristics, funding sources). Describe any assumptions made about any missing or unclear information.	5
Study risk of bias assessment	11	Specify the methods used to assess risk of bias in the included studies, including details of the tool(s) used, how many reviewers assessed each study and whether they worked independently, and if applicable, details of automation tools used in the process.	5,6
Effect measures	12	Specify for each outcome the effect measure(s) (e.g. risk ratio, mean difference) used in the synthesis or presentation of results.	6
Synthesis methods	13a	Describe the processes used to decide which studies were eligible for each synthesis (e.g. tabulating the study intervention characteristics and comparing against the planned groups for each synthesis (item #5)).	6

For peer review only - http://bmjopen.bmj.com/site/about/guidelines.xhtml

Section and Topic	Item #	Checklist item	Reported on page #
	13b	Describe any methods required to prepare the data for presentation or synthesis, such as handling of missing summary statistics, or data conversions.	6
	13c	Describe any methods used to tabulate or visually display results of individual studies and syntheses.	6
	13d	Describe any methods used to synthesize results and provide a rationale for the choice(s). If meta-analysis was performed, describe the model(s), method(s) to identify the presence and extent of statistical heterogeneity, and software package(s) used.	6
	13e	Describe any methods used to explore possible causes of heterogeneity among study results (e.g. subgroup analysis, meta-regression).	6
	13f	Describe any sensitivity analyses conducted to assess robustness of the synthesized results.	6
Reporting bias assessment	14	Describe any methods used to assess risk of bias due to missing results in a synthesis (arising from reporting biases).	6
Certainty assessment	15	Describe any methods used to assess certainty (or confidence) in the body of evidence for an outcome.	NA
**RESULTS**			
Study selection	16a	Describe the results of the search and selection process, from the number of records identified in the search to the number of studies included in the review, ideally using a flow diagram.	6, Figure 1
	16b	Cite studies that might appear to meet the inclusion criteria, but which were excluded, and explain why they were excluded.	Figure 1
Study characteristics	17	Cite each included study and present its characteristics.	6,7, Table 1
Risk of bias in studies	18	Present assessments of risk of bias for each included study.	7, Figure 2
Results of individual studies	19	For all outcomes, present, for each study: (a) summary statistics for each group (where appropriate) and (b) an effect estimate and its precision (e.g. confidence/credible interval), ideally using structured tables or plots.	Figures, suppl.
Results of syntheses	20a	For each synthesis, briefly summarise the characteristics and risk of bias among contributing studies.	7,8
	20b	Present results of all statistical syntheses conducted. If meta-analysis was done, present for each the summary estimate and its precision (e.g. confidence/credible interval) and measures of statistical heterogeneity. If comparing groups, describe the direction of the effect.	7,8
	20c	Present results of all investigations of possible causes of heterogeneity among study results.	7
	20d	Present results of all sensitivity analyses conducted to assess the robustness of the synthesized results.	7
Reporting	21	Present assessments of risk of bias due to missing results (arising from reporting biases) for each synthesis assessed.	7

For peer review only

Section and Topic	Item #	Checklist item	Reported on page #
biases			
Certainty of evidence	22	Present assessments of certainty (or confidence) in the body of evidence for each outcome assessed.	NA
**DISCUSSION**			
Discussion	23a	Provide a general interpretation of the results in the context of other evidence.	8-10
	23b	Discuss any limitations of the evidence included in the review.	9
	23c	Discuss any limitations of the review processes used.	9
	23d	Discuss implications of the results for practice, policy, and future research.	9,10
**OTHER INFORMATION**			
Registration and protocol	24a	Provide registration information for the review, including register name and registration number, or state that the review was not registered.	4
	24b	Indicate where the review protocol can be accessed, or state that a protocol was not prepared.	4
	24c	Describe and explain any amendments to information provided at registration or in the protocol.	NA
Support	25	Describe sources of financial or non-financial support for the review, and the role of the funders or sponsors in the review.	10
Competing interests	26	Declare any competing interests of review authors.	10
Availability of data, code and other materials	27	Report which of the following are publicly available and where they can be found: template data collection forms; data extracted from included studies; data used for all analyses; analytic code; any other materials used in the review.	10

For peer review only

BMJ Open

Colchicine for the treatment of COVID-19 patients: an updated systematic review and meta-analysis of randomized controlled trials

Journal:	*BMJ Open*
Manuscript ID	bmjopen-2023-074373.R1
Article Type:	Original research
Date Submitted by the Author:	12-Feb-2024
Complete List of Authors:	Cheema, Huzaifa Ahmad ; King Edward Medical University, Department of Medicine<br>Jafar, Uzair; King Edward Medical University, Department of Medicine<br>Usman, Muhammad; King Edward Medical University, Department of Medicine<br>Masood, Waniyah; Dow University of Health Sciences, Department of Medicine<br>Naseem, Muhammad Arsal; King Edward Medical University, Department of Medicine<br>Shahid, Abia; King Edward Medical University, Department of Medicine<br>Hermis, Alaa Hamza; Al-Mustaqbal University College<br>Sahra, Syeda; The University of Oklahoma Health Sciences Center, Department of Infectious Diseases<br>Sah, Ranjit; Tribhuvan University Teaching Hospital; Harvard Medical School<br>Lee, Ka Yiu; Mid Sweden University, Department of Health Sciences
**Primary Subject Heading**:	Infectious diseases
Secondary Subject Heading:	Immunology (including allergy)
Keywords:	COVID-19, VIROLOGY, CLINICAL PHARMACOLOGY

SCHOLARONE™
Manuscripts

I, the Submitting Author has the right to grant and does grant on behalf of all authors of the Work (as defined in the below author licence), an exclusive licence and/or a non-exclusive licence for contributions from authors who are: i) UK Crown employees; ii) where BMJ has agreed a CC-BY licence shall apply, and/or iii) in accordance with the terms applicable for US Federal Government officers or employees acting as part of their official duties; on a worldwide, perpetual, irrevocable, royalty-free basis to BMJ Publishing Group Ltd ("BMJ") its licensees and where the relevant Journal is co-owned by BMJ to the co-owners of the Journal, to publish the Work in this journal and any other BMJ products and to exploit all rights, as set out in our licence.

The Submitting Author accepts and understands that any supply made under these terms is made by BMJ to the Submitting Author unless you are acting as an employee on behalf of your employer or a postgraduate student of an affiliated institution which is paying any applicable article publishing charge ("APC") for Open Access articles. Where the Submitting Author wishes to make the Work available on an Open Access basis (and intends to pay the relevant APC), the terms of reuse of such Open Access shall be governed by a Creative Commons licence – details of these licences and which Creative Commons licence will apply to this Work are set out in our licence referred to above.

Other than as permitted in any relevant BMJ Author's Self Archiving Policies, I confirm this Work has not been accepted for publication elsewhere, is not being considered for publication elsewhere and does not duplicate material already published. I confirm all authors consent to publication of this Work and authorise the granting of this licence.

For peer review only

Colchicine for the treatment of COVID-19 patients: an updated systematic review and meta-analysis of randomized controlled trials

Running title: Colchicine for COVID-19 patients

Huzaifa Ahmad Cheema*[1]; Uzair Jafar*[1]; Muhammad Usman[1]; Waniyah Masood[2]; Muhammad Arsal Naseem[1]; Abia Shahid[1]; Alaa Hamza Hermis[3]; Syeda Sahra[4]; Ranjit Sah[5,6]; Ka Yiu Lee[7]

*These authors contributed equally and are co-first authors.

1. Division of Infectious Diseases, Department of Medicine, King Edward Medical University, Lahore, Pakistan.
2. Department of Medicine, Dow University of Health Sciences, Karachi, Pakistan.
3. Al-Mustaqbal University College, 51001 Hillah, Babylon, Iraq.
4. Department of Infectious Diseases, University of Oklahoma Health Sciences Center, Oklahoma City, OK, United States.
5. Tribhuvan University Teaching Hospital, Institute of Medicine, Kathmandu, Nepal.
6. Harvard Medical School, Boston, MA, United States.
7. Swedish Winter Sports Research Centre, Department of Health Sciences, Mid Sweden University, Östersund, Sweden.

Corresponding authors:

1) Ka Yiu Lee, PhD; kyle.lee@miun.se

Address: Swedish Winter Sports Research Centre, Department of Health Sciences, Mid Sweden University, Östersund, Sweden

For peer review only

Telephone: +46725816133

2) Huzaifa Ahmad Cheema, MBBS: huzaifacheema@kemu.edu.pk;

Address: Division of Infectious Diseases, Department of Medicine, King Edward Medical University, Nila Gumbad Chowk, Neela Gumbad Lahore, Punjab 54000

Telephone: +923324487886

Word count: 1899

For peer review only

Abstract

Objectives: We conducted an updated meta-analysis to investigate the effect of colchicine treatment on clinical outcomes in COVID-19 patients.

Design: Systematic review and meta-analysis.

Data sources: We searched PubMed, Embase, the Cochrane Library, medRxiv, and ClinicalTrials.gov from inception to January 2023.

Eligibility criteria: All randomized controlled trials (RCTs) that investigated the efficacy of colchicine treatment in COVID-19 patients as compared with placebo or standard of care were included. There were no language restrictions. Studies that used colchicine prophylactically were excluded.

Data extraction and synthesis: We extracted all information relating to the study characteristics, such as author names, location, study population, details of intervention and comparator groups, and our outcomes of interest. We conducted our meta-analysis using RevMan 5.4 with risk ratio (RR) and mean difference (MD) as the effect measures.

Results: We included 23 RCTs (28249 participants) in this review. Colchicine did not decrease the risk of mortality (RR 0.99; 95% CI: 0.93-1.05; I^2=0%; 20 RCTs, 25824 participants), with the results being consistent among both hospitalized and non-hospitalized patients. There were no significant differences between the colchicine and control groups in other relevant clinical outcomes including the incidence of mechanical ventilation (RR 0.75; 95% CI: 0.48-1.18; p=0.22; I^2=40%; 8 RCTs, 13262 participants), ICU admission (RR 0.77; 95% CI: 0.49-1.22; p=0.27; I^2=0%; 6 RCTs, 961 participants), and hospital admission (RR 0.74; 95% CI: 0.48-1.16; p=0.19; I_2=70%; 3 RCTs, 8572 participants).

For peer review only

Conclusions: The results of this meta-analysis do not support the use of colchicine as a treatment for reducing the risk of mortality or improving other relevant clinical outcomes in patients with COVID-19. However, RCTs investigating early treatment with colchicine (within 5 days of symptom onset or in patients with early-stage disease) are needed to fully elucidate the potential benefits of colchicine in this patient population.

PROSPERO registration number: CRD42022369850

Keywords: colchicine; COVID-19; SARS-CoV-2; meta-analysis

For peer review only

Strengths and limitations of this study

- This systematic review used a comprehensive search for articles evaluating colchicine treatment for COVID-19 patients.
- We performed the largest meta-analysis to date by pooling results from 23 trials.
- There was significant interstudy heterogeneity in the overall analysis of some outcomes, such as length of hospitalization.
- Our results may not be generalizable to the usage of colchicine early in the course of the disease as most RCTs are of late-stage treatment.

For peer review only

1 INTRODUCTION

Unprecedented global research has been conducted as a result of the COVID-19 pandemic. Even though particular antiviral medications, immunomodulators, and monoclonal antibodies have been approved to treat COVID-19,[1–3] ongoing clinical trials are being conducted to combat the virus more successfully, particularly using repurposed drugs and herbal medicines that provide a cheap therapeutic alternative.[4–6] Limited knowledge of COVID-19 molecular mechanisms and pathophysiology, lack of definitive supporting evidence, and expensive costs of some treatment options are some of the challenges faced in using different drugs for COVID-19.[7,8]

Colchicine, an anti-inflammatory medication commonly used to treat gout, recurrent pericarditis, and familial Mediterranean fever, is being extensively investigated among COVID-19 patients.[9] Inhibiting neutrophil chemotaxis, suppressing inflammasome signaling, and preventing the cytokine storm through microtubule depolarization are some of the potential benefits of colchicine in treating COVID-19.[9] Currently, guidelines from the World Health Organization (WHO) recommend against treatment with colchicine based on data from 10 studies.[1]

Although colchicine has been the subject of multiple clinical trials in COVID-19 patients, findings from smaller randomized controlled trials (RCTs) that were positive were later contradicted by results from major RCTs.[9] Therefore, we performed this meta-analysis to evaluate the safety and efficacy of colchicine for the treatment of COVID-19 patients and to interpret the significance of the latest data by incorporating it into our review.

For peer review only

2 METHODS

Our meta-analysis was registered with PROSPERO (CRD42022369850) and conducted by following the recommendations of Preferred Reporting Items for Systematic Reviews and Meta-Analyses (PRISMA).[10] The PRISMA checklist is provided in Supplementary Table 1.

2.1 Data sources and search strategy

We searched MEDLINE (via PubMed), Embase, the Cochrane Library, medRxiv, and ClinicalTrials.gov from inception to January 2023 using a search strategy consisting of terms related to "colchicine" and "COVID-19" without any restrictions. Grey literature and the reference lists of the relevant studies were also explored to include all eligible trials. We did not restrict our search based on language. The detailed search strategy for different databases is given in Supplementary Table 2.

2.2 Eligibility criteria

The inclusion criteria were: 1) population: patients with a diagnosis of COVID-19; 2) intervention: colchicine regardless of dosing or regimen; 3) comparator: placebo or standard of care; 4) outcome: reporting any outcome of interest; and 5) study design: RCTs only. Observational studies, quasi-randomized trials, and studies that used colchicine prophylactically were excluded.

2.3 Study selection

All the literature obtained from our searches was imported into Mendeley Desktop 1.19.8 and duplicates were removed. The remaining articles were subjected to a rigorous screening process by two independent reviewers in a two-stage process (title/abstract screening followed by full-text screening).

For peer review only

2.4 Data extraction and outcomes

We extracted all information relating to the study characteristics such as author names, location, study population, details of intervention and comparator groups, and our outcomes of interest. We chose all-cause mortality as our primary outcome while the secondary outcomes included the incidence of mechanical ventilation (MV), risk of intensive care unit (ICU) admission, risk of hospitalization, the length of hospital stay, and the rate of no recovery (proportion of patients with no symptomatic resolution at the end of study).

2.5 Quality assessment

The risk of bias was assessed using the revised Cochrane Risk of Bias tool (RoB 2.0).[11] A rating of low risk of bias, some concerns, or a high risk of bias was assigned to each study using five domains: randomization process, deviations from intended interventions, missing outcome data, measurement of the outcome, and selection of reported result.

2.6 Data analysis

We conducted our meta-analysis using RevMan 5.4 with risk ratio (RR) and mean difference (MD) as the effect measures for categorical and continuous variables, respectively. We utilized a random-effects model as we anticipated our included studies to be substantially heterogeneous. We evaluated heterogeneity using the Chi^2 test, the I^2 statistic, and the Tau^2 estimate. We used the Cochrane Handbook for Systematic Reviews of Intervention to interpret the values of the I^2 statistic.[12] We conducted a subgroup analysis for our primary outcome on the basis of the study population (hospitalized vs. non-hospitalized patients). In addition, we conducted a sensitivity analysis on our primary outcome by excluding trials that used colchicine in combination with another intervention. For outcomes with data from 10 studies or more, we

For peer review only - http://bmjopen.bmj.com/site/about/guidelines.xhtml

assessed publication bias by constructing funnel plots and running Egger's test for funnel plot asymmetry.

2.7 Patient and public involvement

None.

3 RESULTS

3.1 Search results and study characteristics

A total of 23 RCTs (28249 participants) were eligible for inclusion in our meta-analysis.[13–35] The details of the screening process are presented in Figure 1.

Seventeen RCTs were conducted in hospitalized patients, four evaluated outpatients only,[15,19,20,28] and one RCT included both hospitalized and non-hospitalized patients.[24] Most of the trials had small sample sizes while the RECOVERY trial was the largest RCT with 11,340 patients.[21] The trials employed a variety of colchicine regimens and most were open-label that used standard of care as the comparator. Two RCTs combined colchicine with other treatments; one with rosuvastatin,[23] and the other with herbal phenolic monoterpene.[31] The characteristics of each RCT are summarized in Supplementary Table 3.

3.2 Risk of bias assessment

According to RoB 2.0, seven studies were at a low risk of bias, one had a high risk of bias, and 12 had some concerns of bias (Figure 2). The most common source of bias was lack of blinding and deviations from protocol while some trials also had issues in the randomization process.

3.3 Results of the meta-analysis

3.3.1 Primary outcome

For peer review only

Our analysis, considering 25,824 patients from 20 RCTs, failed to find a statistically significant reduction in the risk of mortality with colchicine use (RR 0.99; 95% CI: 0.93-1.05; p=0.78; I^2=0%; Figure 3). The Egger's test indicated that publication bias was present (p = 0.002; Supplementary Figure 1). The results were consistent ($P_{interaction}$ = 0.59) among both hospitalized (RR 0.98; 95% CI: 0.90-1.06; p=0.64; I^2=5%) and non-hospitalized patients (RR 0.82; 95% CI: 0.43-1.55; p=0.54; I^2=0%; Supplementary Figure 2).

Sensitivity analysis by excluding two studies that used combination therapies in the intervention group,[23,31] produced results consistent with our main analysis (RR 1.00; 95% CI: 0.94-1.06; p=0.88; I^2=0%). The results remained congruous with our primary analysis after performing a second sensitivity analysis excluding studies with a high risk of bias or some concerns of bias in the domain of deviations from intended interventions (RR 0.97; 95% CI: 0.88-1.06; p=0.46; I^2=3%).

3.3.2 Secondary outcomes

There were no significant differences between the colchicine and control groups in the incidence of MV (RR 0.75; 95% CI: 0.48-1.18; p=0.22; I^2=40%; 8 RCTs, 13262 participants; Supplementary Figure 3), risk of ICU admission (RR 0.77; 95% CI: 0.49-1.22; p=0.27; I^2=0%; 6 RCTs, 961 participants; Supplementary Figure 3), risk of hospital admission (RR 0.74; 95% CI: 0.48-1.16; p=0.19; I_2=70%; 3 RCTs, 8572 participants; Supplementary Figure 4), length of hospital stay (MD -0.70 days; 95% CI: -2.30 to 0.89 days; p=0.39; I^2=90%; 8 RCTs, 1028 participants; Supplementary Figure 5), and the rate of no recovery (RR 0.77; 95% CI: 0.56-1.05; p=0.10; I^2=77%; 5 RCTs, 13109 participants; Supplementary Figure 6).

4 DISCUSSION

For peer review only

To the best of our knowledge, this is the most up-to-date meta-analysis to assess the effectiveness of colchicine for COVID-19. The results of this meta-analysis suggest that colchicine does not have a significant effect on the risk of mortality in patients with COVID-19. There was also no significant benefit of colchicine in reducing the risk of mechanical ventilation, ICU admission, hospital admission, or improving recovery rates.

Colchicine's regulation of the inflammatory response has been proposed as a means to inhibit the inflammation caused by COVID-19. The primary mechanism by which colchicine affects cells is through the inhibition of microtubule polymerization, which has a ripple effect on various cellular functions such as maintaining cell shape, signaling, cell division, migration, and transport.[9,36] Additionally, colchicine modulates the synthesis of inflammatory cytokines such as tumor necrosis factor (TNF), interleukin-1 (IL-1), and interleukin-6 (IL-6) and disrupts the activation of inflammasomes, neutrophil adhesion, and recruitment.[9,36] Given that COVID-19 is characterized by excessive inflammation and a cytokine response, colchicine's anti-inflammatory effects may have the potential to improve outcomes.

Our results are consistent with some of the previous meta-analyses which also demonstrated no significant clinical benefit of colchicine in COVID-19 patients.[37–41] On the other hand, the meta-analyses by Zein et al. and Golpour et al. [42,43] found that colchicine use was associated with a reduction in the risk of mortality, and Kow et al. demonstrated a shorter duration of hospital stay with colchicine.[44] However, the meta-analysis by Zein et al. and Golpour et al. also incorporated observational studies, which increases the likelihood of confounding bias in their findings.[42,43] Additionally, all of the previous meta-analyses included a small number of RCTs with most including less than or equal to eleven.[45] Conversely, our meta-analysis pooled

For peer review only

results from 23 RCTs, therefore, including a significantly greater cumulative sample size and substantially increasing the confidence in our estimates.

A recent umbrella review found that exposure to colchicine was associated with reduced mortality.[45] However, this discrepancy can be explained by the fact that it pooled estimates from meta-analyses instead of primary studies. Since there is a significant overlap in the included studies between the prior meta-analyses, pooling meta-analyzed effect sizes leads to erroneous and flawed results due to double-counting of outcome data. The correct approach would be to synthesize data from the primary studies to provide valid estimates.[46]

It is worth noting that despite including a large number of RCTs, the results of our meta-analysis are still greatly influenced by the RECOVERY trial.[21] The RECOVERY trial is by far the largest trial of colchicine which found no clinical benefit of colchicine in COVID-19 patients. However, this was a very late-stage treatment RCT with the median time from symptom onset to treatment initiation being 9 days. It is well recognized that late initiation of therapy might result in decreased effectiveness of antivirals.[47] Therefore, early treatment (within 5 days of symptom onset or at an early stage of the disease) with colchicine might prove to be beneficial for COVID-19 patients; however, this needs to be investigated in future RCTs as all of the currently available RCTs evaluated late-stage treatment.

Some limitations of our meta-analysis should be noted. The quality of the RCTs included in the meta-analysis was mixed with 9 being of high quality, one being of low quality, and 13 having some concerns of bias. This may have affected the reliability of the results. Additionally, there was significant interstudy heterogeneity in the overall analysis of some outcomes, such as length of hospitalization. Our results may not be generalizable to the usage of colchicine early in the

For peer review only

course of the disease as most RCTs are of late-stage treatment. Moreover, many studies either did not report the vaccination status of their participants or included unvaccinated patients only. Therefore, the COVID-19 vaccination status as a potential effect modifier needs to be studied further. Finally, there was evidence of the presence of publication bias in our meta-analysis implying that small studies that did not report positive findings may not have been published.

In conclusion, the results of this meta-analysis do not support the use of colchicine as a treatment for reducing the risk of mortality or improving other clinical outcomes in patients with COVID-19. However, RCTs investigating early treatment with colchicine (within 5 days of symptom onset or in patients with early-stage disease) are needed to fully elucidate the potential benefits of colchicine in this patient population.

Statements and Declarations

Funding statement

No financial support was received for this study.

Conflicts of interest

The authors report no relationships that could be construed as a conflict of interest.

Acknowledgements

Not applicable.

Availability of data

The data that support the findings of this study are available from the corresponding author upon reasonable request.

For peer review only

Ethics statements

Patient consent for publication

Not required.

Ethics approval

Not applicable.

Authors' contributions

Conception and design of study: H.A. Cheema, A. Shahid, K.Y. Lee, U. Jafar; **acquisition of data:** M. Usman, M. A. Naseem, H.A. Cheema; **data analysis and/or interpretation:** S. Sahra, R. Sah, U. Jafar, A. A. Hermis, W. Masood; **drafting or writing of the manuscript:** M. A. Naseem, U. Jafar, S. Sahra, M. Usman, A. Shahid, W. Masood; **substantial revision or critical review of the manuscript:** H.A. Cheema, A.A. Hermis, R. Sah, K.Y. Lee.

All authors have approved the final version of the manuscript.

For peer review only

References

1 Agarwal A, Rochwerg B, Lamontagne F, *et al.* A living WHO guideline on drugs for covid-19. *BMJ*. 2020;370:m3379.

2 Fatima M, Azeem S, Saeed J, *et al.* Efficacy and safety of molnupiravir for COVID-19 patients. *Eur J Intern Med*. 2022;102:118–21.

3 Cheema HA, Jafar U, Sohail A, *et al.* Nirmatrelvir–ritonavir for the treatment of COVID-19 patients: A systematic review and meta-analysis. *J Med Virol*. 2023;95. doi: 10.1002/jmv.28471

4 Cheema HA, Jafar U, Elrashedy AA, *et al.* Efficacy and safety of fluvoxamine for the treatment of COVID-19 patients: A systematic review and meta-analysis. J. Infect. 2022;85:702–69. https://doi.org/10.1016/j.jinf.2022.10.012

5 Shafiee A, Athar MMT, Shahid A, *et al.* Curcumin for the treatment of COVID-19 patients: A meta-analysis of randomized controlled trials. *Phyther Res*. 2023;37:1167–75.

6 Cheema HA, Sohail A, Fatima A, *et al.* Quercetin for the treatment of COVID-19 patients: A systematic review and meta-analysis. *Rev Med Virol*. 2023;33:e2427.

7 Shi J, Xiao Y, Zhang Y, *et al.* Challenges of drug development during the COVID-19 pandemic: Key considerations for clinical trial designs. *Br J Clin Pharmacol*. 2021;87:2170–85.

8 Wang J, Levi J, Ellis L, *et al.* Minimum Manufacturing Costs, National Prices, and Estimated Global Availability of New Repurposed Therapies for Coronavirus Disease

For peer review only

2019. *Open Forum Infect Dis*. 2022;9. doi: 10.1093/ofid/ofab581

9 Reyes AZ, Hu KA, Teperman J, *et al.* Anti-inflammatory therapy for COVID-19 infection: the case for colchicine. *Ann Rheum Dis*. 2021;80:550 LP – 557.

10 Page MJ, McKenzie JE, Bossuyt PM, *et al.* The PRISMA 2020 statement: an updated guideline for reporting systematic reviews. *BMJ*. 2021;372:n71.

11 Sterne JAC, Savović J, Page MJ, *et al.* RoB 2: a revised tool for assessing risk of bias in randomised trials. *BMJ*. 2019;366:l4898.

12 Higgins JPT, Thomas J, Chandler J, *et al.*, editors. *Cochrane Handbook for Systematic Reviews of Interventions*. 2nd ed. Hoboken, New Jersey: Wiley Blackwell 2019. https://doi.org/10.1002/9781119536604

13 Perricone C, Scarsi M, Brucato A, *et al.* Treatment with COLchicine in hospitalized patients affected by COVID-19: The COLVID-19 trial. *Eur J Intern Med*. 2023;107:30–6.

14 Rahman M, Datta PK, Islam K, *et al.* Efficacy of colchicine in patients with moderate COVID-19: A double-blinded, randomized, placebo-controlled trial. *PLoS One*. 2022;17:e0277790.

15 Pourdowlat G, Saghafi F, Mozafari A, *et al.* Efficacy and safety of colchicine treatment in patients with COVID-19: A prospective, multicenter, randomized clinical trial. *Phyther Res*. 2022;36:891–8.

16 Absalón-Aguilar A, Rull-Gabayet M, Pérez-Fragoso A, *et al.* Colchicine Is Safe Though Ineffective in the Treatment of Severe COVID-19: a Randomized Clinical Trial (COLCHIVID). *J Gen Intern Med*. 2022;37:4–14.

For peer review only

17 Gorial FI, Maulood MF, Abdulamir AS, *et al.* Randomized controlled trial of colchicine add on to the standard therapy in moderate and severe corona virus Disease-19 infection. *Ann Med Surg*. 2022;77:103593.

18 Lopes MI, Bonjorno LP, Giannini MC, *et al.* Beneficial effects of colchicine for moderate to severe COVID-19: a randomised, double-blinded, placebo-controlled clinical trial. *RMD Open*. 2021;7:e001455.

19 Tardif JC, Bouabdallaoui N, L'Allier PL, *et al.* Colchicine for community-treated patients with COVID-19 (COLCORONA): a phase 3, randomised, double-blinded, adaptive, placebo-controlled, multicentre trial. *Lancet Respir Med*. 2021;9:924–32.

20 Dorward J, Yu LM, Hayward G, *et al.* Colchicine for COVID-19 in the community (PRINCIPLE): a randomised, controlled, adaptive platform trial. *Br J Gen Pract*. 2022;72:E446–55.

21 Recovery Collaborative Group. Colchicine in patients admitted to hospital with COVID-19 (RECOVERY): a randomised, controlled, open-label, platform trial. *Lancet Respir Med*. 2021;9:1419–26.

22 Diaz R, Orlandini A, Castellana N, *et al.* Effect of Colchicine vs Usual Care Alone on Intubation and 28-Day Mortality in Patients Hospitalized with COVID-19: A Randomized Clinical Trial. *JAMA Netw Open*. 2021;4:1–12.

23 Gaitán-Duarte HG, Álvarez-Moreno C, Rincón-Rodríguez CJ, *et al.* Effectiveness of rosuvastatin plus colchicine, emtricitabine/tenofovir and combinations thereof in hospitalized patients with COVID-19: a pragmatic, open-label randomized trial.

For peer review only

eClinicalMedicine. 2022;43:1–12.

24 Jalal A, Aref S, Albustany D. Effectiveness of colchicine among patients with COVID-19 infection: A randomized, open-labeled, clinical trial. *Indian J Rheumatol*. 2022;17:136.

25 Kasiri H, Ghazaiean M, Rouhani N, *et al.* The effects of colchicine on hospitalized COVID-19 patients: A randomized, double-blind, placebo-controlled clinical trial. *J Investig Med*. 2023;10815589221141816.

26 Pascual-Figal DA, Roura-Piloto AE, Moral-Escudero E, *et al.* Colchicine in Recently Hospitalized Patients with COVID-19: A Randomized Controlled Trial (COL-COVID). *Int J Gen Med*. 2021;Volume 14:5517–26.

27 Alsultan M, Obeid A, Alsamarrai O, *et al.* Efficacy of Colchicine and Budesonide in Improvement Outcomes of Patients with Coronavirus Infection 2019 in Damascus, Syria: A Randomized Control Trial. *Interdiscip Perspect Infect Dis*. 2021;2021:1–7.

28 Eikelboom JW, Jolly SS, Belley-Cote EP, *et al.* Colchicine and aspirin in community patients with COVID-19 (ACT): an open-label, factorial, randomised, controlled trial. *Lancet Respir Med*. 2022;10:1160–8.

29 Sunil Naik K, Andhalkar N, Pendse S. Effect of colchicine and aspirin given together in patients with moderate COVID-19. *Contemp Clin Trials Commun*. 2023;101070.

30 Pimenta Bonifácio L, Ramacciotti E, Agati LB, *et al.* Efficacy and Safety of Ixekizumab vs. Low-Dose IL-2 vs. Colchicine vs. Standard of Care on the Treatment of Patients Hospitalized with Moderate to Critical Covid-19: A Pilot Randomized Clinical Trial (STRUCK: Survival Trial Using Cyto kine Inhibitors). *SSRN Electron J*. Published Online

For peer review only

First: 3 May 2022. doi: 10.2139/ssrn.4095747

31 Mostafaie A. Colchicine Plus Phenolic Monoterpenes to Treat COVID-19. ClinicalTrials.gov. https://clinicaltrials.gov/ct2/show/NCT04392141 (accessed 23 January 2023)

32 Salehzadeh F, Pourfarzi F, Ataei S. The Impact of Colchicine on COVID-19 patients: A Clinical Trial Study. *Mediterr J Rheumatol*. 2022;33:232.

33 Deftereos SG, Giannopoulos G, Vrachatis DA, *et al.* Effect of Colchicine vs Standard Care on Cardiac and Inflammatory Biomarkers and Clinical Outcomes in Patients Hospitalized with Coronavirus Disease 2019: The GRECCO-19 Randomized Clinical Trial. *JAMA Netw Open*. 2020;3:1–14.

34 Cecconi A, Martinez-Vives P, Vera A, *et al.* Efficacy of short-course colchicine treatment in hospitalized patients with moderate to severe COVID-19 pneumonia and hyperinflammation: a randomized clinical trial. *Sci Rep*. 2022;12:1–8.

35 Eikelboom JW, Jolly SS, Belley-Cote EP, *et al.* Colchicine and the combination of rivaroxaban and aspirin in patients hospitalised with COVID-19 (ACT): an open-label, factorial, randomised, controlled trial. *Lancet Respir Med*. 2022;19:1–10.

36 Ghaith HS, Gabra MD, Nafady MH, *et al.* A Review of the Rational and Current Evidence on Colchicine for COVID-19. *Curr Pharm Des*. 2022;28:3194–201.

37 Lan S-H, Hsu C-K, Lai C-C, *et al.* Effect of colchicine on the outcomes of patients with COVID-19: a systematic review and meta-analysis of randomised controlled trials. *Ann Med*. 2022;54:1956–65.

For peer review only

38 Toro-Huamanchumo CJ, Benites-Meza JK, Mamani-García CS, *et al.* Efficacy of Colchicine in the Treatment of COVID-19 Patients: A Systematic Review and Meta-Analysis. *J Clin Med*. 2022;11. doi: 10.3390/jcm11092615

39 Bitar AN, Sulaiman SAS. The evidence from clinical trials on colchicine and corticosteroids' effect on COVID-19: a systematic review and meta-analysis. *Curr Med Res Opin*. 2022;38:2097–108.

40 Romeo FJ, Barbagelata L, Chiabrando JG, *et al.* The Effect of Colchicine on Mortality, Mechanical Ventilation, and Length of Stay in Patients With COVID-19 Infection: An Updated Systematic Review and Meta-analysis of Randomized Clinical Trials. *Am J Ther*. 2022;29:e344–50.

41 Yasmin F, Najeeb H, Moeed A, *et al.* Safety and efficacy of colchicine in COVID-19 patients: A systematic review and meta-analysis of randomized control trials. *PLoS One*. 2022;17:e0266245.

42 Zein AFMZ, Raffaello WM. Effect of colchicine on mortality in patients with COVID-19 - A systematic review and meta-analysis. *Diabetes Metab Syndr*. 2022;16:102395.

43 Golpour M, Mousavi T, Alimohammadi M, *et al.* The effectiveness of Colchicine as an anti-inflammatory drug in the treatment of coronavirus disease 2019: Meta-analysis. *Int J Immunopathol Pharmacol*. 2021;35:20587384211031764.

44 Kow CS, Lee L-H, Ramachandram DS, *et al.* The effect of colchicine on mortality outcome and duration of hospital stay in patients with COVID-19: A meta-analysis of randomized trials. *Immunity, Inflamm Dis*. 2022;10:255–64.

For peer review only

45 Danjuma MI, Sayed R, Aboughalia M, *et al.* Does colchicine reduce mortality in patients with COVID-19 clinical syndrome? An umbrella review of published meta-analyses. *Heliyon*. 2023;9:e20155.

46 Pollock M, Fernandes RM, Becker LA, *et al.* Chapter V: Overviews of Reviews. In: Higgins JPT, Thomas J, Chandler J, *et al.*, eds. *Cochrane Handbook for Systematic Reviews of Interventions*. Wiley 2019.

47 Forrest JI, Rayner CR, Park JJH, *et al.* Early Treatment of COVID-19 Disease: A Missed Opportunity. *Infect Dis Ther*. 2020;9:715–20.

For peer review only

Figure legends

Figure 1. PRISMA 2020 flowchart.

Figure 2. Quality assessment of included trials.

Figure 3. Effect of colchicine on all-cause mortality in COVID-19 patients.

For peer review only

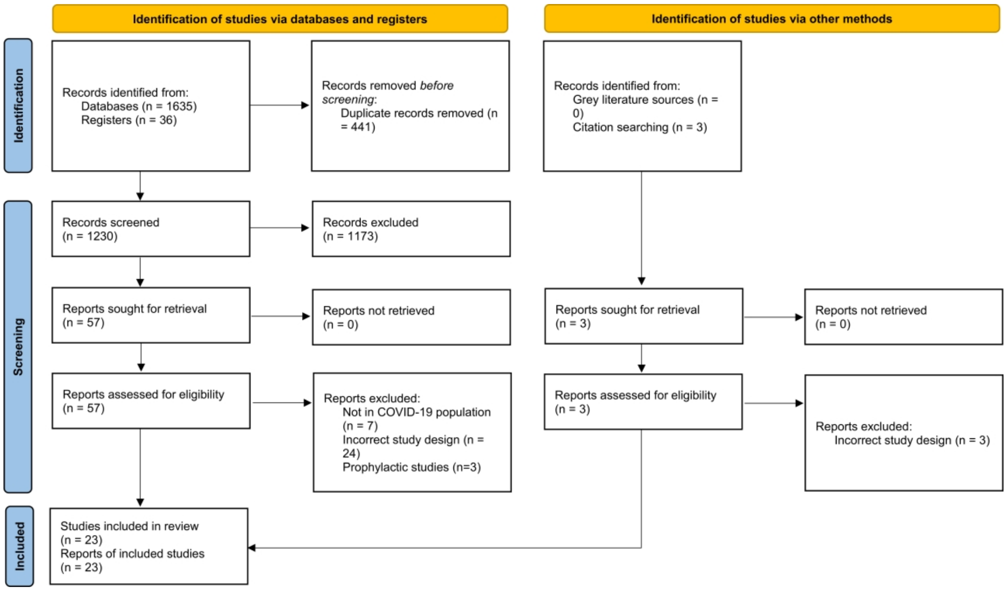

Figure 1. PRISMA 2020 flowchart.

1393x812mm (72 x 72 DPI)

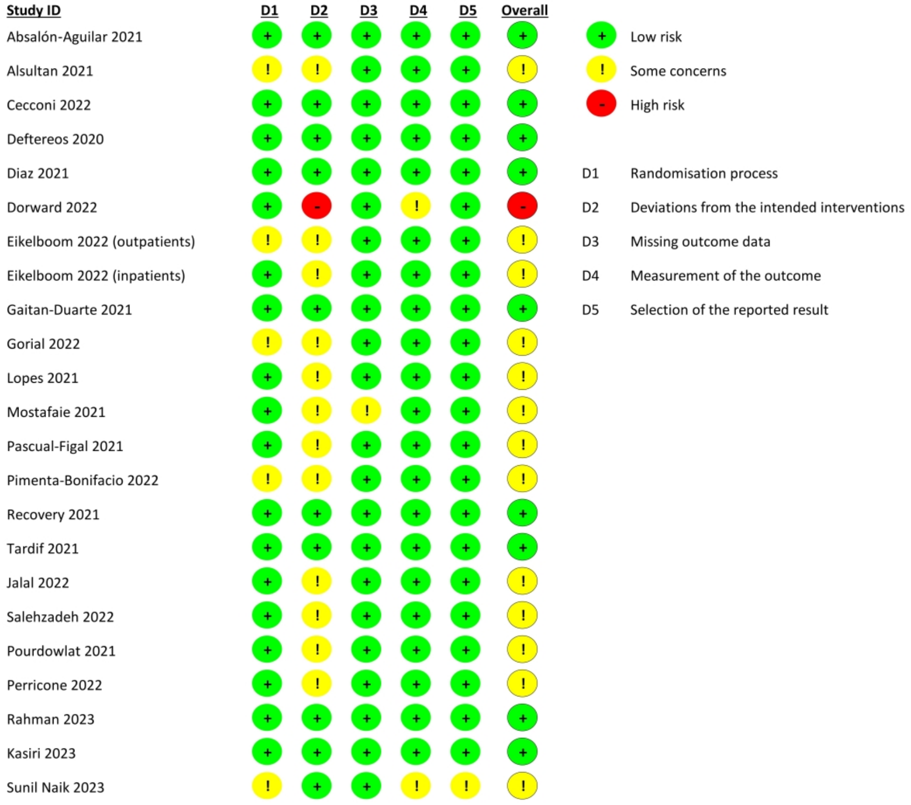

Figure 2. Quality assessment of included trials.

2089x1921mm (72 x 72 DPI)

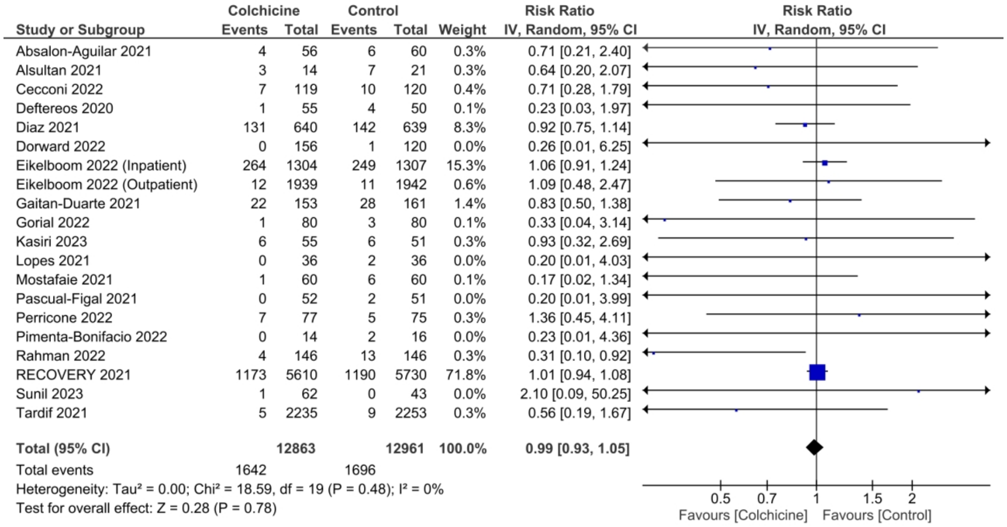

Figure 3. Effect of colchicine on all-cause mortality in COVID-19 patients.

928x493mm (72 x 72 DPI)

Supplementary Material

Colchicine for the treatment of COVID-19 patients: a rapid systematic review and meta-analysis of randomized controlled trials

This supplemental material has been provided by the authors to give readers additional information about their work.

For peer review only

Table S1. PRISMA checklist.

Section and Topic	Item #	Checklist item	Reported on page #
**TITLE**			
Title	1	Identify the report as a systematic review.	1
**ABSTRACT**			
Abstract	2	See the PRISMA 2020 for Abstracts checklist.	3
**INTRODUCTION**			
Rationale	3	Describe the rationale for the review in the context of existing knowledge.	4
Objectives	4	Provide an explicit statement of the objective(s) or question(s) the review addresses.	4
**METHODS**			
Eligibility criteria	5	Specify the inclusion and exclusion criteria for the review and how studies were grouped for the syntheses.	5
Information sources	6	Specify all databases, registers, websites, organisations, reference lists and other sources searched or consulted to identify studies. Specify the date when each source was last searched or consulted.	5
Search strategy	7	Present the full search strategies for all databases, registers and websites, including any filters and limits used.	Suppl.
Selection process	8	Specify the methods used to decide whether a study met the inclusion criteria of the review, including how many reviewers screened each record and each report retrieved, whether they worked independently, and if applicable, details of automation tools used in the process.	5
Data collection process	9	Specify the methods used to collect data from reports, including how many reviewers collected data from each report, whether they worked independently, any processes for obtaining or confirming data from study investigators, and if applicable, details of automation tools used in the process.	5
Data items	10a	List and define all outcomes for which data were sought. Specify whether all results that were compatible with each outcome domain in each study were sought (e.g. for all measures, time points, analyses), and if not, the methods used to decide which results to collect.	5
	10b	List and define all other variables for which data were sought (e.g. participant and intervention characteristics, funding sources). Describe any assumptions made about any missing or unclear information.	5
Study risk of bias assessment	11	Specify the methods used to assess risk of bias in the included studies, including details of the tool(s) used, how many reviewers assessed each study and whether they worked independently, and if applicable, details of automation tools used in the process.	5,6
Effect measures	12	Specify for each outcome the effect measure(s) (e.g. risk ratio, mean difference) used in the synthesis or presentation of results.	6
Synthesis methods	13a	Describe the processes used to decide which studies were eligible for each synthesis (e.g. tabulating the study intervention characteristics and comparing against the planned groups for each synthesis (item #5)).	6

For peer review only

Section and Topic	Item #	Checklist item	Reported on page #
	13b	Describe any methods required to prepare the data for presentation or synthesis, such as handling of missing summary statistics, or data conversions.	6
	13c	Describe any methods used to tabulate or visually display results of individual studies and syntheses.	6
	13d	Describe any methods used to synthesize results and provide a rationale for the choice(s). If meta-analysis was performed, describe the model(s), method(s) to identify the presence and extent of statistical heterogeneity, and software package(s) used.	6
	13e	Describe any methods used to explore possible causes of heterogeneity among study results (e.g. subgroup analysis, meta-regression).	6
	13f	Describe any sensitivity analyses conducted to assess robustness of the synthesized results.	6
Reporting bias assessment	14	Describe any methods used to assess risk of bias due to missing results in a synthesis (arising from reporting biases).	6
Certainty assessment	15	Describe any methods used to assess certainty (or confidence) in the body of evidence for an outcome.	NA
**RESULTS**			
Study selection	16a	Describe the results of the search and selection process, from the number of records identified in the search to the number of studies included in the review, ideally using a flow diagram.	6, Figure 1
	16b	Cite studies that might appear to meet the inclusion criteria, but which were excluded, and explain why they were excluded.	Figure 1
Study characteristics	17	Cite each included study and present its characteristics.	6,7, Table 1
Risk of bias in studies	18	Present assessments of risk of bias for each included study.	7, Figure 2
Results of individual studies	19	For all outcomes, present, for each study: (a) summary statistics for each group (where appropriate) and (b) an effect estimate and its precision (e.g. confidence/credible interval), ideally using structured tables or plots.	Figures, suppl.
Results of syntheses	20a	For each synthesis, briefly summarise the characteristics and risk of bias among contributing studies.	7,8
	20b	Present results of all statistical syntheses conducted. If meta-analysis was done, present for each the summary estimate and its precision (e.g. confidence/credible interval) and measures of statistical heterogeneity. If comparing groups, describe the direction of the effect.	7,8
	20c	Present results of all investigations of possible causes of heterogeneity among study results.	7
	20d	Present results of all sensitivity analyses conducted to assess the robustness of the synthesized results.	7
Reporting	21	Present assessments of risk of bias due to missing results (arising from reporting biases) for each synthesis assessed.	7

For peer review only

For peer review only - http://bmjopen.bmj.com/site/about/guidelines.xhtml

Section and Topic	Item #	Checklist item	Reported on page #
biases			
Certainty of evidence	22	Present assessments of certainty (or confidence) in the body of evidence for each outcome assessed.	NA
**DISCUSSION**			
Discussion	23a	Provide a general interpretation of the results in the context of other evidence.	8-10
	23b	Discuss any limitations of the evidence included in the review.	9
	23c	Discuss any limitations of the review processes used.	9
	23d	Discuss implications of the results for practice, policy, and future research.	9,10
**OTHER INFORMATION**			
Registration and protocol	24a	Provide registration information for the review, including register name and registration number, or state that the review was not registered.	4
	24b	Indicate where the review protocol can be accessed, or state that a protocol was not prepared.	4
	24c	Describe and explain any amendments to information provided at registration or in the protocol.	NA
Support	25	Describe sources of financial or non-financial support for the review, and the role of the funders or sponsors in the review.	10
Competing interests	26	Declare any competing interests of review authors.	10
Availability of data, code and other materials	27	Report which of the following are publicly available and where they can be found: template data collection forms; data extracted from included studies; data used for all analyses; analytic code; any other materials used in the review.	10

For peer review only

Table S2. Search strategy for electronic databases.

**MEDLINE (PubMed)**	("colchicine"[MeSH Terms] OR "colchicine"[tiab]) AND ("COVID-19"[MeSH Terms] OR "COVID-19"[tiab] OR "SARS-CoV-2"[tiab] OR "coronavirus disease 2019"[tiab]) AND ("randomized controlled trial"[pt] OR "controlled clinical trial"[pt] OR "randomized"[tiab] OR "placebo"[tiab] OR "drug therapy"[Subheading] OR "therapeutics"[tiab] OR "treatment"[tiab] OR "intervention"[tiab] OR "clinical trial"[pt] OR "placebo-controlled trial"[tiab])
**Embase**	'colchicine'/exp OR 'colchicine':ab,ti AND ('COVID-19'/exp OR 'COVID-19':ab,ti OR 'SARS-CoV-2':ab,ti OR 'coronavirus disease 2019':ab,ti) AND ('clinical trial'/exp OR 'randomized controlled trial'/exp OR 'placebo'/exp OR 'double-blind procedure'/exp OR 'single-blind procedure'/exp OR 'clinical trial phase III'/exp OR 'clinical trial phase IV'/exp OR 'randomization'/exp OR 'placebo-controlled study'/exp OR 'crossover procedure'/exp OR 'factorial design'/exp OR 'control group'/exp)
**The Cochrane Library**	(("Colchicine"[Mesh]) OR ("colchicine"[tiab])) AND (("COVID-19"[Mesh]) OR ("SARS-CoV-2"[Mesh]) OR ("Coronavirus Disease 2019"[Mesh]) OR ("COVID-19"[tiab]) OR ("SARS-CoV-2"[tiab]) OR ("coronavirus disease 2019"[tiab]))

For peer review only

Table S3. Characteristics on included trials.

Sr No	Author, year	Country	Sample size	Age*	Sex, n (%)	Population	Intervention	Comparator	Immunization Status
1	Absalón-Aguilar 2021	Mexico	116	53 (44–62)	Male: 76 (65.5)<br>Female: 40 (35.5)	Hospitalized COVID-19 adult patients with severe disease	1.5 mg of colchicine at the time of the recruitment in the study and 0.5 mg BID to complete 10 days of treatment	Placebo	NR
2	Alsultan 2021	Syria	49 (35 were eligible as 14 received budesonide)	-	-	Hospitalized, moderate-to-severe ARDS, adult patients with either a positive PCR test of COVID-19 virus in specimens taken from the respiratory tracts or a negative PCR test but with clinical signs and symptoms of viral illness along with findings on chest CT compatible with COVID-19 infection	1.5 mg of colchicine followed by 0.5 mg after an hour on day 1, then 0.5 mg twice daily for the next 4 days	Supportive care only	NR
3	Cecconi 2022	Spain	239	65.1 ± 16.0	Male: 141 (59)<br>Female: 98 (41)	Hospitalized moderate-to-severe COVID-19 patients	5 days of oral colchicine: 1 mg loading dose and then 0.5 mg/day	Placebo	NR

For peer review only

4	Deftereos 2020	Greece	105	64 (54-76)	Male: 61 (58.1) Female: 44 (41.9)	Hospitalized adult COVID-19 patients	The loading dose consisted of 1.5 mg of colchicine followed by 0.5 mg of colchicine 60 minutes later. The maintenance dosage was 0.5 mg colchicine twice daily until hospital discharge or a maximum of 21 days.	No colchicine	NR
5	Diaz 2021	Argentina	1279	61.8 ± 14.6	Male: 830 (64.9) Female: 449 (35.1)	Hospitalized severe COVID-19 patients	Colchicine was administered orally in a loading dose of 1.5 mg followed by 0.5 mg orally within 2 hours of the initial dose and 0.5 mg orally twice a day for 14 days or discharge, whichever occurred first.	Usual care with no colchicine	NR
6	Dorward 2022	UK	2293 (156 received colchicine)	-	-	Non-hospitalized COVID-19 patients	Colchicine 500 µg daily for 14 days	Usual care	Both vaccinated and unvaccinated population
7	Eikelboom 2022 (inpatients)	11 countries	2611	Colchicine: 56.1 ± 16.7 Control: 56.0 ± 16.0	Colchicine: Male: 762 (58.4), Female: 542 (31.6) Control: Male: 796 (60.9), Female: 511 (39.1)	Hospitalized COVID-19 patients	Colchicine 1.2 mg followed by 0.6 mg 2h later and then 0.6 mg twice daily in tablet form for 28 days	Usual care	Both vaccinated and unvaccinated population

For peer review only

8	Eikelboom 2022 (outpatients)	11 countries	3917	Colchicine: 45.0 ± 13.7 Control: 45.0 ± 13.3	Colchicine: Male: 1173 (60.5%) Female 766 (39.5%) Control: Male: 1177 (60·6%) Female 765 (39.4%)	Non-hospitalized COVID-19 patients	Oral colchicine 0.6 mg twice daily for 3 days and then 0.6 mg once daily for 25 days	Usual care	Both vaccinated and unvaccinated population
9	Gaitan-Duarte 2021	Bogota, Colombia	314	Colchicine + Rosuvastatin: 56.1 ± 13.2 Control: 55.3 ± 12.3	Colchicine: Male: 103 (68), Female: 50 (32) Standard care: Male 113 (70.2), Female 48 (29.8)	Hospitalized adult COVID-19 patients	Colchicine plus rosuvastatin (0.5 mg and 40 mg for 14 days)	Standard of care	NR
10	Gorial 2022	Iraq	160	-	-	Hospitalized moderate-to-severe COVID-19 patients	Colchicine 1 mg daily orally for 7 days, then 0.5 mg daily for another 7 days	Standard treatment	NR
11	Jalal 2022	Iraq	80	-	Colchicine: Male: 21 (52.5), Female: 19 (47.5) Control: Male: 19 (47.5), Female: 21 (52.5)	Patients with mild, moderate, or severe COVID-19 infection; either hospitalized or at home	Colchicine 0.5 mg twice daily for 14 days	Standard treatment	NR
12	Kasiri 2023	Iran	106	54.62 ± 13.92	Male: 49 (46.2) Female: 57 (53.8)	Hospitalized adult severe COVID-19 patients	Colchicine 2 mg loading dose followed by 0.5 mg twice daily for 7 days	Placebo	NR
13	Lopes 2021	Brazil	75	Colchicine group: 54.5 (42.5–64.5) Placebo: 55.0 (42.0–67.0)	Colchicine: Male 19 (53), Female: 17 (47) Placebo: Male: 14 (39), Female: 22 (61)	Hospitalized moderate-to-severe COVID-19 patients	Colchicine 0.5 mg thrice daily for 5 days, then 0.5mg twice daily for 5 days	Placebo	NR

For peer review only

14	Mostafaie, unpublished	Iran	120	-	-	Hospitalized COVID-19 patients aged ≥ 10 years	Colchicine plus herbal phenolic monoterpene	Standard treatment	NR
15	Pascual-Figal 2021	Spain	103	51 ± 12	Male: 54 (52.4) Female: 49 (47.6)	Hospitalized adult COVID-19 patients	Colchicine 1.5 mg loading dose, followed by 0.5 mg BID for one week and 0.5 mg per day for 28 days	Standard treatment	NR
16	Perricone 2023	Italy	152	Colchicine: 69.1 ± 13.1 Control: 67.9 ± 15	Colchicine: Male: 47 (61), Female: 30 (39) Control: Male: 50 (67), Female: 25 (33)	Hospitalized adult COVID-19 patients	Colchicine 0.5 mg three times a day if weight was less than 100 kg or 1 mg twice a day if weight was more than 100 kg for a maximum of 30 days or until hospital discharge	Standard treatment	Unvaccinated population only
17	Pimenta Bonifácio 2022	Brazil	30	Colchicine: 56.22 ± 11.6 Control: 46.11 ± 10.6	Colchicine: Male: 10 (71), Female: 4 (29) Control: Male: 11 (69), Female 5 (31)	Hospitalized moderate-to-severe COVID-19 patients	Colchicine 0.5 mg every 8 hours for three days, followed by 0.5mg 2 complete four weeks of treatment	Standard of care	Unvaccinated population only
18	Pourdowlat 2021	Iran	165	54.72 ± 15.03	Male: 93 (63) Female: 72 (37)	Non-hospitalized moderate-to-severe COVID-19 patients	A 3-day course of 0.5 mg colchicine followed by a 12-day course of 1 mg colchicine	Standard of care	NR

For peer review only

19	Rahman 2022	Bangladesh	299	47 (35–55)	Male: 190 (64.2) Female: 106 (35.8)	Hospitalized moderate COVID-19 patients	1.2 mg of colchicine on day 1 followed by daily treatment with 0.6 mg of colchicine for 13 days	Placebo	NR
20	RECOVERY 2021	UK, Indonesia, Nepal	11340	63.4 ± 13·8	Colchicine: Male: 3897 (69), Female: 1713 (31) Control group: Male: 4012 (70), Female: 1718 (30)	Hospitalized moderate-to-severe adult COVID-19 patients	Colchicine 1 mg followed by 500 µg 12h later and then 500 µg twice a day by mouth or nasogastric tube for 10 days in total or until discharge	Standard of care	NR
21	Salehzadeh 2022	Iran	100	Colchicine: 56.56 ± 17.16 Placebo: 55.56 ± 16.38	Male: 41 (41) Female 59 (59)	Hospitalized moderate COVID-19 patients	Colchicine 1 mg daily for six days	Placebo	NR
22	Sunil Naik 2023	India	122	-	-	Hospitalized moderate COVID-19 patients	Colchicine 0.5 mg BID	Standard care	NR
23	Tardif 2021	Brazil, Canada, Greece, South Africa, Spain, and the USA	4488	54·0 (47·0–61·0)	Colchicine: Males: 997 (44.6), Females: 1238 (55.4) Control: Males: 1070 (47.5), Female: 1183 (52.5)	Non-hospitalized COVID-19 patients aged ≥ 40 years	Colchicine 0·5 mg BID for the first 3 days and then once per day for 27 days	Placebo	NR

For peer review only

*Data reported as mean ± SD and median (IQR)

BID, twice daily; ARDS, acute respiratory distress syndrome; PCR, polymerase chain reaction: NR, Not reported

For peer review only

Supplementary Figure S1. Funnel plot for all-cause mortality.

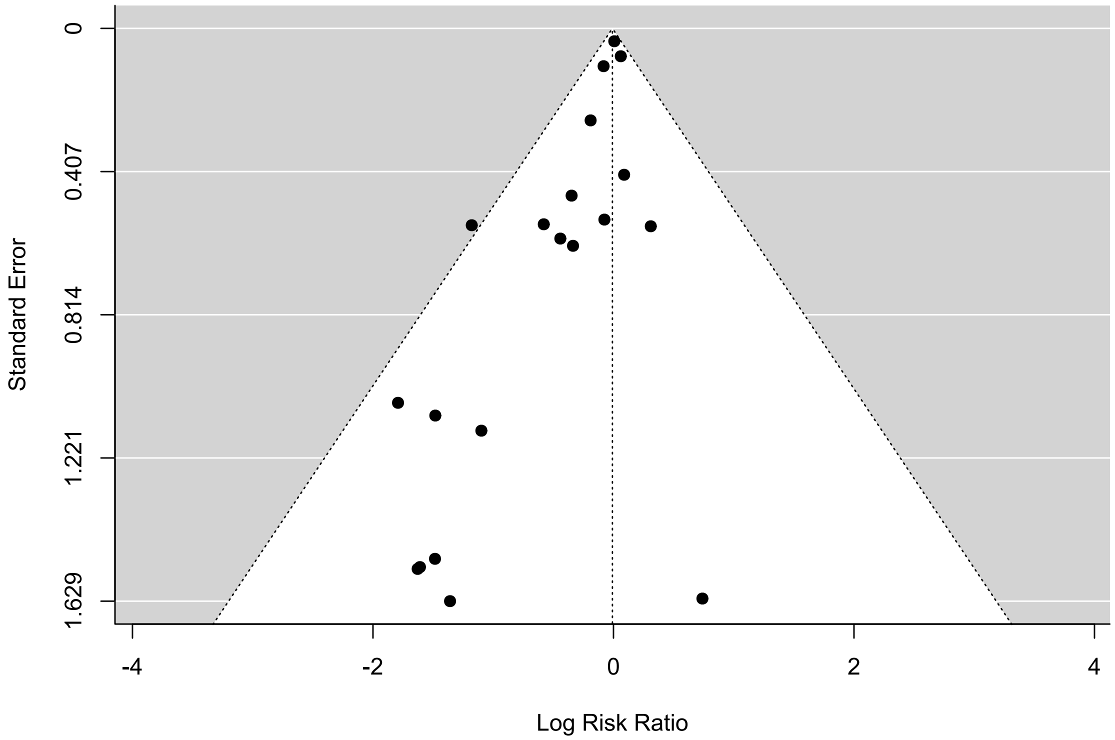

Supplementary Figure S2. Subgroup analysis of the effect of colchicine on the risk of mortality.

Study or Subgroup	Colchicine Events	Colchicine Total	Control Events	Control Total	Weight	Risk Ratio IV, Random, 95% CI
**1.6.1 Hospitalized**						
Absalon-Aguilar 2021	4	56	6	60	0.3%	0.71 [0.21, 2.40]
Alsultan 2021	3	14	7	21	0.3%	0.64 [0.20, 2.07]
Cecconi 2022	7	119	10	120	0.4%	0.71 [0.28, 1.79]
Deftereos 2020	1	55	4	50	0.1%	0.23 [0.03, 1.97]
Diaz 2021	131	640	142	639	8.3%	0.92 [0.75, 1.14]
Eikelboom 2022 (Inpatient)	264	1304	249	1307	15.3%	1.06 [0.91, 1.24]
Gaitan-Duarte 2021	22	153	28	161	1.4%	0.83 [0.50, 1.38]
Gorial 2022	1	80	3	80	0.1%	0.33 [0.04, 3.14]
Kasiri 2023	6	55	6	51	0.3%	0.93 [0.32, 2.69]
Lopes 2021	0	36	2	36	0.0%	0.20 [0.01, 4.03]
Mostafaie 2021	1	60	6	60	0.1%	0.17 [0.02, 1.34]
Pascual-Figal 2021	0	52	2	51	0.0%	0.20 [0.01, 3.99]
Perricone 2022	7	77	5	75	0.3%	1.36 [0.45, 4.11]
Pimenta-Bonifacio 2022	0	14	2	16	0.0%	0.23 [0.01, 4.36]
Rahman 2022	4	146	13	146	0.3%	0.31 [0.10, 0.92]
RECOVERY 2021	1173	5610	1190	5730	71.8%	1.01 [0.94, 1.08]
Sunil 2023	1	62	0	43	0.0%	2.10 [0.09, 50.25]
**Subtotal (95% CI)**		**8533**		**8646**	**99.1%**	**0.98 [0.90, 1.06]**
Total events	1625		1675			
Heterogeneity: Tau² = 0.00; Chi² = 16.79, df = 16 (P = 0.40); I² = 5%						
Test for overall effect: Z = 0.47 (P = 0.64)						
**1.6.2 Non-hospitalized**						
Dorward 2022	0	156	1	120	0.0%	0.26 [0.01, 6.25]
Eikelboom 2022 (Outpatient)	12	1939	11	1942	0.6%	1.09 [0.48, 2.47]
Tardif 2021	5	2235	9	2253	0.3%	0.56 [0.19, 1.67]
**Subtotal (95% CI)**		**4330**		**4315**	**0.9%**	**0.82 [0.43, 1.55]**
Total events	17		21			
Heterogeneity: Tau² = 0.00; Chi² = 1.45, df = 2 (P = 0.48); I² = 0%						
Test for overall effect: Z = 0.61 (P = 0.54)						
**Total (95% CI)**		**12863**		**12961**	**100.0%**	**0.99 [0.93, 1.05]**
Total events	1642		1696			
Heterogeneity: Tau² = 0.00; Chi² = 18.59, df = 19 (P = 0.48); I² = 0%						
Test for overall effect: Z = 0.28 (P = 0.78)						
Test for subgroup differences: Chi² = 0.30, df = 1 (P = 0.59), I² = 0%						

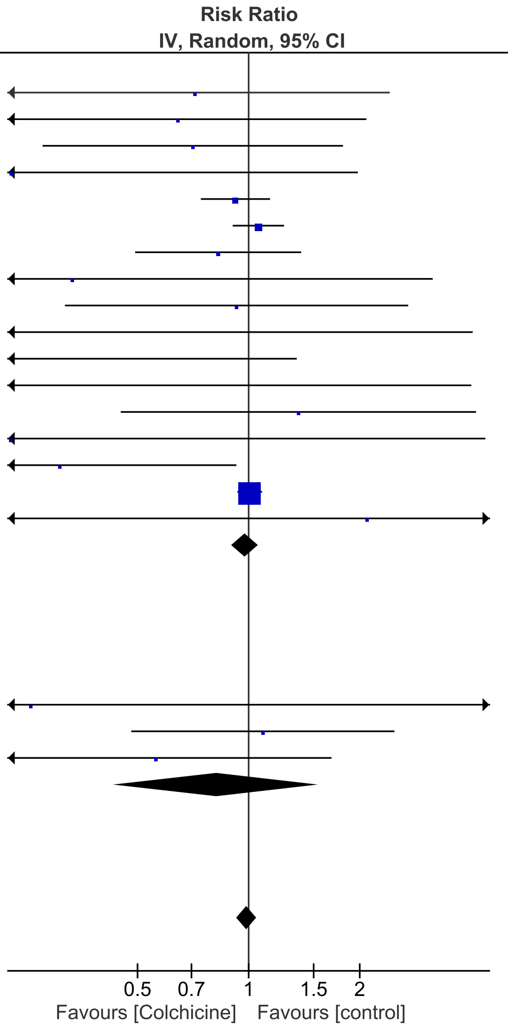

Supplementary Figure S3. Forest plot of the effect of colchicine on the risk of mechanical ventilation and ICU admission.

Study or Subgroup	Colchicine Events	Colchicine Total	Control Events	Control Total	Weight	Risk Ratio IV, Random, 95% CI
**1.2.1 Risk of mechanical ventilation**						
Cecconi 2022	5	119	10	120	12.5%	0.50 [0.18, 1.43]
Deftereos 2020	1	55	5	50	4.0%	0.18 [0.02, 1.50]
Kasiri 2023	6	55	6	51	12.2%	0.93 [0.32, 2.69]
Pascual-Figal 2021	0	52	2	51	2.1%	0.20 [0.01, 3.99]
Perricone 2022	4	77	3	75	7.6%	1.30 [0.30, 5.61]
Rahman 2022	2	146	4	146	6.0%	0.50 [0.09, 2.69]
RECOVERY 2021	259	3815	228	3962	36.4%	1.18 [0.99, 1.40]
Tardif 2021	11	2235	21	2253	19.2%	0.53 [0.26, 1.09]
**Subtotal (95% CI)**		**6554**		**6708**	**100.0%**	**0.75 [0.48, 1.18]**
Total events	288		279			
Heterogeneity: Tau² = 0.14; Chi² = 11.69, df = 7 (P = 0.11); I² = 40%						
Test for overall effect: Z = 1.23 (P = 0.22)						
**1.2.2 ICU admission**						
Cecconi 2022	11	119	14	120	37.4%	0.79 [0.38, 1.67]
Kasiri 2023	12	55	9	51	34.7%	1.24 [0.57, 2.69]
Lopes 2021	2	36	4	36	7.8%	0.50 [0.10, 2.56]
Pascual-Figal 2021	2	52	4	51	7.7%	0.49 [0.09, 2.56]
Perricone 2022	1	77	4	75	4.4%	0.24 [0.03, 2.13]
Rahman 2022	2	146	5	146	7.9%	0.40 [0.08, 2.03]
**Subtotal (95% CI)**		**485**		**479**	**100.0%**	**0.77 [0.49, 1.22]**
Total events	30		40			
Heterogeneity: Tau² = 0.00; Chi² = 3.70, df = 5 (P = 0.59); I² = 0%						
Test for overall effect: Z = 1.10 (P = 0.27)						

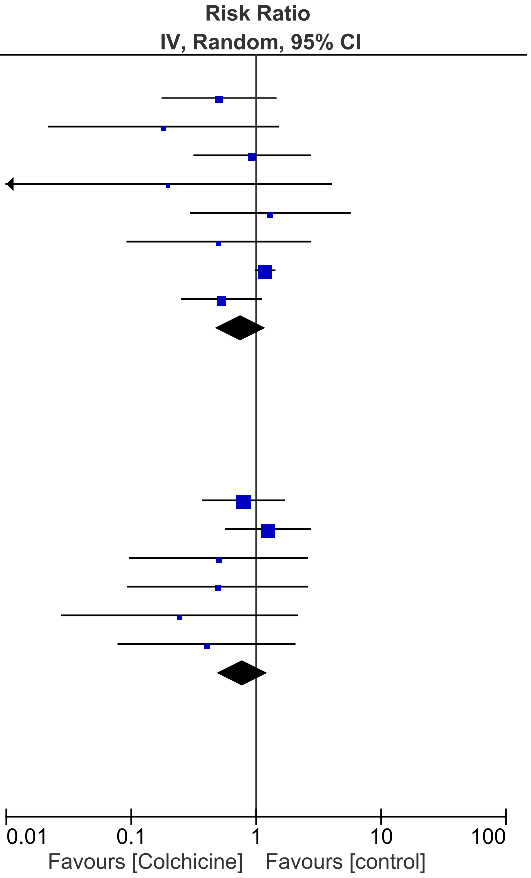

eview only

Supplementary Figure S4. Forest plot of the effect of colchicine on the risk of hospitalization.

	Colchicine		Control			Risk Ratio
**Study or Subgroup**	**Events**	**Total**	**Events**	**Total**	**Weight**	**IV, Random, 95% CI**
Eikelboom 2022 (Outpatient)	62	1939	61	1942	39.7%	1.02 [0.72, 1.44]
Pourdowlat 2021	5	102	18	100	15.5%	0.27 [0.11, 0.71]
Tardif 2021	101	2235	128	2253	44.8%	0.80 [0.62, 1.03]
**Total (95% CI)**		**4276**		**4295**	**100.0%**	**0.74 [0.48, 1.16]**
Total events	168		207			

Heterogeneity: Tau² = 0.10; Chi² = 6.69, df = 2 (P = 0.04); I² = 70%
Test for overall effect: Z = 1.30 (P = 0.19)

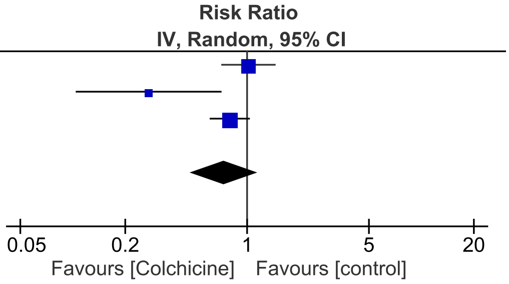

For peer review only

Supplementary Figure S5. Forest plot of the effect of colchicine on the length of hospital stay.

	Colchicine			Control				Mean Difference
**Study or Subgroup**	**Mean**	**SD**	**Total**	**Mean**	**SD**	**Total**	**Weight**	**IV, Random, 95% CI**
Absalon-Aguilar 2021	7.916	4.26	56	8.33	4.07	60	13.2%	-0.41 [-1.93, 1.10]
Jalal 2022	18.4	5.81	40	24.24	8.63	40	9.3%	-5.84 [-9.06, -2.62]
Lopes 2021	7	2.96	36	9.33	3.7	36	13.1%	-2.33 [-3.88, -0.78]
Mostafaie 2021	6.39	2.59	54	4.17	1.34	59	14.4%	2.22 [1.45, 2.99]
Pascual-Figal 2021	6.6	3.86	52	5.76	4.89	51	12.8%	0.84 [-0.86, 2.54]
Perricone 2022	14.1	10.4	77	14.7	8.1	75	9.9%	-0.60 [-3.56, 2.36]
Rahman 2022	10.67	5.93	146	10	6.67	146	13.3%	0.67 [-0.78, 2.12]
Salehzadeh 2022	6.28	2.51	50	8.12	2.66	50	14.1%	-1.84 [-2.85, -0.83]
**Total (95% CI)**			**511**			**517**	**100.0%**	**-0.70 [-2.30, 0.89]**

Heterogeneity: Tau² = 4.44; Chi² = 67.53, df = 7 (P < 0.00001); I² = 90%
Test for overall effect: Z = 0.86 (P = 0.39)

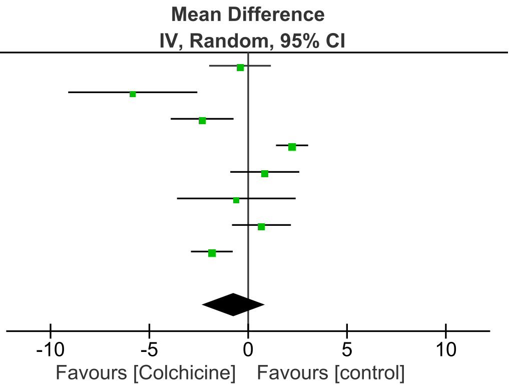

For peer review only

Supplementary Figure S6. Forest plot of the effect of colchicine on the rate of no recovery.

Study or Subgroup	Colchicine Events	Colchicine Total	Control Events	Control Total	Weight	Risk Ratio IV, Random, 95% CI
Dorward 2022	42	156	306	1145	25.3%	1.01 [0.76, 1.33]
Gorial 2022	12	80	32	80	14.8%	0.38 [0.21, 0.67]
Kasiri 2023	7	55	9	51	8.5%	0.72 [0.29, 1.79]
Pourdowlat 2021	25	102	40	100	20.1%	0.61 [0.40, 0.93]
RECOVERY 2021	1709	5610	1698	5730	31.3%	1.03 [0.97, 1.09]
**Total (95% CI)**		**6003**		**7106**	**100.0%**	**0.77 [0.56, 1.05]**
Total events	1795		2085			

Heterogeneity: Tau² = 0.08; Chi² = 17.39, df = 4 (P = 0.002); I² = 77%

Test for overall effect: Z = 1.65 (P = 0.10)

Risk Ratio
IV, Random, 95% CI

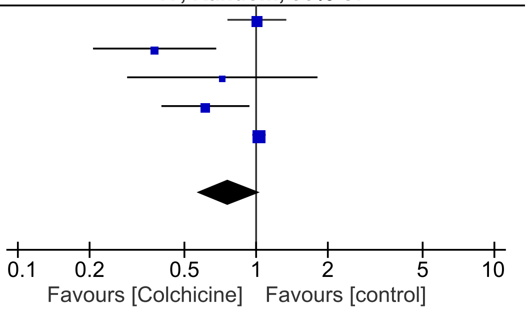

For peer review only

PRISMA 2020 checklist

Section and Topic	Item #	Checklist item	Reported on page #
**TITLE**			
Title	1	Identify the report as a systematic review.	1
**ABSTRACT**			
Abstract	2	See the PRISMA 2020 for Abstracts checklist.	3
**INTRODUCTION**			
Rationale	3	Describe the rationale for the review in the context of existing knowledge.	4
Objectives	4	Provide an explicit statement of the objective(s) or question(s) the review addresses.	4
**METHODS**			
Eligibility criteria	5	Specify the inclusion and exclusion criteria for the review and how studies were grouped for the syntheses.	5
Information sources	6	Specify all databases, registers, websites, organisations, reference lists and other sources searched or consulted to identify studies. Specify the date when each source was last searched or consulted.	5
Search strategy	7	Present the full search strategies for all databases, registers and websites, including any filters and limits used.	Suppl.
Selection process	8	Specify the methods used to decide whether a study met the inclusion criteria of the review, including how many reviewers screened each record and each report retrieved, whether they worked independently, and if applicable, details of automation tools used in the process.	5
Data collection process	9	Specify the methods used to collect data from reports, including how many reviewers collected data from each report, whether they worked independently, any processes for obtaining or confirming data from study investigators, and if applicable, details of automation tools used in the process.	5
Data items	10a	List and define all outcomes for which data were sought. Specify whether all results that were compatible with each outcome domain in each study were sought (e.g. for all measures, time points, analyses), and if not, the methods used to decide which results to collect.	5
	10b	List and define all other variables for which data were sought (e.g. participant and intervention characteristics, funding sources). Describe any assumptions made about any missing or unclear information.	5
Study risk of bias assessment	11	Specify the methods used to assess risk of bias in the included studies, including details of the tool(s) used, how many reviewers assessed each study and whether they worked independently, and if applicable, details of automation tools used in the process.	5,6
Effect measures	12	Specify for each outcome the effect measure(s) (e.g. risk ratio, mean difference) used in the synthesis or presentation of results.	6
Synthesis methods	13a	Describe the processes used to decide which studies were eligible for each synthesis (e.g. tabulating the study intervention characteristics and comparing against the planned groups for each synthesis (item #5)).	6

For peer review only

Section and Topic	Item #	Checklist item	Reported on page #
	13b	Describe any methods required to prepare the data for presentation or synthesis, such as handling of missing summary statistics, or data conversions.	6
	13c	Describe any methods used to tabulate or visually display results of individual studies and syntheses.	6
	13d	Describe any methods used to synthesize results and provide a rationale for the choice(s). If meta-analysis was performed, describe the model(s), method(s) to identify the presence and extent of statistical heterogeneity, and software package(s) used.	6
	13e	Describe any methods used to explore possible causes of heterogeneity among study results (e.g. subgroup analysis, meta-regression).	6
	13f	Describe any sensitivity analyses conducted to assess robustness of the synthesized results.	6
Reporting bias assessment	14	Describe any methods used to assess risk of bias due to missing results in a synthesis (arising from reporting biases).	6
Certainty assessment	15	Describe any methods used to assess certainty (or confidence) in the body of evidence for an outcome.	NA
**RESULTS**			
Study selection	16a	Describe the results of the search and selection process, from the number of records identified in the search to the number of studies included in the review, ideally using a flow diagram.	6, Figure 1
	16b	Cite studies that might appear to meet the inclusion criteria, but which were excluded, and explain why they were excluded.	Figure 1
Study characteristics	17	Cite each included study and present its characteristics.	6,7, Table 1
Risk of bias in studies	18	Present assessments of risk of bias for each included study.	7, Figure 2
Results of individual studies	19	For all outcomes, present, for each study: (a) summary statistics for each group (where appropriate) and (b) an effect estimate and its precision (e.g. confidence/credible interval), ideally using structured tables or plots.	Figures, suppl.
Results of syntheses	20a	For each synthesis, briefly summarise the characteristics and risk of bias among contributing studies.	7,8
	20b	Present results of all statistical syntheses conducted. If meta-analysis was done, present for each the summary estimate and its precision (e.g. confidence/credible interval) and measures of statistical heterogeneity. If comparing groups, describe the direction of the effect.	7,8
	20c	Present results of all investigations of possible causes of heterogeneity among study results.	7
	20d	Present results of all sensitivity analyses conducted to assess the robustness of the synthesized results.	7
Reporting	21	Present assessments of risk of bias due to missing results (arising from reporting biases) for each synthesis assessed.	7

For peer review only

Section and Topic	Item #	Checklist item	Reported on page #
biases			
Certainty of evidence	22	Present assessments of certainty (or confidence) in the body of evidence for each outcome assessed.	NA
**DISCUSSION**			
Discussion	23a	Provide a general interpretation of the results in the context of other evidence.	8-10
	23b	Discuss any limitations of the evidence included in the review.	9
	23c	Discuss any limitations of the review processes used.	9
	23d	Discuss implications of the results for practice, policy, and future research.	9,10
**OTHER INFORMATION**			
Registration and protocol	24a	Provide registration information for the review, including register name and registration number, or state that the review was not registered.	4
	24b	Indicate where the review protocol can be accessed, or state that a protocol was not prepared.	4
	24c	Describe and explain any amendments to information provided at registration or in the protocol.	NA
Support	25	Describe sources of financial or non-financial support for the review, and the role of the funders or sponsors in the review.	10
Competing interests	26	Declare any competing interests of review authors.	10
Availability of data, code and other materials	27	Report which of the following are publicly available and where they can be found: template data collection forms; data extracted from included studies; data used for all analyses; analytic code; any other materials used in the review.	10

For peer review only

BMJ Open

Colchicine for the treatment of patients with COVID-19: an updated systematic review and meta-analysis of randomized controlled trials

Journal:	*BMJ Open*
Manuscript ID	bmjopen-2023-074373.R2
Article Type:	Original research
Date Submitted by the Author:	28-Mar-2024
Complete List of Authors:	Cheema, Huzaifa Ahmad ; King Edward Medical University, Department of Medicine<br>Jafar, Uzair; King Edward Medical University, Department of Medicine<br>Shahid, Abia; King Edward Medical University, Department of Medicine<br>Masood, Waniyah; Dow University of Health Sciences, Department of Medicine<br>Usman, Muhammad; King Edward Medical University, Department of Medicine<br>Hermis, Alaa Hamza; Al-Mustaqbal University College<br>Naseem, Muhammad Arsal; King Edward Medical University, Department of Medicine<br>Sahra, Syeda; The University of Oklahoma Health Sciences Center, Department of Infectious Diseases<br>Sah, Ranjit; Tribhuvan University Teaching Hospital; Harvard Medical School<br>Lee, Ka Yiu; Mid Sweden University, Department of Health Sciences
**Primary Subject Heading**:	Infectious diseases
Secondary Subject Heading:	Immunology (including allergy)
Keywords:	COVID-19, VIROLOGY, CLINICAL PHARMACOLOGY

SCHOLARONE™
Manuscripts

I, the Submitting Author has the right to grant and does grant on behalf of all authors of the Work (as defined in the below author licence), an exclusive licence and/or a non-exclusive licence for contributions from authors who are: i) UK Crown employees; ii) where BMJ has agreed a CC-BY licence shall apply, and/or iii) in accordance with the terms applicable for US Federal Government officers or employees acting as part of their official duties; on a worldwide, perpetual, irrevocable, royalty-free basis to BMJ Publishing Group Ltd ("BMJ") its licensees and where the relevant Journal is co-owned by BMJ to the co-owners of the Journal, to publish the Work in this journal and any other BMJ products and to exploit all rights, as set out in our licence.

The Submitting Author accepts and understands that any supply made under these terms is made by BMJ to the Submitting Author unless you are acting as an employee on behalf of your employer or a postgraduate student of an affiliated institution which is paying any applicable article publishing charge ("APC") for Open Access articles. Where the Submitting Author wishes to make the Work available on an Open Access basis (and intends to pay the relevant APC), the terms of reuse of such Open Access shall be governed by a Creative Commons licence – details of these licences and which Creative Commons licence will apply to this Work are set out in our licence referred to above.

Other than as permitted in any relevant BMJ Author's Self Archiving Policies, I confirm this Work has not been accepted for publication elsewhere, is not being considered for publication elsewhere and does not duplicate material already published. I confirm all authors consent to publication of this Work and authorise the granting of this licence.

For peer review only

Colchicine for the treatment of patients with COVID-19: an updated systematic review and meta-analysis of randomized controlled trials

Huzaifa Ahmad Cheema*[1]; Uzair Jafar*[1]; Abia Shahid[1]; Waniyah Masood[2]; Muhammad Usman[1]; Alaa Hamza Hermis[3]; Muhammad Arsal Naseem[1]; Syeda Sahra[4]; Ranjit Sah[5,6]; Ka Yiu Lee[7]

*These authors contributed equally and are co-first authors

1. Division of Infectious Diseases, Department of Medicine, King Edward Medical University, Lahore, Pakistan.
2. Department of Medicine, Dow University of Health Sciences, Karachi, Pakistan.
3. Al-Mustaqbal University College, 51001 Hillah, Babylon, Iraq.
4. Department of Infectious Diseases, University of Oklahoma Health Sciences Center, Oklahoma City, OK, United States.
5. Tribhuvan University Teaching Hospital, Institute of Medicine, Kathmandu, Nepal.
6. Harvard Medical School, Boston, MA, United States.
7. Swedish Winter Sports Research Centre, Department of Health Sciences, Mid Sweden University, Östersund, Sweden.

Correspondence to:

Ka Yiu Lee

Swedish Winter Sports Research Centre, Department of Health Sciences, Mid Sweden University, Östersund, Sweden

For peer review only

kyle.lee@miun.se

or

Huzaifa Ahmad Cheema

Division of Infectious Diseases, Department of Medicine, King Edward Medical University, Nila Gumbad Chowk, Neela Gumbad Lahore, Punjab 54000, Pakistan

huzaifacheema@kemu.edu.pk;

For peer review only

Word count: 1899

Abstract

Objectives: We conducted an updated systematic review and meta-analysis to investigate the effect of colchicine treatment on clinical outcomes in patients with COVID-19.

Design: Systematic review and meta-analysis.

Data sources: We searched PubMed, Embase, the Cochrane Library, medRxiv, and ClinicalTrials.gov from inception to January 2023.

Eligibility criteria: All randomized controlled trials (RCTs) that investigated the efficacy of colchicine treatment in patients with COVID-19 as compared with placebo or standard of care were included. There were no language restrictions. Studies that used colchicine prophylactically were excluded.

Data extraction and synthesis: We extracted all information relating to the study characteristics, such as author names, location, study population, details of intervention and comparator groups, and our outcomes of interest. We conducted our meta-analysis using RevMan 5.4 with risk ratio (RR) and mean difference (MD) as the effect measures.

Results: We included 23 RCTs (28249 participants) in this systematic review. Colchicine did not decrease the risk of mortality (RR 0.99; 95% CI: 0.93-1.05; I^2=0%; 20 RCTs, 25824 participants), with the results being consistent among both hospitalized and non-hospitalized patients. There were no significant differences between the colchicine and control groups in other relevant clinical outcomes, including the incidence of mechanical ventilation (RR 0.75; 95% CI: 0.48-1.18; p=0.22; I^2=40%; eight RCTs, 13262 participants), ICU admission (RR 0.77; 95% CI: 0.49-1.22; p=0.27; I^2=0%; six RCTs, 961 participants), and hospital admission (RR 0.74; 95% CI: 0.48-1.16; p=0.19; I_2=70%; three RCTs, 8572 participants).

For peer review only

Conclusions: The results of this meta-analysis do not support the use of colchicine as a treatment for reducing the risk of mortality or improving other relevant clinical outcomes in patients with COVID-19. However, RCTs investigating early treatment with colchicine (within 5 days of symptom onset or in patients with early-stage disease) are needed to fully elucidate the potential benefits of colchicine in this patient population.

Study registration: PROSPERO, CRD42022369850.

For peer review only

Keywords: colchicine; COVID-19; SARS-CoV-2; meta-analysis

Strengths and limitations of this study

- This systematic review used a comprehensive search for articles evaluating colchicine treatment for patients with COVID-19.
- We performed, to our knowledge, the largest meta-analysis to date by pooling results from 23 trials.
- There was significant interstudy heterogeneity in the overall analysis of some outcomes, such as length of hospitalization.
- Our results may not be generalizable to the use of colchicine early in the course of the disease as most RCTs studied late-stage treatment.

For peer review only

INTRODUCTION

Unprecedented global research has been conducted as a result of the COVID-19 pandemic. Even though particular antiviral medications, immunomodulators, and monoclonal antibodies have been approved to treat COVID-19,[1–3] ongoing clinical trials are being conducted to combat the virus more successfully, particularly using repurposed drugs and herbal medicines that provide a cheap therapeutic alternative.[4–6] Limited knowledge of COVID-19 molecular mechanisms and pathophysiology, lack of definitive supporting evidence, and expensive costs of some treatment options are some of the challenges faced in using different drugs for COVID-19.[7,8]

Colchicine, an anti-inflammatory medication commonly used to treat gout, recurrent pericarditis, and familial Mediterranean fever, is being extensively investigated among COVID-19 patients.[9] Inhibiting neutrophil chemotaxis, suppressing inflammasome signaling, and preventing the cytokine storm through microtubule depolarization are some of the potential benefits of colchicine in treating COVID-19.[9] Currently, guidelines from the World Health Organization (WHO) recommend against treatment with colchicine based on data from 10 studies.[1]

Although colchicine has been the subject of multiple clinical trials in COVID-19 patients, findings from smaller randomized controlled trials (RCTs) that were positive were later contradicted by results from major RCTs.[9] Therefore, we performed this meta-analysis to evaluate the safety and efficacy of colchicine for the treatment of COVID-19 patients and to interpret the significance of the latest data by incorporating it into our review.

For peer review only

METHODS

Our systematic review and meta-analysis was registered with PROSPERO (CRD42022369850) and conducted by following the recommendations of Preferred Reporting Items for Systematic Reviews and Meta-Analyses (PRISMA).[10] The PRISMA checklist is provided in Supplementary Table 1.

Data sources and search strategy

We searched MEDLINE (via PubMed), Embase, the Cochrane Library, medRxiv, and ClinicalTrials.gov from inception to January 2023 using a search strategy consisting of terms related to “colchicine” and “COVID-19” without any restrictions. Grey literature and the reference lists of the relevant studies were also explored to include all eligible trials. We did not restrict our search based on language. The detailed search strategy for different databases is given in Supplementary Table 2.

Eligibility criteria

The inclusion criteria were: 1) population: patients with a diagnosis of COVID-19; 2) intervention: colchicine regardless of dosing or regimen; 3) comparator: placebo or standard of care; 4) outcome: reporting any outcome of interest; and 5) study design: RCTs only. Observational studies, quasi-randomized trials, and studies that used colchicine prophylactically were excluded.

Study selection

All the literature obtained from our searches was imported into Mendeley Desktop 1.19.8 and duplicates were removed. The remaining articles were subjected to a rigorous screening process

For peer review only

by two independent reviewers in a two-stage process (title/abstract screening followed by full-text screening).

Data extraction and outcomes

We extracted all information relating to the study characteristics such as author names, location, study population, details of intervention and comparator groups, and our outcomes of interest. We chose all-cause mortality as our primary outcome while the secondary outcomes included the incidence of mechanical ventilation (MV), risk of intensive care unit (ICU) admission, risk of hospitalization, the length of hospital stay, and the rate of no recovery (proportion of patients with no symptomatic resolution at the end of study).

Quality assessment

The risk of bias was assessed using the revised Cochrane Risk of Bias tool (RoB 2.0).[11] A rating of low risk of bias, some concerns, or a high risk of bias was assigned to each study using five domains: randomization process, deviations from intended interventions, missing outcome data, measurement of the outcome, and selection of reported result.

Data analysis

We conducted our meta-analysis using RevMan 5.4 with risk ratio (RR) and mean difference (MD) as the effect measures for categorical and continuous variables, respectively. We utilized a random-effects model as we anticipated our included studies to be substantially heterogeneous. We evaluated heterogeneity using the Chi^2 test, the I^2 statistic, and the Tau^2 estimate. We used the Cochrane Handbook for Systematic Reviews of Intervention to interpret the values of the I^2 statistic.[12] We conducted a subgroup analysis for our primary outcome on the basis of the study population (hospitalized vs. non-hospitalized patients). In addition, we conducted a

For peer review only - http://bmjopen.bmj.com/site/about/guidelines.xhtml

sensitivity analysis on our primary outcome by excluding trials that used colchicine in combination with another intervention. For outcomes with data from 10 studies or more, we assessed publication bias by constructing funnel plots and running Egger's test for funnel plot asymmetry.

Patient and public involvement

None.

RESULTS

Search results and study characteristics

A total of 23 RCTs (28249 participants) were eligible for inclusion in our meta-analysis.[13–35] The details of the screening process are presented in Figure 1.

17 RCTs were conducted in hospitalized patients, four evaluated outpatients only,[15,19,20,28] and one RCT included both hospitalized and non-hospitalized patients.[24] Most of the trials had small sample sizes while the RECOVERY trial was the largest RCT with 11,340 patients.[21] The trials employed a variety of colchicine regimens and most were open-label that used standard of care as the comparator. Two RCTs combined colchicine with other treatments; one with rosuvastatin,[23] and the other with herbal phenolic monoterpene.[31] The characteristics of each RCT are summarized in Supplementary Table 3.

Risk of bias assessment

According to RoB 2.0, seven studies were at a low risk of bias, one had a high risk of bias, and 12 had some concerns of bias (Figure 2). The most common source of bias was lack of blinding and deviations from protocol while some trials also had issues in the randomization process.

For peer review only - http://bmjopen.bmj.com/site/about/guidelines.xhtml

Results of the meta-analysis

Primary outcome

Our analysis, considering 25,824 patients from 20 RCTs, failed to find a statistically significant reduction in the risk of mortality with colchicine use (RR 0.99; 95% CI: 0.93-1.05; p=0.78; I^2=0%; Figure 3). The Egger's test indicated that publication bias was present (p = 0.002; Supplementary Figure 1). The results were consistent ($P_{interaction}$ = 0.59) among both hospitalized (RR 0.98; 95% CI: 0.90-1.06; p=0.64; I^2=5%) and non-hospitalized patients (RR 0.82; 95% CI: 0.43-1.55; p=0.54; I^2=0%; Supplementary Figure 2).

Sensitivity analysis by excluding two studies that used combination therapies in the intervention group,[23,31] produced results consistent with our main analysis (RR 1.00; 95% CI: 0.94-1.06; p=0.88; I^2=0%). The results remained congruous with our primary analysis after performing a second sensitivity analysis excluding studies with a high risk of bias or some concerns of bias in the domain of deviations from intended interventions (RR 0.97; 95% CI: 0.88-1.06; p=0.46; I^2=3%).

Secondary outcomes

There were no significant differences between the colchicine and control groups in the incidence of MV (RR 0.75; 95% CI: 0.48-1.18; p=0.22; I^2=40%; eight RCTs, 13262 participants; Supplementary Figure 3), risk of ICU admission (RR 0.77; 95% CI: 0.49-1.22; p=0.27; I^2=0%; six RCTs, 961 participants; Supplementary Figure 3), risk of hospital admission (RR 0.74; 95% CI: 0.48-1.16; p=0.19; I_2=70%; three RCTs, 8572 participants; Supplementary Figure 4), length of hospital stay (MD -0.70 days; 95% CI: -2.30 to 0.89 days; p=0.39; I^2=90%; eight RCTs, 1028 participants; Supplementary Figure 5), and the rate of no recovery (RR 0.77; 95% CI: 0.56-1.05; p=0.10; I^2=77%; five RCTs, 13109 participants; Supplementary Figure 6).

For peer review only

DISCUSSION

To the best of our knowledge, this is the largest and most up-to-date meta-analysis to assess the effectiveness of colchicine for COVID-19. The results of this meta-analysis suggest that colchicine does not have a significant effect on the risk of mortality in patients with COVID-19. There was also no significant benefit of colchicine in reducing the risk of mechanical ventilation, ICU admission, hospital admission, or improving recovery rates.

Colchicine's regulation of the inflammatory response has been proposed as a means to inhibit the inflammation caused by COVID-19. The primary mechanism by which colchicine affects cells is through the inhibition of microtubule polymerization, which has a ripple effect on various cellular functions such as maintaining cell shape, signaling, cell division, migration, and transport.[9,36] Additionally, colchicine modulates the synthesis of inflammatory cytokines such as tumor necrosis factor (TNF), interleukin-1 (IL-1), and interleukin-6 (IL-6) and disrupts the activation of inflammasomes, neutrophil adhesion, and recruitment.[9,36] Given that COVID-19 is characterized by excessive inflammation and a cytokine response, colchicine's anti-inflammatory effects may have the potential to improve outcomes.

Our results are consistent with some of the previous meta-analyses which also demonstrated no significant clinical benefit of colchicine in COVID-19 patients.[37–41] On the other hand, the meta-analyses by Zein et al. and Golpour et al. [42,43] found that colchicine use was associated with a reduction in the risk of mortality, and Kow et al. demonstrated a shorter duration of hospital stay with colchicine.[44] However, the meta-analysis by Zein et al. and Golpour et al. also incorporated observational studies, which increases the likelihood of confounding bias in their findings.[42,43] Additionally, all of the previous meta-analyses included a small number of RCTs with most including less than or equal to eleven.[45] Conversely, our meta-analysis pooled

For peer review only

results from 23 RCTs, therefore, including a significantly greater cumulative sample size and substantially increasing the confidence in our estimates.

A recent umbrella review found that exposure to colchicine was associated with reduced mortality.[45] However, this discrepancy can be explained by the fact that it pooled estimates from meta-analyses instead of primary studies. Since there is a significant overlap in the included studies between the prior meta-analyses, pooling meta-analyzed effect sizes leads to erroneous and flawed results due to double-counting of outcome data. The correct approach would be to synthesize data from the primary studies to provide valid estimates.[46]

It is worth noting that despite including a large number of RCTs, the results of our meta-analysis are still greatly influenced by the RECOVERY trial.[21] The RECOVERY trial is by far the largest trial of colchicine which found no clinical benefit of colchicine in COVID-19 patients. However, this was a very late-stage treatment RCT with the median time from symptom onset to treatment initiation being 9 days. It is well recognized that late initiation of therapy might result in decreased effectiveness of antivirals.[47] Therefore, early treatment (within 5 days of symptom onset or at an early stage of the disease) with colchicine might prove to be beneficial for COVID-19 patients; however, this needs to be investigated in future RCTs as all of the currently available RCTs evaluated late-stage treatment.

Some limitations of our meta-analysis should be noted. The quality of the RCTs included in the meta-analysis was mixed, with nine being of high quality, one being of low quality, and 13 having some concerns of bias. This may have affected the reliability of the results. Additionally, there was significant interstudy heterogeneity in the overall analysis of some outcomes, such as length of hospitalization. Our results may not be generalizable to the usage of colchicine early in

For peer review only

the course of the disease as most RCTs are of late-stage treatment; consequently, there was not enough data to compare early- versus late-stage treatment. Furthermore, the composite endpoint of death or mechanical ventilation is an important outcome; however, due to a lack of data provided by the RCTs, we were unable to analyze this endpoint. Moreover, many studies either did not report the vaccination status of their participants or included unvaccinated patients only. Therefore, the COVID-19 vaccination status as a potential effect modifier needs to be studied further. Finally, there was evidence of the presence of publication bias in our meta-analysis implying that small studies that did not report positive findings may not have been published.

CONCLUSION

The results of this meta-analysis do not support the use of colchicine as a treatment for reducing the risk of mortality or improving other clinical outcomes in patients with COVID-19. However, RCTs investigating early treatment with colchicine (within 5 days of symptom onset or in patients with early-stage disease) are needed to fully elucidate the potential benefits of colchicine in this patient population.

For peer review only

Contributors

Conception and design of study: H.A. Cheema, A. Shahid, K.Y. Lee, U. Jafar. Acquisition of data: M. Usman, M. A. Naseem, H.A. Cheema. Data analysis and/or interpretation: S. Sahra, R. Sah, U. Jafar, A. A. Hermis, W. Masood. Drafting or writing of the manuscript: M. A. Naseem, U. Jafar, S. Sahra, M. Usman, A. Shahid, W. Masood. Substantial revision or critical review of the manuscript: H.A. Cheema, A.A. Hermis, R. Sah, K.Y. Lee. All authors have approved the final submitted version of the manuscript.

Funding

No financial support was received for this study.

Competing interests

The authors report no relationships that could be construed as a conflict of interest.

Data availability statement

The data that support the findings of this study are available from the corresponding author upon reasonable request.

Ethics approval

Not applicable.

Patient consent for publication

Not required.

For peer review only

References

1 Agarwal A, Rochwerg B, Lamontagne F, *et al.* A living WHO guideline on drugs for covid-19. *BMJ*. 2020;370:m3379.

2 Fatima M, Azeem S, Saeed J, *et al.* Efficacy and safety of molnupiravir for COVID-19 patients. *Eur J Intern Med*. 2022;102:118–21.

3 Cheema HA, Jafar U, Sohail A, *et al.* Nirmatrelvir–ritonavir for the treatment of COVID-19 patients: A systematic review and meta-analysis. *J Med Virol*. 2023;95. doi: 10.1002/jmv.28471

4 Cheema HA, Jafar U, Elrashedy AA, *et al.* Efficacy and safety of fluvoxamine for the treatment of COVID-19 patients: A systematic review and meta-analysis. J. Infect. 2022;85:702–69. https://doi.org/10.1016/j.jinf.2022.10.012

5 Shafiee A, Athar MMT, Shahid A, *et al.* Curcumin for the treatment of COVID-19 patients: A meta-analysis of randomized controlled trials. *Phyther Res*. 2023;37:1167–75.

6 Cheema HA, Sohail A, Fatima A, *et al.* Quercetin for the treatment of COVID-19 patients: A systematic review and meta-analysis. *Rev Med Virol*. 2023;33:e2427.

7 Shi J, Xiao Y, Zhang Y, *et al.* Challenges of drug development during the COVID-19 pandemic: Key considerations for clinical trial designs. *Br J Clin Pharmacol*. 2021;87:2170–85.

8 Wang J, Levi J, Ellis L, *et al.* Minimum Manufacturing Costs, National Prices, and Estimated Global Availability of New Repurposed Therapies for Coronavirus Disease

For peer review only

2019. *Open Forum Infect Dis*. 2022;9. doi: 10.1093/ofid/ofab581

9 Reyes AZ, Hu KA, Teperman J, *et al.* Anti-inflammatory therapy for COVID-19 infection: the case for colchicine. *Ann Rheum Dis*. 2021;80:550 LP – 557.

10 Page MJ, McKenzie JE, Bossuyt PM, *et al.* The PRISMA 2020 statement: an updated guideline for reporting systematic reviews. *BMJ*. 2021;372:n71.

11 Sterne JAC, Savović J, Page MJ, *et al.* RoB 2: a revised tool for assessing risk of bias in randomised trials. *BMJ*. 2019;366:l4898.

12 Higgins JPT, Thomas J, Chandler J, *et al.*, editors. *Cochrane Handbook for Systematic Reviews of Interventions*. 2nd ed. Hoboken, New Jersey: Wiley Blackwell 2019. https://doi.org/10.1002/9781119536604

13 Perricone C, Scarsi M, Brucato A, *et al.* Treatment with COLchicine in hospitalized patients affected by COVID-19: The COLVID-19 trial. *Eur J Intern Med*. 2023;107:30–6.

14 Rahman M, Datta PK, Islam K, *et al.* Efficacy of colchicine in patients with moderate COVID-19: A double-blinded, randomized, placebo-controlled trial. *PLoS One*. 2022;17:e0277790.

15 Pourdowlat G, Saghafi F, Mozafari A, *et al.* Efficacy and safety of colchicine treatment in patients with COVID-19: A prospective, multicenter, randomized clinical trial. *Phyther Res*. 2022;36:891–8.

16 Absalón-Aguilar A, Rull-Gabayet M, Pérez-Fragoso A, *et al.* Colchicine Is Safe Though Ineffective in the Treatment of Severe COVID-19: a Randomized Clinical Trial (COLCHIVID). *J Gen Intern Med*. 2022;37:4–14.

For peer review only

17 Gorial FI, Maulood MF, Abdulamir AS, *et al.* Randomized controlled trial of colchicine add on to the standard therapy in moderate and severe corona virus Disease-19 infection. *Ann Med Surg*. 2022;77:103593.

18 Lopes MI, Bonjorno LP, Giannini MC, *et al.* Beneficial effects of colchicine for moderate to severe COVID-19: a randomised, double-blinded, placebo-controlled clinical trial. *RMD Open*. 2021;7:e001455.

19 Tardif JC, Bouabdallaoui N, L'Allier PL, *et al.* Colchicine for community-treated patients with COVID-19 (COLCORONA): a phase 3, randomised, double-blinded, adaptive, placebo-controlled, multicentre trial. *Lancet Respir Med*. 2021;9:924–32.

20 Dorward J, Yu LM, Hayward G, *et al.* Colchicine for COVID-19 in the community (PRINCIPLE): a randomised, controlled, adaptive platform trial. *Br J Gen Pract*. 2022;72:E446–55.

21 Recovery Collaborative Group. Colchicine in patients admitted to hospital with COVID-19 (RECOVERY): a randomised, controlled, open-label, platform trial. *Lancet Respir Med*. 2021;9:1419–26.

22 Diaz R, Orlandini A, Castellana N, *et al.* Effect of Colchicine vs Usual Care Alone on Intubation and 28-Day Mortality in Patients Hospitalized with COVID-19: A Randomized Clinical Trial. *JAMA Netw Open*. 2021;4:1–12.

23 Gaitán-Duarte HG, Álvarez-Moreno C, Rincón-Rodríguez CJ, *et al.* Effectiveness of rosuvastatin plus colchicine, emtricitabine/tenofovir and combinations thereof in hospitalized patients with COVID-19: a pragmatic, open-label randomized trial.

For peer review only

eClinicalMedicine. 2022;43:1–12.

24 Jalal A, Aref S, Albustany D. Effectiveness of colchicine among patients with COVID-19 infection: A randomized, open-labeled, clinical trial. *Indian J Rheumatol*. 2022;17:136.

25 Kasiri H, Ghazaiean M, Rouhani N, *et al.* The effects of colchicine on hospitalized COVID-19 patients: A randomized, double-blind, placebo-controlled clinical trial. *J Investig Med*. 2023;10815589221141816.

26 Pascual-Figal DA, Roura-Piloto AE, Moral-Escudero E, *et al.* Colchicine in Recently Hospitalized Patients with COVID-19: A Randomized Controlled Trial (COL-COVID). *Int J Gen Med*. 2021;Volume 14:5517–26.

27 Alsultan M, Obeid A, Alsamarrai O, *et al.* Efficacy of Colchicine and Budesonide in Improvement Outcomes of Patients with Coronavirus Infection 2019 in Damascus, Syria: A Randomized Control Trial. *Interdiscip Perspect Infect Dis*. 2021;2021:1–7.

28 Eikelboom JW, Jolly SS, Belley-Cote EP, *et al.* Colchicine and aspirin in community patients with COVID-19 (ACT): an open-label, factorial, randomised, controlled trial. *Lancet Respir Med*. 2022;10:1160–8.

29 Sunil Naik K, Andhalkar N, Pendse S. Effect of colchicine and aspirin given together in patients with moderate COVID-19. *Contemp Clin Trials Commun*. 2023;101070.

30 Pimenta Bonifácio L, Ramacciotti E, Agati LB, *et al.* Efficacy and Safety of Ixekizumab vs. Low-Dose IL-2 vs. Colchicine vs. Standard of Care on the Treatment of Patients Hospitalized with Moderate to Critical Covid-19: A Pilot Randomized Clinical Trial (STRUCK: Survival Trial Using Cyto kine Inhibitors). *SSRN Electron J*. Published Online

For peer review only

First: 3 May 2022. doi: 10.2139/ssrn.4095747

31 Mostafaie A. Colchicine Plus Phenolic Monoterpenes to Treat COVID-19. ClinicalTrials.gov. https://clinicaltrials.gov/ct2/show/NCT04392141 (accessed 23 January 2023)

32 Salehzadeh F, Pourfarzi F, Ataei S. The Impact of Colchicine on COVID-19 patients: A Clinical Trial Study. *Mediterr J Rheumatol*. 2022;33:232.

33 Deftereos SG, Giannopoulos G, Vrachatis DA, *et al.* Effect of Colchicine vs Standard Care on Cardiac and Inflammatory Biomarkers and Clinical Outcomes in Patients Hospitalized with Coronavirus Disease 2019: The GRECCO-19 Randomized Clinical Trial. *JAMA Netw Open*. 2020;3:1–14.

34 Cecconi A, Martinez-Vives P, Vera A, *et al.* Efficacy of short-course colchicine treatment in hospitalized patients with moderate to severe COVID-19 pneumonia and hyperinflammation: a randomized clinical trial. *Sci Rep*. 2022;12:1–8.

35 Eikelboom JW, Jolly SS, Belley-Cote EP, *et al.* Colchicine and the combination of rivaroxaban and aspirin in patients hospitalised with COVID-19 (ACT): an open-label, factorial, randomised, controlled trial. *Lancet Respir Med*. 2022;19:1–10.

36 Ghaith HS, Gabra MD, Nafady MH, *et al.* A Review of the Rational and Current Evidence on Colchicine for COVID-19. *Curr Pharm Des*. 2022;28:3194–201.

37 Lan S-H, Hsu C-K, Lai C-C, *et al.* Effect of colchicine on the outcomes of patients with COVID-19: a systematic review and meta-analysis of randomised controlled trials. *Ann Med*. 2022;54:1956–65.

For peer review only

38 Toro-Huamanchumo CJ, Benites-Meza JK, Mamani-García CS, *et al.* Efficacy of Colchicine in the Treatment of COVID-19 Patients: A Systematic Review and Meta-Analysis. *J Clin Med*. 2022;11. doi: 10.3390/jcm11092615

39 Bitar AN, Sulaiman SAS. The evidence from clinical trials on colchicine and corticosteroids' effect on COVID-19: a systematic review and meta-analysis. *Curr Med Res Opin*. 2022;38:2097–108.

40 Romeo FJ, Barbagelata L, Chiabrando JG, *et al.* The Effect of Colchicine on Mortality, Mechanical Ventilation, and Length of Stay in Patients With COVID-19 Infection: An Updated Systematic Review and Meta-analysis of Randomized Clinical Trials. *Am J Ther*. 2022;29:e344–50.

41 Yasmin F, Najeeb H, Moeed A, *et al.* Safety and efficacy of colchicine in COVID-19 patients: A systematic review and meta-analysis of randomized control trials. *PLoS One*. 2022;17:e0266245.

42 Zein AFMZ, Raffaello WM. Effect of colchicine on mortality in patients with COVID-19 - A systematic review and meta-analysis. *Diabetes Metab Syndr*. 2022;16:102395.

43 Golpour M, Mousavi T, Alimohammadi M, *et al.* The effectiveness of Colchicine as an anti-inflammatory drug in the treatment of coronavirus disease 2019: Meta-analysis. *Int J Immunopathol Pharmacol*. 2021;35:20587384211031764.

44 Kow CS, Lee L-H, Ramachandram DS, *et al.* The effect of colchicine on mortality outcome and duration of hospital stay in patients with COVID-19: A meta-analysis of randomized trials. *Immunity, Inflamm Dis*. 2022;10:255–64.

For peer review only

45 Danjuma MI, Sayed R, Aboughalia M, *et al.* Does colchicine reduce mortality in patients with COVID-19 clinical syndrome? An umbrella review of published meta-analyses. *Heliyon*. 2023;9:e20155.

46 Pollock M, Fernandes RM, Becker LA, *et al.* Chapter V: Overviews of Reviews. In: Higgins JPT, Thomas J, Chandler J, *et al.*, eds. *Cochrane Handbook for Systematic Reviews of Interventions*. Wiley 2019.

47 Forrest JI, Rayner CR, Park JJH, *et al.* Early Treatment of COVID-19 Disease: A Missed Opportunity. *Infect Dis Ther*. 2020;9:715–20.

For peer review only

Figure titles

Figure 1. PRISMA 2020 flowchart

Figure 2. Quality assessment of included trials

Figure 3. Effect of colchicine on all-cause mortality in patients with COVID-19

For peer review only

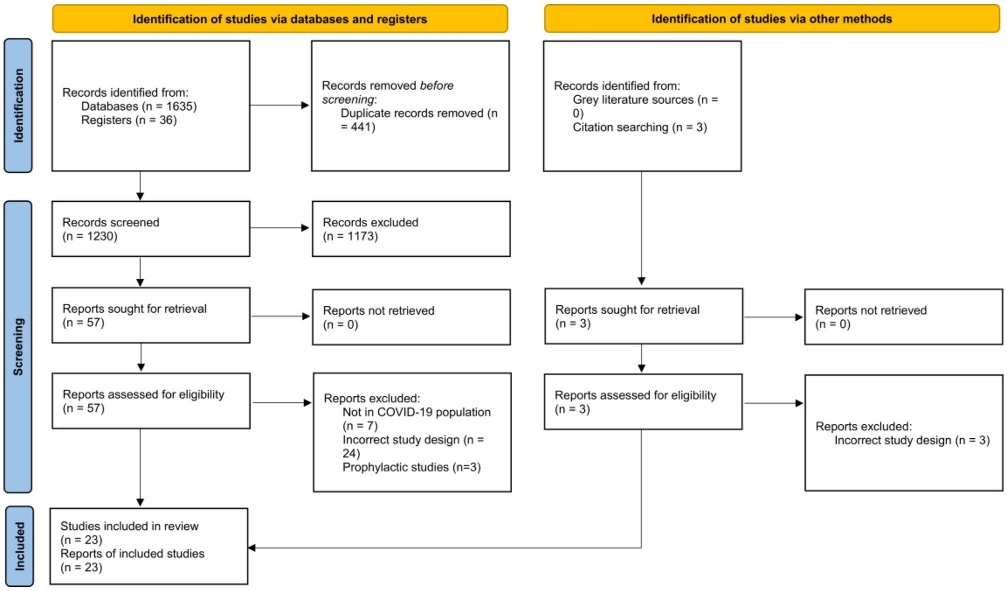

Figure 1. PRISMA 2020 flowchart.

1393x812mm (72 x 72 DPI)

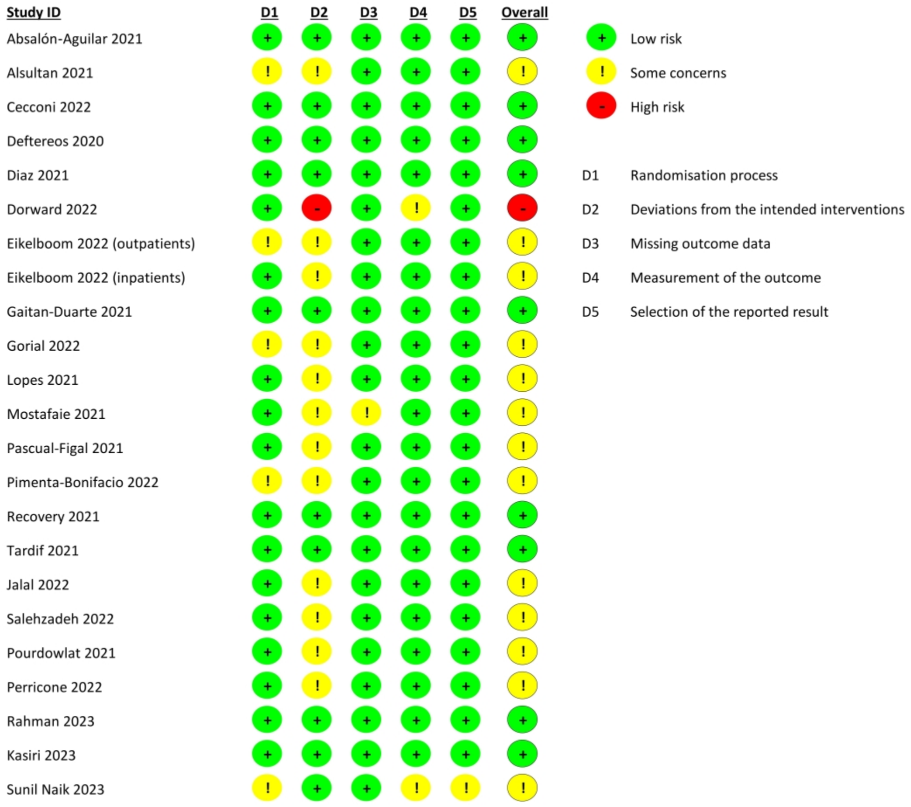

Figure 2. Quality assessment of included trials.

2089x1921mm (72 x 72 DPI)

Study or Subgroup	Colchicine Events	Colchicine Total	Control Events	Control Total	Weight	Risk Ratio IV, Random, 95% CI
Absalon-Aguilar 2021	4	56	6	60	0.3%	0.71 [0.21, 2.40]
Alsultan 2021	3	14	7	21	0.3%	0.64 [0.20, 2.07]
Cecconi 2022	7	119	10	120	0.4%	0.71 [0.28, 1.79]
Deftereos 2020	1	55	4	50	0.1%	0.23 [0.03, 1.97]
Diaz 2021	131	640	142	639	8.3%	0.92 [0.75, 1.14]
Dorward 2022	0	156	1	120	0.0%	0.26 [0.01, 6.25]
Eikelboom 2022 (Inpatient)	264	1304	249	1307	15.3%	1.06 [0.91, 1.24]
Eikelboom 2022 (Outpatient)	12	1939	11	1942	0.6%	1.09 [0.48, 2.47]
Gaitan-Duarte 2021	22	153	28	161	1.4%	0.83 [0.50, 1.38]
Gorial 2022	1	80	3	80	0.1%	0.33 [0.04, 3.14]
Kasiri 2023	6	55	6	51	0.3%	0.93 [0.32, 2.69]
Lopes 2021	0	36	2	36	0.0%	0.20 [0.01, 4.03]
Mostafaie 2021	1	60	6	60	0.1%	0.17 [0.02, 1.34]
Pascual-Figal 2021	0	52	2	51	0.0%	0.20 [0.01, 3.99]
Perricone 2022	7	77	5	75	0.3%	1.36 [0.45, 4.11]
Pimenta-Bonifacio 2022	0	14	2	16	0.0%	0.23 [0.01, 4.36]
Rahman 2022	4	146	13	146	0.3%	0.31 [0.10, 0.92]
RECOVERY 2021	1173	5610	1190	5730	71.8%	1.01 [0.94, 1.08]
Sunil 2023	1	62	0	43	0.0%	2.10 [0.09, 50.25]
Tardif 2021	5	2235	9	2253	0.3%	0.56 [0.19, 1.67]
**Total (95% CI)**		**12863**		**12961**	**100.0%**	**0.99 [0.93, 1.05]**
Total events	1642		1696			

Heterogeneity: Tau² = 0.00; Chi² = 18.59, df = 19 (P = 0.48); I² = 0%
Test for overall effect: Z = 0.28 (P = 0.78)

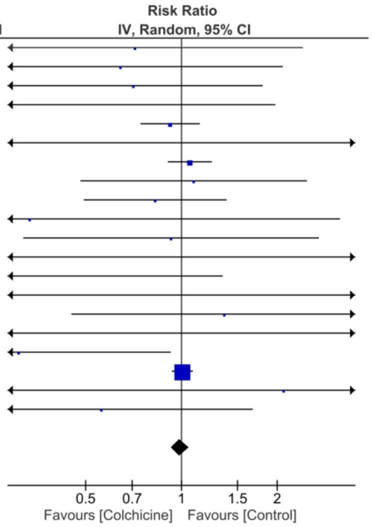

Figure 3. Effect of colchicine on all-cause mortality in COVID-19 patients.

928x493mm (72 x 72 DPI)

Supplementary Material

Colchicine for the treatment of COVID-19 patients: a rapid systematic review and meta-analysis of randomized controlled trials

This supplemental material has been provided by the authors to give readers additional information about their work.

For peer review only

Table S1. PRISMA checklist.

Section and Topic	Item #	Checklist item	Reported on page #
**TITLE**			
Title	1	Identify the report as a systematic review.	1
**ABSTRACT**			
Abstract	2	See the PRISMA 2020 for Abstracts checklist.	3
**INTRODUCTION**			
Rationale	3	Describe the rationale for the review in the context of existing knowledge.	4
Objectives	4	Provide an explicit statement of the objective(s) or question(s) the review addresses.	4
**METHODS**			
Eligibility criteria	5	Specify the inclusion and exclusion criteria for the review and how studies were grouped for the syntheses.	5
Information sources	6	Specify all databases, registers, websites, organisations, reference lists and other sources searched or consulted to identify studies. Specify the date when each source was last searched or consulted.	5
Search strategy	7	Present the full search strategies for all databases, registers and websites, including any filters and limits used.	Suppl.
Selection process	8	Specify the methods used to decide whether a study met the inclusion criteria of the review, including how many reviewers screened each record and each report retrieved, whether they worked independently, and if applicable, details of automation tools used in the process.	5
Data collection process	9	Specify the methods used to collect data from reports, including how many reviewers collected data from each report, whether they worked independently, any processes for obtaining or confirming data from study investigators, and if applicable, details of automation tools used in the process.	5
Data items	10a	List and define all outcomes for which data were sought. Specify whether all results that were compatible with each outcome domain in each study were sought (e.g. for all measures, time points, analyses), and if not, the methods used to decide which results to collect.	5
	10b	List and define all other variables for which data were sought (e.g. participant and intervention characteristics, funding sources). Describe any assumptions made about any missing or unclear information.	5
Study risk of bias assessment	11	Specify the methods used to assess risk of bias in the included studies, including details of the tool(s) used, how many reviewers assessed each study and whether they worked independently, and if applicable, details of automation tools used in the process.	5,6
Effect measures	12	Specify for each outcome the effect measure(s) (e.g. risk ratio, mean difference) used in the synthesis or presentation of results.	6
Synthesis methods	13a	Describe the processes used to decide which studies were eligible for each synthesis (e.g. tabulating the study intervention characteristics and comparing against the planned groups for each synthesis (item #5)).	6

For peer review only

Section and Topic	Item #	Checklist item	Reported on page #
	13b	Describe any methods required to prepare the data for presentation or synthesis, such as handling of missing summary statistics, or data conversions.	6
	13c	Describe any methods used to tabulate or visually display results of individual studies and syntheses.	6
	13d	Describe any methods used to synthesize results and provide a rationale for the choice(s). If meta-analysis was performed, describe the model(s), method(s) to identify the presence and extent of statistical heterogeneity, and software package(s) used.	6
	13e	Describe any methods used to explore possible causes of heterogeneity among study results (e.g. subgroup analysis, meta-regression).	6
	13f	Describe any sensitivity analyses conducted to assess robustness of the synthesized results.	6
Reporting bias assessment	14	Describe any methods used to assess risk of bias due to missing results in a synthesis (arising from reporting biases).	6
Certainty assessment	15	Describe any methods used to assess certainty (or confidence) in the body of evidence for an outcome.	NA
**RESULTS**			
Study selection	16a	Describe the results of the search and selection process, from the number of records identified in the search to the number of studies included in the review, ideally using a flow diagram.	6, Figure 1
	16b	Cite studies that might appear to meet the inclusion criteria, but which were excluded, and explain why they were excluded.	Figure 1
Study characteristics	17	Cite each included study and present its characteristics.	6,7, Table 1
Risk of bias in studies	18	Present assessments of risk of bias for each included study.	7, Figure 2
Results of individual studies	19	For all outcomes, present, for each study: (a) summary statistics for each group (where appropriate) and (b) an effect estimate and its precision (e.g. confidence/credible interval), ideally using structured tables or plots.	Figures, suppl.
Results of syntheses	20a	For each synthesis, briefly summarise the characteristics and risk of bias among contributing studies.	7,8
	20b	Present results of all statistical syntheses conducted. If meta-analysis was done, present for each the summary estimate and its precision (e.g. confidence/credible interval) and measures of statistical heterogeneity. If comparing groups, describe the direction of the effect.	7,8
	20c	Present results of all investigations of possible causes of heterogeneity among study results.	7
	20d	Present results of all sensitivity analyses conducted to assess the robustness of the synthesized results.	7
Reporting	21	Present assessments of risk of bias due to missing results (arising from reporting biases) for each synthesis assessed.	7

Section and Topic	Item #	Checklist item	Reported on page #
biases			
Certainty of evidence	22	Present assessments of certainty (or confidence) in the body of evidence for each outcome assessed.	NA
**DISCUSSION**			
Discussion	23a	Provide a general interpretation of the results in the context of other evidence.	8-10
	23b	Discuss any limitations of the evidence included in the review.	9
	23c	Discuss any limitations of the review processes used.	9
	23d	Discuss implications of the results for practice, policy, and future research.	9,10
**OTHER INFORMATION**			
Registration and protocol	24a	Provide registration information for the review, including register name and registration number, or state that the review was not registered.	4
	24b	Indicate where the review protocol can be accessed, or state that a protocol was not prepared.	4
	24c	Describe and explain any amendments to information provided at registration or in the protocol.	NA
Support	25	Describe sources of financial or non-financial support for the review, and the role of the funders or sponsors in the review.	10
Competing interests	26	Declare any competing interests of review authors.	10
Availability of data, code and other materials	27	Report which of the following are publicly available and where they can be found: template data collection forms; data extracted from included studies; data used for all analyses; analytic code; any other materials used in the review.	10

For peer review only

Table S2. Search strategy for electronic databases.

**MEDLINE (PubMed)**	("colchicine"[MeSH Terms] OR "colchicine"[tiab]) AND ("COVID-19"[MeSH Terms] OR "COVID-19"[tiab] OR "SARS-CoV-2"[tiab] OR "coronavirus disease 2019"[tiab]) AND ("randomized controlled trial"[pt] OR "controlled clinical trial"[pt] OR "randomized"[tiab] OR "placebo"[tiab] OR "drug therapy"[Subheading] OR "therapeutics"[tiab] OR "treatment"[tiab] OR "intervention"[tiab] OR "clinical trial"[pt] OR "placebo-controlled trial"[tiab])
**Embase**	'colchicine'/exp OR 'colchicine':ab,ti AND ('COVID-19'/exp OR 'COVID-19':ab,ti OR 'SARS-CoV-2':ab,ti OR 'coronavirus disease 2019':ab,ti) AND ('clinical trial'/exp OR 'randomized controlled trial'/exp OR 'placebo'/exp OR 'double-blind procedure'/exp OR 'single-blind procedure'/exp OR 'clinical trial phase III'/exp OR 'clinical trial phase IV'/exp OR 'randomization'/exp OR 'placebo-controlled study'/exp OR 'crossover procedure'/exp OR 'factorial design'/exp OR 'control group'/exp)
**The Cochrane Library**	(("Colchicine"[Mesh]) OR ("colchicine"[tiab])) AND (("COVID-19"[Mesh]) OR ("SARS-CoV-2"[Mesh]) OR ("Coronavirus Disease 2019"[Mesh]) OR ("COVID-19"[tiab]) OR ("SARS-CoV-2"[tiab]) OR ("coronavirus disease 2019"[tiab]))

For peer review only

Table S3. Characteristics on included trials.

Sr No	Author, year	Country	Sample size	Age*	Sex, n (%)	Population	Intervention	Comparator	Immunization Status
1	Absalón-Aguilar 2021	Mexico	116	53 (44–62)	Male: 76 (65.5)<br>Female: 40 (35.5)	Hospitalized COVID-19 adult patients with severe disease	1.5 mg of colchicine at the time of the recruitment in the study and 0.5 mg BID to complete 10 days of treatment	Placebo	NR
2	Alsultan 2021	Syria	49 (35 were eligible as 14 received budesonide)	-	-	Hospitalized, moderate-to-severe ARDS, adult patients with either a positive PCR test of COVID-19 virus in specimens taken from the respiratory tracts or a negative PCR test but with clinical signs and symptoms of viral illness along with findings on chest CT compatible with COVID-19 infection	1.5 mg of colchicine followed by 0.5 mg after an hour on day 1, then 0.5 mg twice daily for the next 4 days	Supportive care only	NR
3	Cecconi 2022	Spain	239	65.1 ± 16.0	Male: 141 (59)<br>Female: 98 (41)	Hospitalized moderate-to-severe COVID-19 patients	5 days of oral colchicine: 1 mg loading dose and then 0.5 mg/day	Placebo	NR

For peer review only

4	Deftereos 2020	Greece	105	64 (54-76)	Male: 61 (58.1)<br>Female: 44 (41.9)	Hospitalized adult COVID-19 patients	The loading dose consisted of 1.5 mg of colchicine followed by 0.5 mg of colchicine 60 minutes later. The maintenance dosage was 0.5 mg colchicine twice daily until hospital discharge or a maximum of 21 days.	No colchicine	NR
5	Diaz 2021	Argentina	1279	61.8 ± 14.6	Male: 830 (64.9)<br>Female: 449 (35.1)	Hospitalized severe COVID-19 patients	Colchicine was administered orally in a loading dose of 1.5 mg followed by 0.5 mg orally within 2 hours of the initial dose and 0.5 mg orally twice a day for 14 days or discharge, whichever occurred first.	Usual care with no colchicine	NR
6	Dorward 2022	UK	2293 (156 received colchicine)	-	-	Non-hospitalized COVID-19 patients	Colchicine 500 µg daily for 14 days	Usual care	Both vaccinated and unvaccinated population
7	Eikelboom 2022 (inpatients)	11 countries	2611	Colchicine: 56.1 ± 16.7<br>Control: 56.0 ± 16.0	Colchicine: Male: 762 (58.4), Female: 542 (31.6)<br>Control: Male: 796 (60.9), Female: 511 (39.1)	Hospitalized COVID-19 patients	Colchicine 1.2 mg followed by 0.6 mg 2h later and then 0.6 mg twice daily in tablet form for 28 days	Usual care	Both vaccinated and unvaccinated population

For peer review only - http://bmjopen.bmj.com/site/about/guidelines.xhtml

8	Eikelboom 2022 (outpatients)	11 countries	3917	Colchicine: 45.0 ± 13.7 Control: 45.0 ± 13.3	Colchicine: Male: 1173 (60.5%) Female 766 (39.5%) Control: Male: 1177 (60·6%) Female 765 (39.4%)	Non-hospitalized COVID-19 patients	Oral colchicine 0.6 mg twice daily for 3 days and then 0.6 mg once daily for 25 days	Usual care	Both vaccinated and unvaccinated population
9	Gaitan-Duarte 2021	Bogota, Colombia	314	Colchicine + Rosuvastatin: 56.1 ± 13.2 Control: 55.3 ± 12.3	Colchicine: Male: 103 (68), Female: 50 (32) Standard care: Male 113 (70.2), Female 48 (29.8)	Hospitalized adult COVID-19 patients	Colchicine plus rosuvastatin (0.5 mg and 40 mg for 14 days)	Standard of care	NR
10	Gorial 2022	Iraq	160	-	-	Hospitalized moderate-to-severe COVID-19 patients	Colchicine 1 mg daily orally for 7 days, then 0.5 mg daily for another 7 days	Standard treatment	NR
11	Jalal 2022	Iraq	80	-	Colchicine: Male: 21 (52.5), Female: 19 (47.5) Control: Male: 19 (47.5), Female: 21 (52.5)	Patients with mild, moderate, or severe COVID-19 infection; either hospitalized or at home	Colchicine 0.5 mg twice daily for 14 days	Standard treatment	NR
12	Kasiri 2023	Iran	106	54.62 ± 13.92	Male: 49 (46.2) Female: 57 (53.8)	Hospitalized adult severe COVID-19 patients	Colchicine 2 mg loading dose followed by 0.5 mg twice daily for 7 days	Placebo	NR
13	Lopes 2021	Brazil	75	Colchicine group: 54.5 (42.5–64.5) Placebo: 55.0 (42.0–67.0)	Colchicine: Male 19 (53), Female: 17 (47) Placebo: Male: 14 (39), Female: 22 (61)	Hospitalized moderate-to-severe COVID-19 patients	Colchicine 0.5 mg thrice daily for 5 days, then 0.5mg twice daily for 5 days	Placebo	NR

For peer review only

14	Mostafaie, unpublished	Iran	120	-	-	Hospitalized COVID-19 patients aged ≥ 10 years	Colchicine plus herbal phenolic monoterpene	Standard treatment	NR
15	Pascual-Figal 2021	Spain	103	51 ± 12	Male: 54 (52.4) Female: 49 (47.6)	Hospitalized adult COVID-19 patients	Colchicine 1.5 mg loading dose, followed by 0.5 mg BID for one week and 0.5 mg per day for 28 days	Standard treatment	NR
16	Perricone 2023	Italy	152	Colchicine: 69.1 ± 13.1 Control: 67.9 ± 15	Colchicine: Male: 47 (61), Female: 30 (39) Control: Male: 50 (67), Female: 25 (33)	Hospitalized adult COVID-19 patients	Colchicine 0.5 mg three times a day if weight was less than 100 kg or 1 mg twice a day if weight was more than 100 kg for a maximum of 30 days or until hospital discharge	Standard treatment	Unvaccinated population only
17	Pimenta Bonifácio 2022	Brazil	30	Colchicine: 56.22 ± 11.6 Control: 46.11 ± 10.6	Colchicine: Male: 10 (71), Female: 4 (29) Control: Male: 11 (69), Female 5 (31)	Hospitalized moderate-to-severe COVID-19 patients	Colchicine 0.5 mg every 8 hours for three days, followed by 0.5mg 2 complete four weeks of treatment	Standard of care	Unvaccinated population only
18	Pourdowlat 2021	Iran	165	54.72 ± 15.03	Male: 93 (63) Female: 72 (37)	Non-hospitalized moderate-to-severe COVID-19 patients	A 3-day course of 0.5 mg colchicine followed by a 12-day course of 1 mg colchicine	Standard of care	NR

For peer review only

19	Rahman 2022	Bangladesh	299	47 (35–55)	Male: 190 (64.2) Female: 106 (35.8)	Hospitalized moderate COVID-19 patients	1.2 mg of colchicine on day 1 followed by daily treatment with 0.6 mg of colchicine for 13 days	Placebo	NR
20	RECOVERY 2021	UK, Indonesia, Nepal	11340	63.4 ± 13·8	Colchicine: Male: 3897 (69), Female: 1713 (31) Control group: Male: 4012 (70), Female: 1718 (30)	Hospitalized moderate-to-severe adult COVID-19 patients	Colchicine 1 mg followed by 500 µg 12h later and then 500 µg twice a day by mouth or nasogastric tube for 10 days in total or until discharge	Standard of care	NR
21	Salehzadeh 2022	Iran	100	Colchicine: 56.56 ± 17.16 Placebo: 55.56 ± 16.38	Male: 41 (41) Female 59 (59)	Hospitalized moderate COVID-19 patients	Colchicine 1 mg daily for six days	Placebo	NR
22	Sunil Naik 2023	India	122	-	-	Hospitalized moderate COVID-19 patients	Colchicine 0.5 mg BID	Standard care	NR
23	Tardif 2021	Brazil, Canada, Greece, South Africa, Spain, and the USA	4488	54·0 (47·0–61·0)	Colchicine: Males: 997 (44.6), Females: 1238 (55.4) Control: Males: 1070 (47.5), Female: 1183 (52.5)	Non-hospitalized COVID-19 patients aged ≥ 40 years	Colchicine 0·5 mg BID for the first 3 days and then once per day for 27 days	Placebo	NR

For peer review only

*Data reported as mean ± SD and median (IQR)

BID, twice daily; ARDS, acute respiratory distress syndrome; PCR, polymerase chain reaction: NR, Not reported

For peer review only

Supplementary Figure S1. Funnel plot for all-cause mortality.

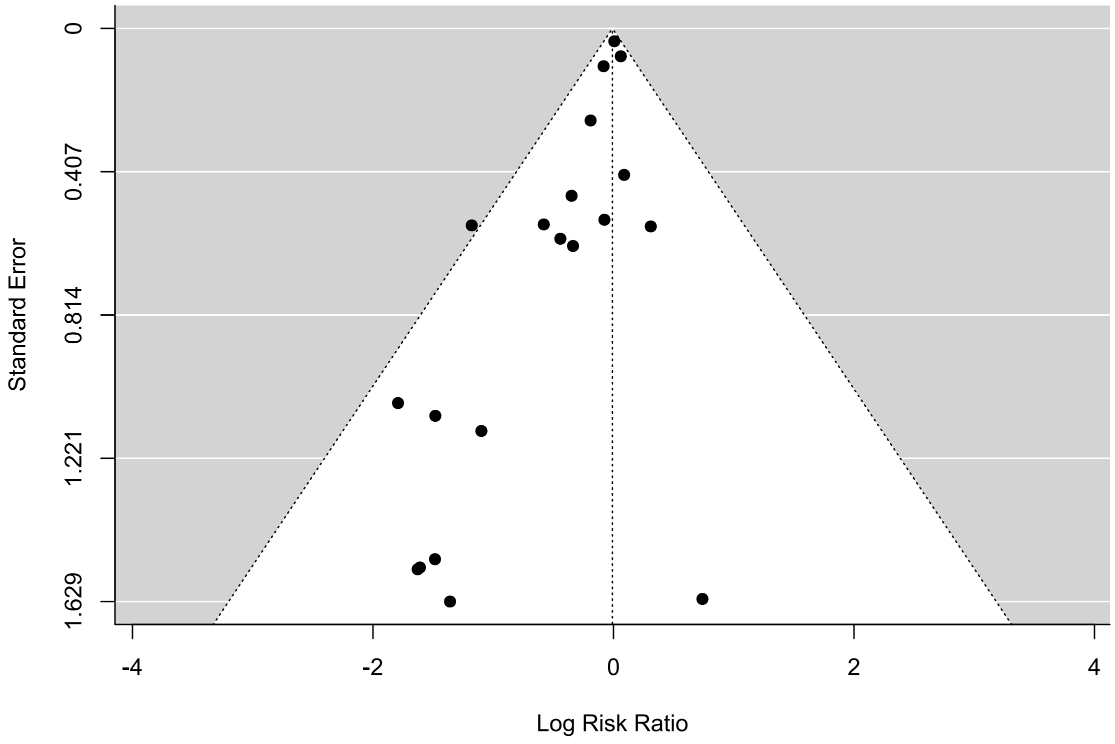

eview only

Supplementary Figure S2. Subgroup analysis of the effect of colchicine on the risk of mortality.

Study or Subgroup	Colchicine Events	Colchicine Total	Control Events	Control Total	Weight	Risk Ratio IV, Random, 95% CI
**1.6.1 Hospitalized**						
Absalon-Aguilar 2021	4	56	6	60	0.3%	0.71 [0.21, 2.40]
Alsultan 2021	3	14	7	21	0.3%	0.64 [0.20, 2.07]
Cecconi 2022	7	119	10	120	0.4%	0.71 [0.28, 1.79]
Deftereos 2020	1	55	4	50	0.1%	0.23 [0.03, 1.97]
Diaz 2021	131	640	142	639	8.3%	0.92 [0.75, 1.14]
Eikelboom 2022 (Inpatient)	264	1304	249	1307	15.3%	1.06 [0.91, 1.24]
Gaitan-Duarte 2021	22	153	28	161	1.4%	0.83 [0.50, 1.38]
Gorial 2022	1	80	3	80	0.1%	0.33 [0.04, 3.14]
Kasiri 2023	6	55	6	51	0.3%	0.93 [0.32, 2.69]
Lopes 2021	0	36	2	36	0.0%	0.20 [0.01, 4.03]
Mostafaie 2021	1	60	6	60	0.1%	0.17 [0.02, 1.34]
Pascual-Figal 2021	0	52	2	51	0.0%	0.20 [0.01, 3.99]
Perricone 2022	7	77	5	75	0.3%	1.36 [0.45, 4.11]
Pimenta-Bonifacio 2022	0	14	2	16	0.0%	0.23 [0.01, 4.36]
Rahman 2022	4	146	13	146	0.3%	0.31 [0.10, 0.92]
RECOVERY 2021	1173	5610	1190	5730	71.8%	1.01 [0.94, 1.08]
Sunil 2023	1	62	0	43	0.0%	2.10 [0.09, 50.25]
**Subtotal (95% CI)**		**8533**		**8646**	**99.1%**	**0.98 [0.90, 1.06]**
Total events	1625		1675			
Heterogeneity: Tau² = 0.00; Chi² = 16.79, df = 16 (P = 0.40); I² = 5%						
Test for overall effect: Z = 0.47 (P = 0.64)						
**1.6.2 Non-hospitalized**						
Dorward 2022	0	156	1	120	0.0%	0.26 [0.01, 6.25]
Eikelboom 2022 (Outpatient)	12	1939	11	1942	0.6%	1.09 [0.48, 2.47]
Tardif 2021	5	2235	9	2253	0.3%	0.56 [0.19, 1.67]
**Subtotal (95% CI)**		**4330**		**4315**	**0.9%**	**0.82 [0.43, 1.55]**
Total events	17		21			
Heterogeneity: Tau² = 0.00; Chi² = 1.45, df = 2 (P = 0.48); I² = 0%						
Test for overall effect: Z = 0.61 (P = 0.54)						
**Total (95% CI)**		**12863**		**12961**	**100.0%**	**0.99 [0.93, 1.05]**
Total events	1642		1696			
Heterogeneity: Tau² = 0.00; Chi² = 18.59, df = 19 (P = 0.48); I² = 0%						
Test for overall effect: Z = 0.28 (P = 0.78)						
Test for subgroup differences: Chi² = 0.30, df = 1 (P = 0.59), I² = 0%						

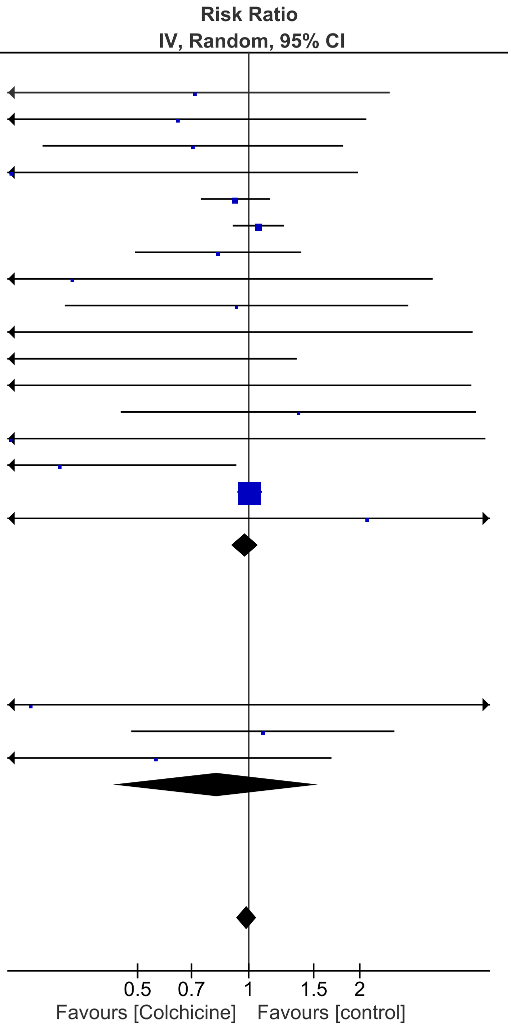

For peer review only - http://bmjopen.bmj.com/site/about/guidelines.xhtml

Supplementary Figure S3. Forest plot of the effect of colchicine on the risk of mechanical ventilation and ICU admission.

	Colchicine		Control			Risk Ratio
**Study or Subgroup**	**Events**	**Total**	**Events**	**Total**	**Weight**	**IV, Random, 95% CI**
**1.2.1 Risk of mechanical ventilation**						
Cecconi 2022	5	119	10	120	12.5%	0.50 [0.18, 1.43]
Deftereos 2020	1	55	5	50	4.0%	0.18 [0.02, 1.50]
Kasiri 2023	6	55	6	51	12.2%	0.93 [0.32, 2.69]
Pascual-Figal 2021	0	52	2	51	2.1%	0.20 [0.01, 3.99]
Perricone 2022	4	77	3	75	7.6%	1.30 [0.30, 5.61]
Rahman 2022	2	146	4	146	6.0%	0.50 [0.09, 2.69]
RECOVERY 2021	259	3815	228	3962	36.4%	1.18 [0.99, 1.40]
Tardif 2021	11	2235	21	2253	19.2%	0.53 [0.26, 1.09]
**Subtotal (95% CI)**		**6554**		**6708**	**100.0%**	**0.75 [0.48, 1.18]**
Total events	288		279			
Heterogeneity: Tau² = 0.14; Chi² = 11.69, df = 7 (P = 0.11); I² = 40%						
Test for overall effect: Z = 1.23 (P = 0.22)						
**1.2.2 ICU admission**						
Cecconi 2022	11	119	14	120	37.4%	0.79 [0.38, 1.67]
Kasiri 2023	12	55	9	51	34.7%	1.24 [0.57, 2.69]
Lopes 2021	2	36	4	36	7.8%	0.50 [0.10, 2.56]
Pascual-Figal 2021	2	52	4	51	7.7%	0.49 [0.09, 2.56]
Perricone 2022	1	77	4	75	4.4%	0.24 [0.03, 2.13]
Rahman 2022	2	146	5	146	7.9%	0.40 [0.08, 2.03]
**Subtotal (95% CI)**		**485**		**479**	**100.0%**	**0.77 [0.49, 1.22]**
Total events	30		40			
Heterogeneity: Tau² = 0.00; Chi² = 3.70, df = 5 (P = 0.59); I² = 0%						
Test for overall effect: Z = 1.10 (P = 0.27)						

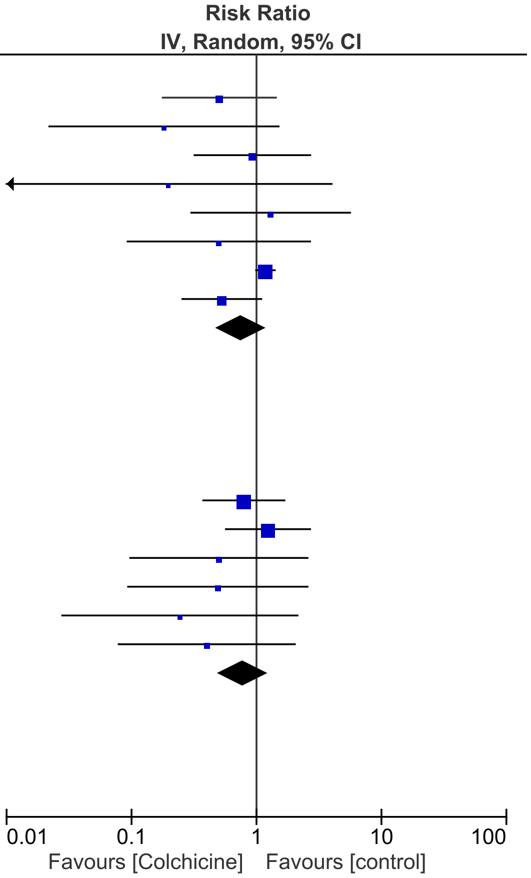

eview only

Supplementary Figure S4. Forest plot of the effect of colchicine on the risk of hospitalization.

Study or Subgroup	Colchicine Events	Colchicine Total	Control Events	Control Total	Weight	Risk Ratio IV, Random, 95% CI
Eikelboom 2022 (Outpatient)	62	1939	61	1942	39.7%	1.02 [0.72, 1.44]
Pourdowlat 2021	5	102	18	100	15.5%	0.27 [0.11, 0.71]
Tardif 2021	101	2235	128	2253	44.8%	0.80 [0.62, 1.03]
**Total (95% CI)**		**4276**		**4295**	**100.0%**	**0.74 [0.48, 1.16]**
Total events	168		207			

Heterogeneity: Tau² = 0.10; Chi² = 6.69, df = 2 (P = 0.04); I² = 70%
Test for overall effect: Z = 1.30 (P = 0.19)

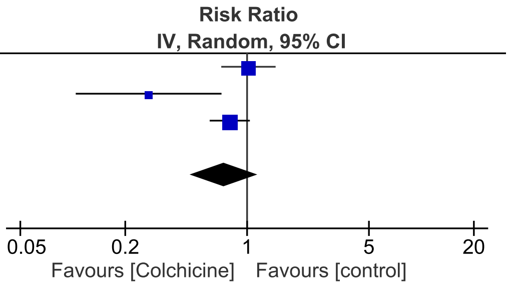

For peer review only

Supplementary Figure S5. Forest plot of the effect of colchicine on the length of hospital stay.

Study or Subgroup	Colchicine Mean	Colchicine SD	Colchicine Total	Control Mean	Control SD	Control Total	Weight	Mean Difference IV, Random, 95% CI
Absalon-Aguilar 2021	7.916	4.26	56	8.33	4.07	60	13.2%	-0.41 [-1.93, 1.10]
Jalal 2022	18.4	5.81	40	24.24	8.63	40	9.3%	-5.84 [-9.06, -2.62]
Lopes 2021	7	2.96	36	9.33	3.7	36	13.1%	-2.33 [-3.88, -0.78]
Mostafaie 2021	6.39	2.59	54	4.17	1.34	59	14.4%	2.22 [1.45, 2.99]
Pascual-Figal 2021	6.6	3.86	52	5.76	4.89	51	12.8%	0.84 [-0.86, 2.54]
Perricone 2022	14.1	10.4	77	14.7	8.1	75	9.9%	-0.60 [-3.56, 2.36]
Rahman 2022	10.67	5.93	146	10	6.67	146	13.3%	0.67 [-0.78, 2.12]
Salehzadeh 2022	6.28	2.51	50	8.12	2.66	50	14.1%	-1.84 [-2.85, -0.83]
**Total (95% CI)**			**511**			**517**	**100.0%**	**-0.70 [-2.30, 0.89]**

Heterogeneity: Tau² = 4.44; Chi² = 67.53, df = 7 (P < 0.00001); I² = 90%
Test for overall effect: Z = 0.86 (P = 0.39)

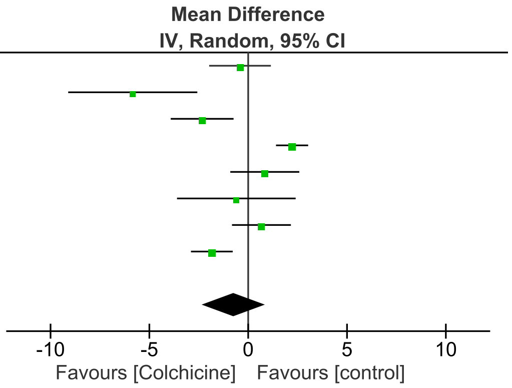

For peer review only

Supplementary Figure S6. Forest plot of the effect of colchicine on the rate of no recovery.

	Colchicine		Control			Risk Ratio	Risk Ratio
**Study or Subgroup**	**Events**	**Total**	**Events**	**Total**	**Weight**	**IV, Random, 95% CI**	**IV, Random, 95% CI**
Dorward 2022	42	156	306	1145	25.3%	1.01 [0.76, 1.33]	
Gorial 2022	12	80	32	80	14.8%	0.38 [0.21, 0.67]	
Kasiri 2023	7	55	9	51	8.5%	0.72 [0.29, 1.79]	
Pourdowlat 2021	25	102	40	100	20.1%	0.61 [0.40, 0.93]	
RECOVERY 2021	1709	5610	1698	5730	31.3%	1.03 [0.97, 1.09]	
**Total (95% CI)**		**6003**		**7106**	**100.0%**	**0.77 [0.56, 1.05]**	
Total events	1795		2085				

Heterogeneity: Tau² = 0.08; Chi² = 17.39, df = 4 (P = 0.002); I² = 77%

Test for overall effect: Z = 1.65 (P = 0.10)

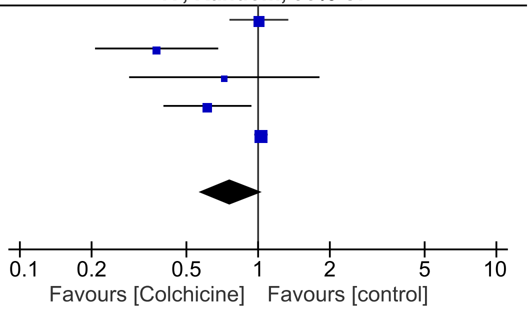

For peer review only

PRISMA 2020 checklist

Section and Topic	Item #	Checklist item	Reported on page #
**TITLE**			
Title	1	Identify the report as a systematic review.	1
**ABSTRACT**			
Abstract	2	See the PRISMA 2020 for Abstracts checklist.	3
**INTRODUCTION**			
Rationale	3	Describe the rationale for the review in the context of existing knowledge.	4
Objectives	4	Provide an explicit statement of the objective(s) or question(s) the review addresses.	4
**METHODS**			
Eligibility criteria	5	Specify the inclusion and exclusion criteria for the review and how studies were grouped for the syntheses.	5
Information sources	6	Specify all databases, registers, websites, organisations, reference lists and other sources searched or consulted to identify studies. Specify the date when each source was last searched or consulted.	5
Search strategy	7	Present the full search strategies for all databases, registers and websites, including any filters and limits used.	Suppl.
Selection process	8	Specify the methods used to decide whether a study met the inclusion criteria of the review, including how many reviewers screened each record and each report retrieved, whether they worked independently, and if applicable, details of automation tools used in the process.	5
Data collection process	9	Specify the methods used to collect data from reports, including how many reviewers collected data from each report, whether they worked independently, any processes for obtaining or confirming data from study investigators, and if applicable, details of automation tools used in the process.	5
Data items	10a	List and define all outcomes for which data were sought. Specify whether all results that were compatible with each outcome domain in each study were sought (e.g. for all measures, time points, analyses), and if not, the methods used to decide which results to collect.	5
	10b	List and define all other variables for which data were sought (e.g. participant and intervention characteristics, funding sources). Describe any assumptions made about any missing or unclear information.	5
Study risk of bias assessment	11	Specify the methods used to assess risk of bias in the included studies, including details of the tool(s) used, how many reviewers assessed each study and whether they worked independently, and if applicable, details of automation tools used in the process.	5,6
Effect measures	12	Specify for each outcome the effect measure(s) (e.g. risk ratio, mean difference) used in the synthesis or presentation of results.	6
Synthesis methods	13a	Describe the processes used to decide which studies were eligible for each synthesis (e.g. tabulating the study intervention characteristics and comparing against the planned groups for each synthesis (item #5)).	6

For peer review only

Section and Topic	Item #	Checklist item	Reported on page #
	13b	Describe any methods required to prepare the data for presentation or synthesis, such as handling of missing summary statistics, or data conversions.	6
	13c	Describe any methods used to tabulate or visually display results of individual studies and syntheses.	6
	13d	Describe any methods used to synthesize results and provide a rationale for the choice(s). If meta-analysis was performed, describe the model(s), method(s) to identify the presence and extent of statistical heterogeneity, and software package(s) used.	6
	13e	Describe any methods used to explore possible causes of heterogeneity among study results (e.g. subgroup analysis, meta-regression).	6
	13f	Describe any sensitivity analyses conducted to assess robustness of the synthesized results.	6
Reporting bias assessment	14	Describe any methods used to assess risk of bias due to missing results in a synthesis (arising from reporting biases).	6
Certainty assessment	15	Describe any methods used to assess certainty (or confidence) in the body of evidence for an outcome.	NA
**RESULTS**			
Study selection	16a	Describe the results of the search and selection process, from the number of records identified in the search to the number of studies included in the review, ideally using a flow diagram.	6, Figure 1
	16b	Cite studies that might appear to meet the inclusion criteria, but which were excluded, and explain why they were excluded.	Figure 1
Study characteristics	17	Cite each included study and present its characteristics.	6,7, Table 1
Risk of bias in studies	18	Present assessments of risk of bias for each included study.	7, Figure 2
Results of individual studies	19	For all outcomes, present, for each study: (a) summary statistics for each group (where appropriate) and (b) an effect estimate and its precision (e.g. confidence/credible interval), ideally using structured tables or plots.	Figures, suppl.
Results of syntheses	20a	For each synthesis, briefly summarise the characteristics and risk of bias among contributing studies.	7,8
	20b	Present results of all statistical syntheses conducted. If meta-analysis was done, present for each the summary estimate and its precision (e.g. confidence/credible interval) and measures of statistical heterogeneity. If comparing groups, describe the direction of the effect.	7,8
	20c	Present results of all investigations of possible causes of heterogeneity among study results.	7
	20d	Present results of all sensitivity analyses conducted to assess the robustness of the synthesized results.	7
Reporting	21	Present assessments of risk of bias due to missing results (arising from reporting biases) for each synthesis assessed.	7

For peer review only

Section and Topic	Item #	Checklist item	Reported on page #
biases			
Certainty of evidence	22	Present assessments of certainty (or confidence) in the body of evidence for each outcome assessed.	NA
**DISCUSSION**			
Discussion	23a	Provide a general interpretation of the results in the context of other evidence.	8-10
	23b	Discuss any limitations of the evidence included in the review.	9
	23c	Discuss any limitations of the review processes used.	9
	23d	Discuss implications of the results for practice, policy, and future research.	9,10
**OTHER INFORMATION**			
Registration and protocol	24a	Provide registration information for the review, including register name and registration number, or state that the review was not registered.	4
	24b	Indicate where the review protocol can be accessed, or state that a protocol was not prepared.	4
	24c	Describe and explain any amendments to information provided at registration or in the protocol.	NA
Support	25	Describe sources of financial or non-financial support for the review, and the role of the funders or sponsors in the review.	10
Competing interests	26	Declare any competing interests of review authors.	10
Availability of data, code and other materials	27	Report which of the following are publicly available and where they can be found: template data collection forms; data extracted from included studies; data used for all analyses; analytic code; any other materials used in the review.	10

For peer review only
